# Synthesis and In Vivo Antiarrhythmic Activity Evaluation of Novel Scutellarein Analogues as Voltage-Gated Nav1.5 and Cav1.2 Channels Blockers

**DOI:** 10.3390/molecules28217417

**Published:** 2023-11-03

**Authors:** Wei Yang, Wenping Wang, Song Cai, Peng Li, Die Zhang, Jinhua Ning, Jin Ke, Anguo Hou, Linyun Chen, Yunshu Ma, Wenbin Jin

**Affiliations:** 1 Key Laboratory of External Drug Delivery System and Preparation Technology in Universities of Yunnan, Yunnan University of Chinese Medicine, Kunming 650500, Chinazhangdie422@163.com (D.Z.);; 2 Faculty of Chinese Materia Medica, Yunnan University of Chinese Medicine, Kunming 650500, China; 3 Department of Anatomy and Histology, Shenzhen University Medical School, Shenzhen 518060, China; 4 School of Food and Drug, Shenzhen Polytechnic, Shenzhen 518000, China; 5 State Key Laboratory of Chemical Biology, The Hong Kong Polytechnic University, Hung Hom, Kowloon, Hong Kong SAR, China; 6 Drug Discovery and Department of Applied Biology, The Hong Kong Polytechnic University, Hung Hom, Kowloon, Hong Kong SAR, China; 7 Chemical Technology, The Hong Kong Polytechnic University, Hung Hom, Kowloon, Hong Kong SAR, China

**Keywords:** antiarrhythmic, scutellarein derivatives, Nav1.5 channel blockers, Cav1.2 channel blockers, electrophysiology

## Abstract

Malignant cardiac arrhythmias with high morbidity and mortality have posed a significant threat to our human health. Scutellarein, a metabolite of Scutellarin which is isolated from *Scutellaria altissima* L., presents excellent therapeutic effects on cardiovascular diseases and could further be metabolized into methylated forms. A series of 22 new scutellarein derivatives with hydroxyl-substitution based on the scutellarin metabolite in vivo was designed, synthesized via the conjugation of the scutellarein scaffold with pharmacophores of FDA-approved antiarrhythmic medications and evaluated for their antiarrhythmic activity through the analyzation of the rat number of arrhythmia recovery, corresponding to the recovery time and maintenance time in the rat model of barium chloride-induced arrhythmia, as well as the cumulative dosage of aconitine required to induce VP, VT, VF and CA in the rat model of aconitine-induced arrhythmia. All designed compounds could shorten the time of the arrhythmia continuum induced by barium chloride, indicating that 4′-hydroxy substituents of scutellarein had rapid-onset antiarrhythmic effects. In addition, nearly all of the compounds could normalize the HR, RR, QRS, QT and QTc interval, as well as the P/T waves’ amplitude. The most promising compound **10e** showed the best antiarrhythmic activity with long-term efficacy and extremely low cytotoxicity, better than the positive control scutellarein. This result was also approved by the computational docking simulation. Most importantly, patch clamp measurements on Nav1.5 and Cav1.2 channels indicated that compound **10e** was able to reduce the INa and ICa in a concentration-dependent manner and left-shifted the inactivation curve of Nav1.5. Taken together, all compounds were considered to be antiarrhythmic. Compound **10e** even showed no proarrhythmic effect and could be classified as Ib Vaughan Williams antiarrhythmic agents. What is more, compound **10e** did not block the hERG potassium channel which highly associated with cardiotoxicity.

## 1. Introduction

Sudden cardiac death caused by malignant cardiac arrhythmias including ventricular tachycardia, ventricular fibrillation and cardiac arrest with high morbidity and mortality over recent decades have posed a significant threat to our human health, accounting for 15–20% of all deaths [1]. One major cause for arrythmias is the aberrant sinus node firing of electrical signals mediated by the voltage-gated ion channels including Na, K and Ca, etc., and *β*-adrenergic stimulation [2]. Based on the different mechanisms of action, antiarrhythmic drugs could be classified into four classes such as Na channel blockers (category I), *β*-receptor blockers (category II), K channel blockers (category III) and Ca channel blockers (category IV) according to the Vaughan Williams’ classification [3]. At present, antiarrhythmic drugs were the predominant clinical treatments, but almost all of them had proarrhythmic effects at different degrees [4,5]. In traditional Chinese medicine, arrhythmia could be classified as “palpitations” [6]. Anti-arrhythmic Chinese medicine formulas had advantages of safety in the prevention and treatment of arrhythmias, for instance, Wen Xin Granules and Shensong Yangxin Capsules [7,8,9,10,11]. Therefore, screening from natural products in Chinese materia medica acting as antiarrhythmic drugs mediated by multiple ion channels seemed to be an approach of importance [12,13].

Traditional Chinese medicine (TCM) has been widely used to treat cardiovascular and cerebrovascular diseases with unique clinical benefits including multi-target pharmacological functions and fewer side effects [14,15,16]. Particularly, a panel of dietary flavonoids have been reported [17,18,19]. Scutellarin isolated from *Scutellaria altissima* L. has been reported to present excellent therapeutic effects on cardiovascular diseases and inflammation [20,21]. Recent investigations also have provided evidence for its multiple pharmacological properties such as anti-cancer, anticonvulsive, antioxidative, anti-inflammatory, hepatoprotective, antibacterial, antiviral and neuroprotective activity [22,23,24,25]. Meanwhile, the long-term regimen of a high-dose scutellarin was also proved to be a sufficient margin of safety suggested by acute and subacute toxicity studies [26]. These investigations confirmed that scutellarin could be a promising drug candidate for the treatment of cardiovascular diseases.

**Figure 1:**
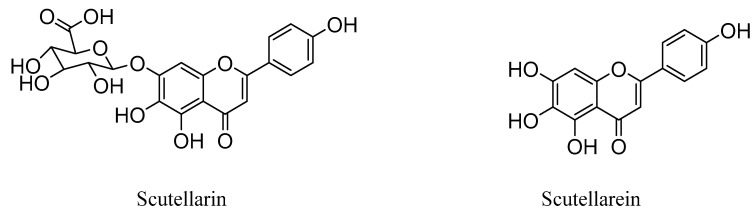
Chemical structures of scutellarin and scutellarein.

Interestingly, studies have revealed that the prodrug scutellarin transformed into scutellarein in vivo, which could further be metabolized into methylated forms, as shown in Figure 1 [27,28,29,30]. Therefore, scutellarein analogues bearing substitutes at the hydroxyl group could be more metabolic stable and have a much higher bioavailability in vivo, compared with scutellarin only with 0.4% oral bioavailability in a beagle dog [31,32].

**Figure 2:**
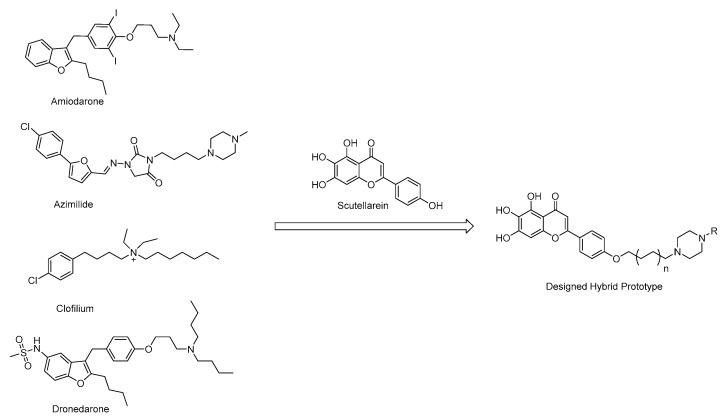
Chemical structures of antiarrhythmic drugs approved by FDA and Design strategy of new scutellarein derivatives.

Based on the structural analysis results of antiarrhythmic drugs approved by the FDA, such as Amiodarone, Dronedarone, Azimilide, etc., it was found that they all have a nitrogen atom-containing group, which easily transforms to quaternary ammonium salts with an ability to bind to the peptide anion on the cardiomyocyte membrane (Figure 2).

In order to find the Structure–Activity relationships (SAR) of scutellarein derivatives, herein we described the synthesis and in vivo antiarrhythmic evaluation of a novel series of scutellarein analogues via semi-synthetic methods. By adopting a hybrid prototype combining scutellarein and tertiary amines, 22 novel compounds were designed and synthesized. Then, the antiarrhythmic activities of these compounds were evaluated by the detection of the rat number of arrhythmia recovery, corresponding to the recovery time, maintenance time, as well as ECG parameters such as heart rate (HR), QT interval (QT), QRS interval, QTc interval (QTc) and the amplitude of P and T waves in the rat model of barium chloride-induced arrhythmia. Meanwhile, the cumulative dosage of aconitine required to induce ventricular premature beats (VP), ventricular tachycardia (VT), ventricular fibrillation (VF) and cardiac arrest (CA) in the rat model of aconitine-induced arrhythmia were also determined. Furthermore, to reveal the antiarrhythmic mechanism of these compounds, we examined their effects on voltage-gated ion channels including Nav1.5 and Cav1.2. Nav1.5 is the dominant voltage-gated sodium channel in the heart, responsible for type 3 long QT syndrome (LQT3) and Brugada syndrome (BrS). The dysfunction of the Nav channels is generally associated with multiple pathophysiological syndromes, exemplified by arrhythmias, seizures, epilepsy, autism and Parkinson’s disease [33]. We have also confirmed that regulation of the Nav channels could reverse peripheral neuropathic pain [34,35,36]. Among the Cav channels, the Cav1 family belonging to the L-type channels require strong depolarization for activation to form the dominant Ca^2+^ influx route in vascular cells. The drug-associated inhibition of hERG potassium channels could cause long QT syndrome and trigger fatal arrhythmias such as torsades de pointes (Tdp) [37]. In recent years, a variety of non-anti-arrhythmic drugs such as terfenadine, asimizole and astemizole have been removed by the FDA, mainly due to fatal arrhythmias caused by the inhibition of hERG potassium channels, also known as Kv11.1 [38]. Therefore, we also reported here the effects of these compounds on hERG potassium channels to illustrate whether it displayed cardiotoxicity or not. 

## 2. Results

### 2.1. Chemistry

**Scheme 1:**
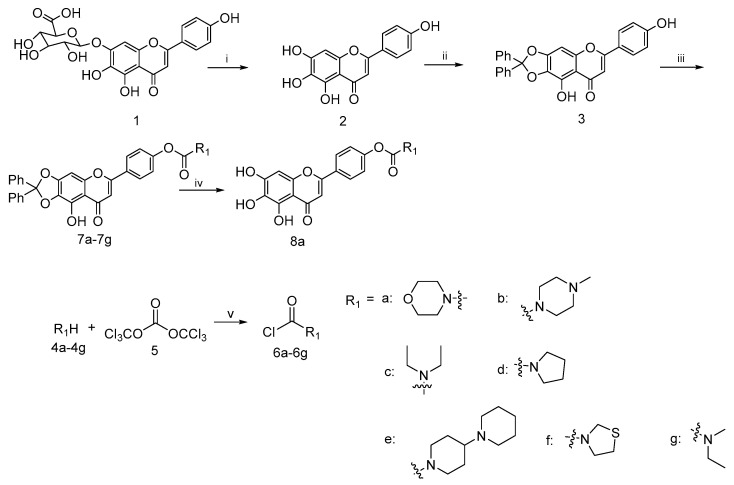
Reagents and conditions: (**i**) H_2_SO_4_, EtOH, N_2_, reflux, 8 h; (**ii**) dichlorodiphenylmethane, diphenyl ether, 175 °C, 30 min; (**iii**) **6a**–**6g**, K_2_CO_3_, KI, DMF, 25 °C, 24 h; (**iv**) H_2_, 10%Pd/C, THF/EtOH, 12 h; (**v**) Et_3_N, DCM, N_2_, 0 °C, 5 h.

Based on the previous reports, a concise synthetic route for the preparation of an intermediate compound with good yields was depicted in Scheme 1. Starting from the commercially available scutellarin (**1**), the conversion to compound **2** by refluxing with 6N H_2_SO_4_ in alcohol followed by subsequent protection with dichlorodiphenylmethane using a diphenyl ether as the solvent and esterification with a wide range of fresh prepared acid chloride **6a–6h** using K_2_CO_3_ and KI as the catalysts in *N,N*-dimethylformamide (DMF), we successfully afforded the target compounds **7a**–**7h** in three steps with reasonably good yields. The deprotection of **7a** in the presence of a catalytic amount of 10% palladium on carbon at the room temperature gave the compound **8a** in good yield [39].

**Scheme 2:**
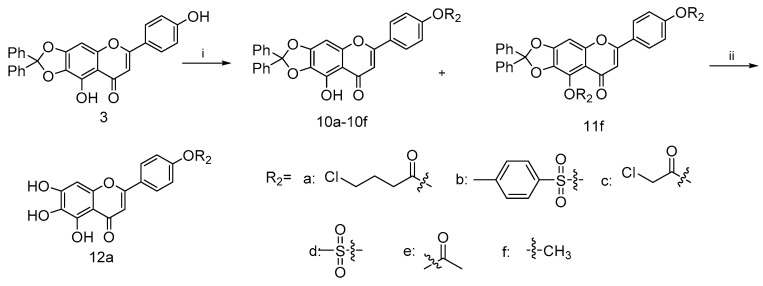
Reagents and conditions: (**i**) for **9a–9e**, Et_3_N, DMF, 25 °C, 24 h; for **10f** and **11f**, K_2_CO_3_, KI, DMF, 25 °C, 24 h (**ii**) H_2_, 10%Pd/C, THF/EtOH, 12 h.

Similarly, the coupling of intermediate **3** with various acid chloride **9a–9e** in the presence of Et_3_N gave **10a**–**10e**, as shown in Scheme 2 [40,41]. Such efficient syntheses of 4′-substituted scutellarein derivatives allowed the rapid construction of target compounds for subsequent biological studies. In addition, scutellarein derivatives **10f** and **11f** were obtained in moderate yields as the consequences of the methylation of **3**, respectively. Subsequently, the palladium-on-carbon-catalyzed facile hydrogenative deprotection of **10a** in THF/EtOH solution with an H_2_ atmosphere at room temperature produced **12a**.

**Scheme 3:**
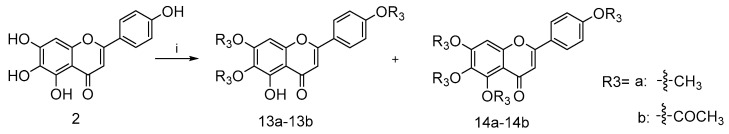
Reagents and conditions: (**i**) for **13a** and **14a**, NaH, pyridine, 25 °C, 12 h; for **13b** and **14b**, DMAP, pyridine, 25 °C, 12 h.

Furthermore, compounds **13a**–**13b** with a methyl group and **14a**–**14b** with a acetyl group were prepared as shown in Scheme 3 in order to explore the importance of the 1-phenolic hydroxyl group. The treatment of intermediate **2** with methyl iodide or acetic anhydride gave the desired compound **13a**–**13b** and **14a**–**14b,** respectively.

### 2.2. Biological Activity

According to the published method, the antiarrhythmic activity of the synthetic compounds was examined in a barium chloride- or aconitine-induced arrhythmia rat model [42]. Normal electrocardiogram (ECG) waves could be observed before intraperitoneal anesthesia with pentobarbital sodium (50 mg/kg) and before a sublingual vein injection of barium chloride (4 mg/kg). The aftermath of the administration of arrhythmogen including BaCl_2_ and aconitine was the occurrence of VP, followed by aggravating VT and VF, eventually with CA resulting in the cardiac mortality of the rat.

#### 2.2.1. Antiarrhythmic Effects of Scutellarein Derivatives on BaCl_2_-Induced Arrhythmia in Rats

The criterion for antiarrhythmic activity was the gradual disappearance of arrhythmia and the restoration of the sinus rhythm. The observation was performed for 35 min. A total of 22 scutellarein derivatives administrated at a dose of 8 mg/kg were evaluated for antiarrhythmic activity by monitoring the recovery period from arrhythmia and the duration of recovery maintenance after treatments. BaCl_2_ (4 mg/kg) administered into the sublingual vein evokes heart rhythm irregularities including extrasystoles, conduction blocks and bradycardia which lead to long QT syndrome. The negative control group such as the NS or DMSO group receiving no treatment except for the administration of arrhythmogen showed arrhythmia, suggesting that the barium chloride-induced arrhythmia rat model was set up successfully. Both the lead compound scutellarein at a dose of 8 mg/kg and the FDA-approved antiarrhythmic drug Verapamil (Ver) at a dose of 2 mg/kg acted as positive controls for comparison. 

These results shown in Table 1 suggest that nearly all of the scutellarein analogues exhibited better antiarrhythmic activity against barium chloride-induced arrhythmia than that of the positive control scutellarein, especially shortening the time of the arrhythmia continuum, indicating that 4′-hydroxy substituents of scutellarein had rapid-onset antiarrhythmic effects (*p* < 0.001; Table 1). Meanwhile, the onset time of our synthetic compounds showed no difference with that of Ver. Compounds **8a** and **12a,** without the diphenyl protecting group, even displayed weaker antiarrhythmic activity with a shorter duration of efficacy, compared with the corresponding compounds **7a** and **10a**. At the same time, the number of rats with a duration of efficacy larger than 20 min was decreased by 1–2, respectively. This might be ascribed to the steric hindrance of the diphenyl protecting group hampering the metabolism of the scutellarein analogues, which leads to an extension of the duration of efficacy, suggesting that the 6,7- and 4′-hydroxyl groups of scutellarein would not be beneficial for antiarrhythmic activity. In addition, it was worthy to notice that compounds **7b**, **10a**, **10e**, **11f** and **13b** with the 4′-hydroxyl substituents and the diphenyl group exhibited better antiarrhythmic effects than that of the positive control scutellarein, subject to the speeding up of the onset of drugs. Compound **10e** even had a longer duration of efficacy (*p* < 0.05) and a shorter time to onset (*p* < 0.001) than the lead compound scutellarein. In addition, the number of rats with a duration of efficacy larger than 20 min also increased, further proving the aforementioned conclusion. However, the methylated compounds **13a** and **14a** have a much shorter duration time than the acetylated compounds **13b** and **14b**. This is mainly due to the fact that acetylated compounds could be easier to metabolize. 

Generally, arrhythmias could give rise to long QT syndrome. The results, as shown in the Table 2, further prove this situation. The administration of BaCl_2_ prior to the investigated compounds (pre-dose group) caused a significant extension in QT duration, whereas the injection of the tested compounds (post-dose group) could sharply reduce the duration of QT and even made no difference with the normal group, with the exception of the negative control groups (*p* < 0.001), as well as Ver, **10b**, **11f** and **12a** (*p* < 0.05). Moreover, an injection of the tested compounds could all significantly alter the QT intervals compared with that of the NS group (*p* < 0.05, *p* < 0.01 or *p* < 0.001). Based on the conclusion that the synthesized compounds could restore the QT interval, it was suggested that the synthesized compounds may be targeted to treat sudden cardiac death caused by ventricular tachycardia. 

**Figure 3:**
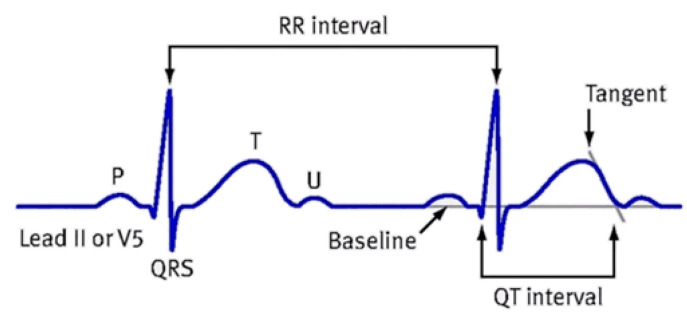
Measurement diagram of QTc.

To diminish the influence of the heart rate on the QT interval, the QTc interval was also investigated. The QTc, shown in Figure 3, was calculated according to the formula of Bazzett: QTc = QT/√RR. 

The results of the QTc are listed in Table 3. It was worth noting that an injection of BaCl_2_ (pre-dose) could alter the QTc interval, heart rate (HR) and QRS interval. However, after the administration of synthetic compounds, the QTc interval showed no difference with that of the Normal group, with the exception of compound **13a** and **14a** (*p* < 0.05), which suggests that our compound could restore the QTc value after the administration of BaCl_2_. Still worse, the positive control Ver and negative control NS could not restore the QTc. In addition, the results related to the HR, RR and QRS interval also suggested that nearly all of the compounds could normalize the HR, RR and QRS interval after the administration of BaCl_2_ (refer to S2–S4 in the Supporting Information). 

Compared with the normal group, the pre-dose group, referring to the BaCl_2_-treated group, significantly decreased the P wave and T wave amplitude, as shown in Table 4 and Table 5. After one injection with our compounds, the P wave and T wave amplitude was nearly restored. 

Next, by the analysis of the antiarrhythmic activity of a small library of scutellarein analogues that we constructed, the potent compound **10e** was screened out and it was further determined whether it has dose-dependent effects on BaCl_2_-induced arrhythmia in rats. It was found that increasing the administration dose of **10e** from 2 mg/kg to 8 mg/kg could diminished the onset time of efficacy and extended the duration efficacy. The results are summarized in Table 6, which displays that the number of rats with a duration of efficacy more than 20 min increased from 0 to 5, as well as that the number of rats with a duration of efficacy less than 10 min decreased from 3 to 1. However, the antiarrhythmic effects decreased when the administration dose of **10e** was 16 mg/kg. Overall, compound **10e** could sharply decrease the onset time in a dose-dependent manner. 

#### 2.2.2. Antiarrhythmic Effects of Scutellarein Analogues on Aconitine-Induced Arrhythmia in Rats

VP were followed by VT and VF appearing in all treated rats with the administration of aconitine. The administration of compound **10e** at a concentration of 8 mg/kg prior to aconitine caused a significant increase in the cumulative dosage of aconitine required to induce VF and CA compared with the NS and DMSO groups, respectively (*p* < 0.05 or *p* < 0.01; Table 7). In addition, an intraperitoneal injection of scutellarein gave rise to a notable improvement in the cumulative dosage of aconitine needed to evoke VP, VT and VF compared with the negative control groups and the Ver positive control group, whereas the pro-drug of scutellarein, named scutellarin, showed much weaker antiarrhythmic effects than scutellarein against VP, VT, VF as well as CA induced by aconitine, indicating that scutellarein was a promising lead compound for the investigation of an antiarrhythmic drug.

#### 2.2.3. The Influence of the Compounds on Normal Rat ECG

**Figure 4:**
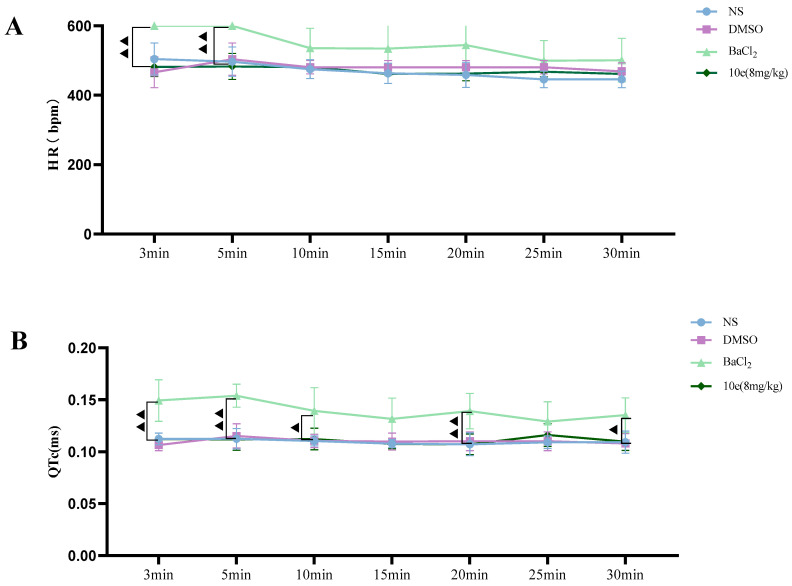

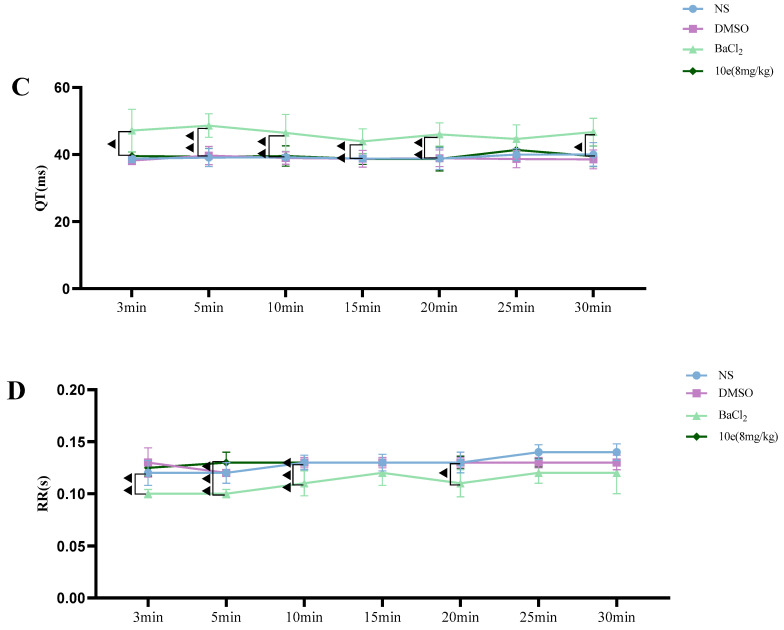
Compound **10e**-induced alterations of HR (**A**), QTc-Bazett’s (**B**), QT (**C**) and RR (**D**) in anesthetized rats. Significantly different between compound **10e** group and BaCl_2_-treated group in QT and QTc value, ▲ *p* < 0.05, ▲▲ *p* < 0.01, ▲▲▲ *p* < 0.001.

Compared with the NS and DMSO groups, compound **10e** administered into the sublingual vein at a dose of 8 mg/kg did not show any significant difference in the HR, RR, QT and QTc interval, suggesting that this compound did not have any proarrhythmic effect and could be classified as Ib Vaughan Williams antiarrhythmic agents. In addition, after the administration of **10e**, no statistically significant changes regarding QTc and QT were observed in 30 min. However, the injection of BaCl_2_ altered the QT, QTc intervals, HR and RR markedly in the 3rd and 5th min post-injection of anesthesia, as shown in Figure 4. 

#### 2.2.4. Cytotoxicity Test of Compound **10e** against Normal Cell

**Figure 5:**
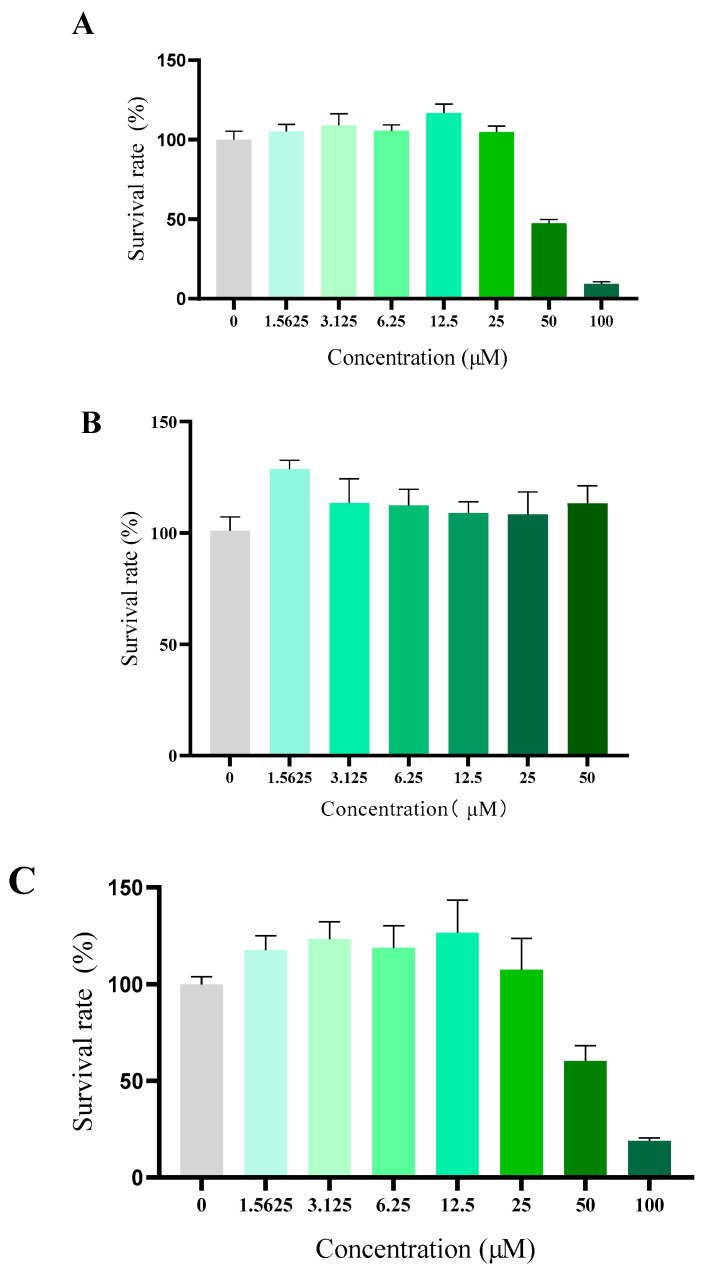
Cytotoxicity of compound **10e** towards HFLI cell lines (**A**), HEK293 cell lines (**B**) and H9c2 cell lines (**C**).

Compound toxicity has always been a great concern for the development of antiarrhythmic drug in clinic use. Although the daily oral treatment of scutellarin at a dose of 500 mg/kg for 30 days to rats was proved to be a sufficient margin of safety suggested by in vivo acute and subacute toxicity studies, whether scutellarin displayed high toxicity towards eukaryotic cells or not remained still unclear [26]. To verify the cytotoxicity of **10e** in vitro, human fetal lung fibroblast I (HFL-I), HEK293 and H9c2 cells were used, respectively. As shown in Figure 5, compound **10e** exhibited negligible cytotoxicity against HFL-I and H9c2 cells lines with a greater than 100% cell viability at a concentration of 25 μM, which is approximately the concentration in the patch clamp study. Microscopic investigations of H9c2 cell lines also indicated that there were no morphological changes after incubation with compound **10e** at the concentration of 25 μM, displaying relatively low cytotoxicity. (Refer to Supplementary S8).

#### 2.2.5. Molecular Docking with Nav1.5 and Cav1.2 Channels

**Figure 6:**
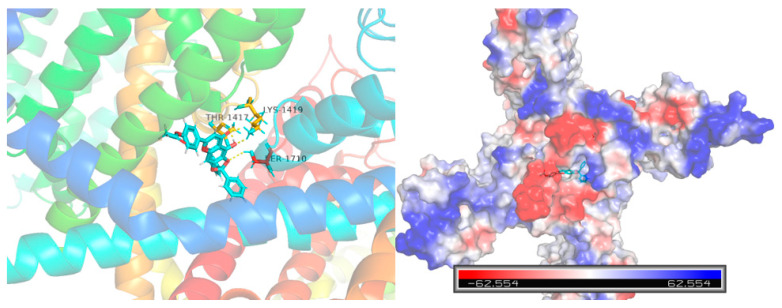
Virtual molecular docking of compounds **10e** with Nav1.5 protein.

Computational molecular docking was conducted to gain more insights into the molecular interaction details between compound **10e** and the Nav1.5 protein (ID: 6LQA) (Figure 6). The results revealed that the highest docking score positioned compound **10e** into the substrate-binding site of the Nav1.5 protein (ID: 6LQA). The warheads of the 2,2-diphenyl protection group of compound **10e** were well-situated in the central cavity of the pore domain of the ion channel of the Nav1.5 protein, interacting with the amino acid residues nearby. A conventional hydrogen-bonding interaction was predicted to occur between the amide group of THR-1417, LYS-1419 and the hydroxyl group of **10e**, as well as between the amide group of SER-1710 and the hydroxyl group of **10e**. 

Furthermore, the docking score of scutellarein and a Nav1.5 protein was 5.775, which was lower than that of compound **10e** (6.8012), suggesting that compound **10e** had better binding energy with the Nav1.5 protein in accordance with the aforementioned antiarrhythmic results. This indicated that **10e** should be worthy of further study.

**Figure 7:**
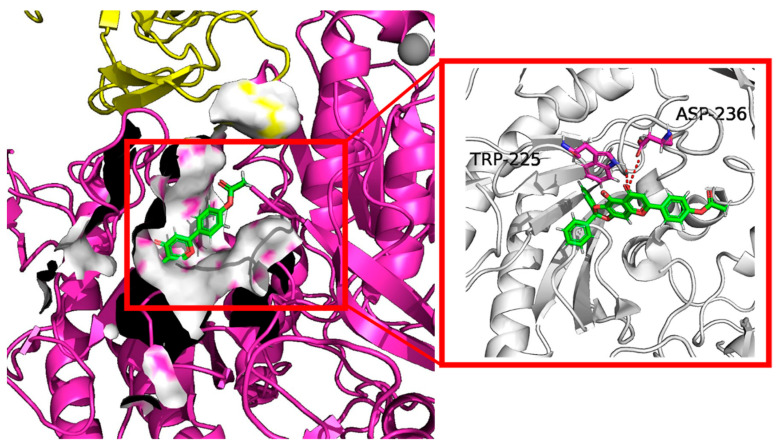
Virtual molecular docking of compounds **10e** with Cav1.2 protein.

The docking results shown in Figure 7 also suggested that the highest docking score positioned compound **10e** into the substrate-binding site of Cav1.2 (ID: 8FD7). A hydrogen-bonding interaction was predicted by the PLIP website to occur between residues TRP-225, ASP236 and the hydroxyl group of **10e**. The length of the hydrogen bonds was less than 3 pm, showing a strong physicochemical force.

#### 2.2.6. Patch Clump Recordings

**Figure 8:**
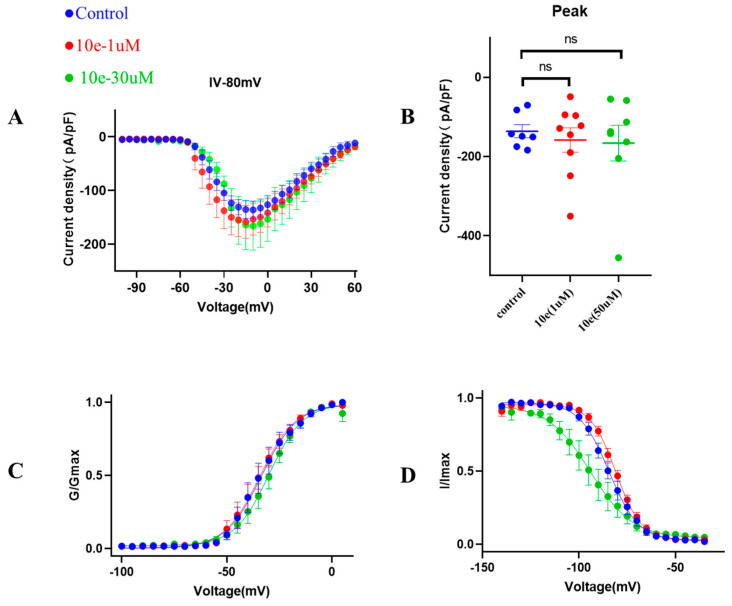
Effect of compound **10e** on voltage-dependent sodium current (I_Na_) activation and inactivation. (**A**) Current density of Nav1.5 mediated by control (DMSO) and various concentration of compound **10e**. (**B**) Scatter distribution plot of experimental data (**C**) Voltage-dependent activation (G/Gmax) and (**D**) inactivation (I/Imax) of the I_Na_ in the absence (blue circles) or presence of 1 μM compound **10e** (red circles) or presence of 30 μM compound **10e** (green circles).

Next, to provide additional mechanistic insights into the effects of compound **10e** on the biophysical properties of the I_Na_, we investigated the voltage-dependent activation of the I_Na_ in the absence or presence of compound **10e** (1 μM and 30 μM). It was clear that compound **10e** did not alter the current density of the Nav1.5 channel and the voltage-dependency for I_Na_ activation (Figure 8). Figure 8C presents the comparable activation curves (G/G_MAX_, V_1/2_ = −33.6 ± 1.1 mV in control vs. −34.2 ± 0.9 mV in the presence of compound **10e** at the concentration of 1 μmol vs. −30.1 ± 0.9 mV in the presence of compound **10e** at the concentration of 30 μmol, *n* = 8, *p* > 0.05). However, the steady-state inactivation curve (Figure 8D) was left-shifted in the presence of compound **10e** (I/I_MAX_, V_1/2_ = −84.4 ± 0.6 mV in control vs. −80.8 ± 0.4 mV in the presence of compound **10e** at the concentration of 1 μmol vs. −94.1 ± 1.7 mV in the presence of compound **10e** at the concentration of 30 μmol, *n* = 8, *p* < 0.05), indicating that compound **10e** may enhance the inactivation of the Nav1.5 channel.

**Figure 9:**
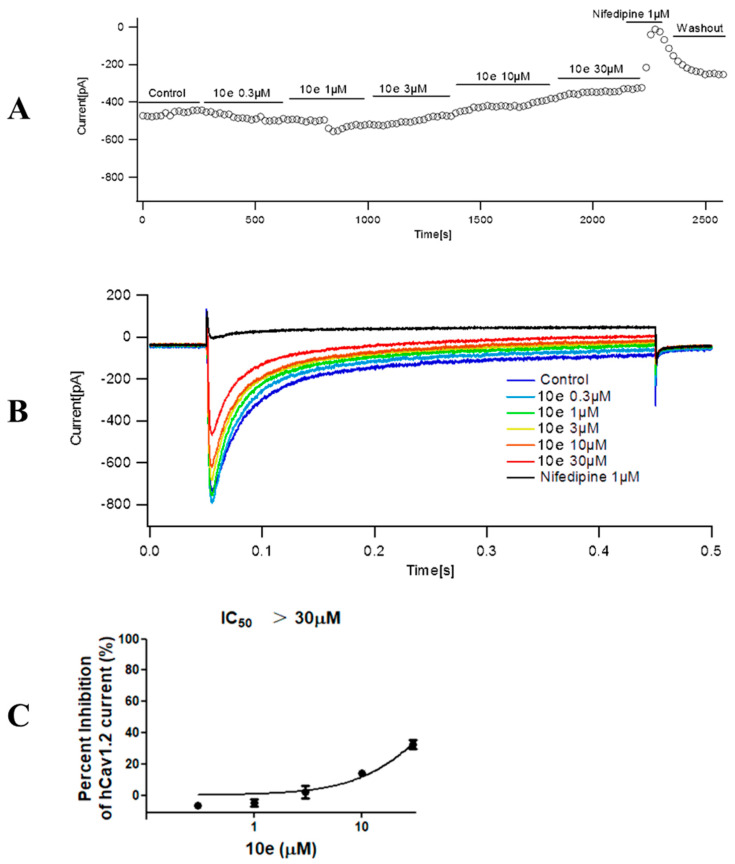
Effect of compound **10e** on the peak calcium current (I_Ca_) amplitude. (**A**) Time course effect of different concentrations of compound **10e** (0.3–30 μM) on the peak I_Ca_ at V_H_ = −80 mV. (**B**) Representative I_Ca_ traces in the control group and in the presence of compound **10e** at a holding potential (V_H_) = −80 mV. (**C**) Dose−response curve of compound **10e** on the I_Ca_ amplitude when cells were maintained at V_H_ = −80 mV. *n* = 3 cells.

Compound **10e** was found to reduce the I_Ca_ peak amplitude in a concentration-dependent manner. In addition, the maximum effect for each tested concentration was reached quickly, as is displayed in the time course effect of compound **10e** (Figure 9). When cells were exposed to increasing concentrations of compound **10e** (from 0.3 to 30 μM), a concentration–response curve of I_Ca_ peak inhibition was obtained. The interaction of drugs with the calcium channel depends on the channel state, for instance, closed, open and inactivated. Thus, we composed a concentration–response curve of compound **10e** in cells expressing human Cav1.2 at a holding potential (V_H_) of −80 mV, which favor a partially inactivated state. We found that there was an evident dependency of the resting membrane potential in the potency of compound **10e** to block I_Ca._ Once the cell membrane was held at a V_H_ of −80 mV, compound **10e** (30 μM) reduced approximately 32.58 ± 5.16% of the I_Ca_ amplitude. This effect was partially reversible after washout. Interestingly, when the V_H_ was set at −80 mV, Nifedipine (1 μM) attenuated almost 97.93 ± 0.96% of the I_Ca_ amplitude. The IC_50_ of compound **10e** for I_Ca_ inhibition at a V_H_ of −80 mV was undefined due to the solubility dilemma. These findings indicated that the compound **10e**-dependent inhibition of the I_Ca_ preferentially targets the inactivated state of Cav 1.2 and may partially explain its antiarrhythmic activity.

**Figure 10:**
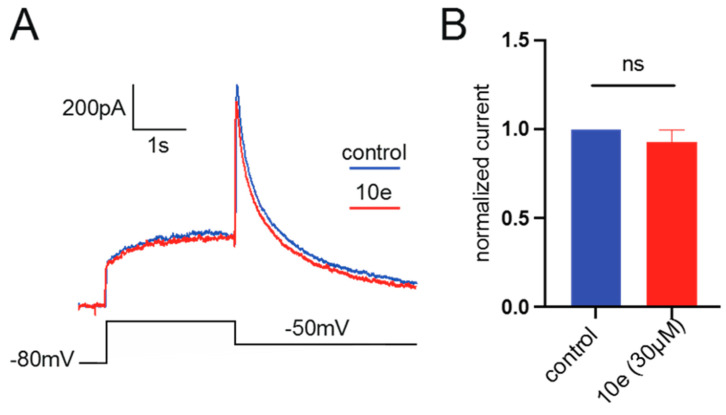
(**A**). Representative traces of current recorded from HEK293 expressing hERG channel by patch clamp technique in the presence or absence of **10e** (30 µM) perfusion. The current was recorded by depolarizing the voltage to +40 mV for 2.5 s from the holding potential at −80 mV, followed by a hyperpolarization step to −50 mV, lasting for 4 s, which elicited the peak tail current of hERG channel. (**B**). Normalized peak tail current of hERG channel, *n* = 4 for each group.

It was found that compound **10e** at the concentration of 30 μM could not reduce the I_kr_ peak amplitude. This suggests that it did not block the hERG potassium channel, indicating that our compound in future drug development could avoid the cardiac side effects (Figure 10).

## 3. Discussion

In this work, based on the concept of a twin drug, a total of 22 novel scutellarein derivatives have been synthesized via the conjugation of the scutellarein scaffold with a pharmacophore of FDA-approved antiarrhythmic medication. The antiarrhythmic effects of these scutellarein derivatives were evaluated by the analyzation of the rat number of arrhythmia recovery, ECG parameters, corresponding recovery time and maintenance time in the rat model of barium chloride-induced arrhythmia, as well as the cumulative dosage of aconitine required to induce VP, VT, VF and CA in the rat model of aconitine-induced arrhythmia. Compounds with 4′-hydroxyl substituents had rapid-onset antiarrhythmic effects. Furthermore, an injection of our compounds could normalize the HR, RR, QRS, QT and QTc interval, as well as the P/T waves’ amplitude. The most promising compound, **10e**, exhibited the best antiarrhythmic activity with long-term efficacy and extremely low cytotoxicity. What is more, compound **10e** showed no proarrhythmic effects and it did not block the hERG potassium channel, which is highly associated with cardiotoxicity. Therefore, it could be classified as an Ib Vaughan Williams antiarrhythmic agent. Importantly, compound **10e** could gently reduce the I_Na_ and I_Ca_ in a concentration-dependent manner and left-shifted the inactivation curve of Nav1.5. However, the IC_50_ of compound **10e** could not be determined due to the poor solubility. At present, a variety of ion channels have been reported to be involved in the action potential of the myocardium, including the sodium, potassium and calcium channels, etc. Maybe compound **10e** also interacts with other kinds of ion channels. However, in cardiomyocytes, which types of ion channels that compound **10e** inhibits to exert such potent antiarrhythmic effects is unclear and needs to be further studied. Altogether, our work indicated that **10e** represented an important scutellarein scaffold targeting voltage-gated ion channels including Nav1.5 and Cav1.2, providing a promising starting point to be further developed as a safe antiarrhythmic medication.

## 4. Materials and Methods

### 4.1. Reagents, Solvents and Chemicals

Scutellarin (**1**) was purchased from Yuanye Biotechnology Co., Ltd. (Shanghai, China). All reagents and solvents were purchased from Aladdin Holdings Group Co., Ltd. (Shanghai, China) and used without further purification unless otherwise stated. The anhydrous solvents were transferred via syringe. All reactions were monitored by TLC by using UV light of 254 nm as the detection wavelength. Chromatographic purifications were carried out on silica gel (160–200 mesh) by using gradient mixtures of petroleum ether and ethyl acetate as eluent. All activity tested compounds were determined by an Agilent 1260 series HPLC to have at least 95% purity. All ^1^H NMR and ^13^C NMR spectra were recorded on Bruker Avance spectrometer. High-resolution mass spectra (HRMS) were obtained on an Agilent 6545 by quadrupole time-of-flight (Q-TOF) mode.

### 4.2. Synthesis of Scutellarein Derivatives

#### 4.2.1. 5,6,7-Trihydroxy-2-(4-hydroxyphenyl)-4H-chromen-4-one (**2**)

To a well-stirred mixture of scutellarin (**1**) (3.0 g, 6.4 mmol) in 95% ethanol (48 mL), solution of concentrated H_2_SO_4_ (18 mL) was added dropwise and the reaction was refluxed for 4 h under nitrogen. After that, excess water (50 mL) was added in portions to the reaction mixture. Meanwhile, the precipitates that appeared were filtered, washed with glacial acetic acid and dried in vacuo to afford scutellarein **2** (1.1 g, 59% yield) as a yellow solid; mp 324−335 °C; ^1^H NMR (400 MHz, CDCl_3_) δ 12.70 (s, 1H), 7.73–7.82 (m, 2H), 7.49–7.59 (m, 4H), 7.28–7.35 (m, 6H), 7.15–7.25 (m, 2H), 6.55 (s, 1H), 6.58 (s, 1H), 3.26–3.32 (m, 2H), 3.21 (q, J = 7.17 Hz, 2H) and 1.09–1.13 (m, 6H). ^13^C NMR (101 MHz, DMSO-d6) δ 182.5, 164.0, 161.5, 153.8, 150.1, 147.5, 129.6, 128.8, 121.9, 116.4, 104.4, 102.7 and 94.3. HRMS(Q-TOF) m/z calculated for C_15_H_10_O_6_ [M + H]^+^: 287.0477 found: 287.0490.

#### 4.2.2. 9-Hydroxy-6-(4-hydroxyphenyl)-2,2-diphenyl-8H-[1,3]dioxolo[4,5-g]chromen-8-one (**3**)

A mixture of scutellarein (**2**) (10.0 g, 34.94 mmol) and dichlorodiphenylmethane (10.0 g, 42.17 mmol) in diphenyl ether (200 mL) was stirred at 180 °C under nitrogen protection for 1.5 h. Then, the resulting dilution was allowed to slowly cool to room temperature. Petroleum ether (400 mL) was added dropwise to the solution to remove the diphenyl ether from the solid that appeared. Then, the filtered solid was purified by column chromatography on silica gel with 25% ethyl acetate in petroleum ether as eluent to afford **3** (10.7 g, 68%) as a yellow solid; mp 357−364 °C; ^1^H NMR (400 MHz, DMSO-d6) δ 7.81–7.93 (m, 2H), 7.46–7.55 (m, 4H), 7.36–7.46 (m, 6H), 6.99 (s, 1H), 6.89 (d, J = 8.80 Hz, 2H) and 6.79 (s, 1H). ^13^C NMR (101 MHz, DMSO-d6) δ 182.9, 164.7, 162.8, 162.2, 153.2, 152.9, 139.3, 130.2, 129.2, 129.2, 129.0, 126.3, 121.1, 118.7, 116.5, 107.4, 103.1, 36.3 and 31.2. HRMS(Q-TOF) m/z calculated for C_28_H_18_O_6_ [M + H]^+^: 451.1103 found: 451.1179.

#### 4.2.3. General Procedure for the Synthesis of Compounds **7a**–**7g**

To a well-stirred mixture of compound **4a**–**4g** (10.3 mmol) in dichlorodiphenylmethane (10 mL) a solution of triphosgene (3.0 g, 10.1 mmol) in DCM (15 mL) was added dropwise at 0 °C and the reaction mixture was stirred for 6–8 h. Then it was allowed to warm slowly to room temperature. After the disappearance of starting material, as indicated by TLC, the reaction mixture was blown by nitrogen and filtered to afford the filtrate **6a**–**6g,** respectively, which could be used directly in the next step.

To a well-stirred solution of compound **3** (3 g, 6.6 mmol) in anhydrous DMF (30 mL) at rt. the aforementioned fresh prepared carbamoyl chloride **6a**–**6g** in the presence of K_2_CO_3_ (1.0 g, 7.2 mmol) and KI (1.0 g, 6.0 mmol) was added. The resulting solution was diluted with ethyl acetate, washed with brine, dried over anhydrous Na_2_SO_4_, filtered and evaporated under reduced pressure. The crude residue was purified by flash column chromatography on silica gel using 50% ethyl acetate in petroleum ether as eluent to afford **7a**–**7g**.

##### 4-(9-Hydroxy-8-oxo-2,2-diphenyl-8H-[1,3]dioxolo[4,5-g]chromen-6-yl)phenyl morpholine-4-carboxylate (**7a**)

This compound (2.2 g, yield 54.0%) was prepared from important immediate compound **3** (3.0 g, 6.6 mmol), DMF (30 mL) and 4-morpholinylcarbonyl chloride **6a** (1.2 g, 8 mmol) according to the general procedure described above. ^1^H NMR (400 MHz, DMSO-d6) δ 12.94 (s, 1H), 7.97–8.19 (m, J = 8.80 Hz, 2H), 7.47–7.55 (m, 5H), 7.39–7.45 (m, 6H), 7.28–7.34 (m, J = 8.80 Hz, 2H), 7.08 (s, 1H), 7.01 (s, 1H), 3.57–3.64 (m, 4H), 3.45–3.57 (m, 2H) and 3.38 (br. s., 2H). ^13^C NMR (101 MHz, DMSO-d6) δ 183.2, 163.5, 154.5, 153.2, 139.2, 130.3, 129.2, 128.3, 126.3, 123.0, 107.6, 22.6 and 19.4. HRMS(Q-TOF) m/z calculated for C_33_H_25_NO_8_ [M + H]^+^: 564.1580 found: 564.1654.

##### 4-(9-Hydroxy-8-oxo-2,2-diphenyl-8H-[1,3]dioxolo[4,5-g]chromen-6-yl)phenyl 4-methylpiperazine-1-carboxylate (**7b**)

This compound (0.4 g, yield 62.6%) was prepared from important immediate compound **3** (0.5 g, 1.1 mmol), DMF (10 mL) and 4-methylpiperazine-1-carbonyl chloride **6b** (0.18 g, 1.3 mmol) according to the general procedure described above. ^1^H NMR (400 MHz, DMSO-d6) δ 13.00 (br. s., 1H), 8.12 (d, J = 8.07 Hz, 2H), 7.53–7.62 (m, 4H), 7.48 (d, J = 5.62 Hz, 6H), 7.36 (d, J = 7.58 Hz, 2H), 7.14 (s, 1H), 7.07 (s, 1H), 3.62 (br. s., 2H), 3.45 (br. s., 2H), 2.41 (br. s., 4H) and 2.25 (s, 3H). ^13^C NMR (101 MHz, METHANOL-d4) δ 166.6, 152.1, 98.3, 94.2, 77.1, 76.5, 76.1, 73.0, 70.3, 50.3, 50.1 and 12.5. HRMS(Q-TOF) m/z calculated for C_34_H_28_N_2_O_7_ [M + H]^+^: 577.1897 found: 577.1964.

##### 4-(9-Hydroxy-8-oxo-2,2-diphenyl-8H-[1,3]dioxolo[4,5-g]chromen-6-yl)phenyl diethylcarbamate (**7c**)

This compound (0.32 g, yield 52.5%) was prepared from important immediate compound **3** (0.5 g, 1.1 mmol), DMF (10 mL) and diethylcarbamic chloride **6c** (0.18 g, 1.3 mmol) according to the general procedure described above. ^1^H NMR (400 MHz, CDCl_3_) δ 12.70 (s, 1H), 7.73–7.82 (m, 2H), 7.49–7.59 (m, 4H), 7.28–7.35 (m, 6H), 7.15–7.25 (m, 2H), 6.55 (s, 1H), 6.58 (s, 1H), 3.26–3.32 (m, 2H), 3.21 (q, J = 7.17 Hz, 2H) and 1.09–1.13 (m, 6H). ^13^C NMR (101 MHz, CHLOROFORM-d) δ 182.99, 163.42,154.44, 153.50, 153.27, 142.30, 139.25, 130.04, 129.48, 128.38, 127.86, 127.43, 126.33, 122.45, 119.22, 107.77, 105.23, 89.56, 42.44, 42.37, 14.22 and 13.11. HRMS(Q-TOF) m/z calculated for C_33_H_27_NO_7_ [M + H]^+^: 550.1788 found: 550.1859.

##### 4-(9-Hydroxy-8-oxo-2,2-diphenyl-8H-[1,3]dioxolo[4,5-g]chromen-6-yl)phenyl pyrrolidine-1-carboxylate (**7d**)

This compound (0.19 g, yield 79.2%) was prepared from important immediate compound **3** (0.2 g, 0.4 mmol), DMF (10 mL) and pyrrolidine-1-carbonyl chloride **6d** (0.13 g, 0.97 mmol) according to the general procedure described above. ^1^H NMR (400 MHz, CHLOROFORM-d) δ 12.69 (s, 1H), 7.70–7.88 (m, 2H), 7.46–7.59 (m, 4H), 7.27–7.37 (m, 6H), 7.20–7.23 (m, 2H), 6.55 (s, 1H), 6.58 (s, 1H), 3.28–3.44 (m, 4H) and 1.17–1.22 (m, 4H). ^13^C NMR (101 MHz, CDCl_3_) δ 183.0, 163.4, 142.3, 139.3, 130.1, 129.5, 128.4, 127.9, 127.4, 126.3, 122.4 and 89.5. HRMS(Q-TOF) m/z calculated for C_33_H_25_NO_7_ [M + Na]^+^: 570.1631 found: 572.1684.

##### 4-(9-Hydroxy-8-oxo-2,2-diphenyl-8H-[1,3]dioxolo[4,5-g]chromen-6-yl)phenyl [1,4′-bipiperidine]-1′-carboxylate (**7e**)

This compound (0.18 g, yield 64.3%) was prepared from important immediate compound **3** (0.2 g, 0.4 mmol), DMF (10 mL) and [1,4′-bipiperidine]-1′-carbonyl chloride **6e** (0.23 g,0.99 mmol) according to the general procedure described above. ^1^H NMR (400 MHz, CDCl_3_) δ ppm 1.12–1.26 (m, 2 H), 1.33–1.42 (m, 2 H), 1.49–1.56 (m, 5 H), 1.82 (d, J = 12.72 Hz, 2 H), 2.35–2.42 (m, 1 H), 2.42–2.50 (m, 4 H), 2.77 (t, J = 12.23 Hz, 1 H), 2.91 (t, J = 12.23 Hz, 1 H), 4.14–4.40 (m, 2 H), 6.48–6.60 (m, 2 H), 7.14–7.23 (m, 2 H), 7.25–7.38 (m, 6 H), 7.45–7.61 (m, 4 H) and 7.69–7.84 (m, 2 H). ^13^C NMR (101 MHz, CDCl_3_) δ 183.0, 163.3, 153.5, 153.3, 142.3, 139.3, 129.5, 128.4, 128.1, 127.5, 126.3, 122.4, 119.2, 107.8, 105.3, 89.5, 62.5, 50.3, 26.0 and 24.5. HRMS(Q-TOF) m/z calculated for C_39_H_36_N_2_O_7_ [M + H]^+^: 645.2523 found: 645.2678.

##### 4-(9-Hydroxy-8-oxo-2,2-diphenyl-8H-[1,3]dioxolo[4,5-g]chromen-6-yl)phenyl thiazolidine-3-carboxylate (**7f**)

This compound (0.05 g, yield 71.4%) was prepared from important immediate compound **3** (0.06 g, 0.1 mmol), DMF (10 mL) and thiazolidine-3-carbonyl chloride **6f** (0.04 g, 0.2 mmol) according to the general procedure described above. ^1^H NMR (400 MHz, CDCl_3_) δ ppm 3.05–3.19 (m, 2 H), 3.84–3.97 (m, 2 H), 4.61 (s, 1 H), 4.68 (s, 1 H), 6.62–6.65 (m, 1 H), 6.66 (s, 1 H), 7.31 (m, J = 8.80 Hz, 2 H), 7.38–7.43 (m, 6 H), 7.58–7.66 (m, 4 H), 7.81–7.93 (m, 2 H) and 12.75 (s, 1 H). ^13^C NMR (101 MHz, CDCl_3_) δ ppm 182.95, 163.17, 153.55, 153.28, 142.31, 139.24, 130.09, 129.48, 128.38, 127.55, 126.34, 122.35, 119.26, 107.79, 105.42 and 89.53. HRMS(Q-TOF) m/z calculated for C_32_H_23_NO_7_S [M + H]^+^:566.5925 found: 566.1265.

##### 4-(9-Hydroxy-8-oxo-2,2-diphenyl-8H-[1,3]dioxolo[4,5-g]chromen-6-yl)phenyl ethyl(methyl)carbamate (**7g**)

This compound (0.16 g, yield 71.4%) was prepared from important immediate compound **3** (0.2 g, 0.4 mmol), DMF (10 mL) and ethyl(methyl)carbamic chloride **6g** (0.12 g, 0.9 mmol) according to the general procedure described above. ^1^H NMR (400 MHz, CDCl_3_) δ 12.67 (s, 1H), 7.77–7.84 (m, 2H), 7.52–7.59 (m, 4H), 7.31–7.36 (m, 6H), 7.21–7.27 (m, J = 8.80 Hz, 2H), 6.52–6.60 (m, 2H), 4.60 (s, 1H), 4.52 (s, 1H), 3.76–3.89 (m, 2H) and 2.99–3.09 (m, 2H). ^13^C NMR (101 MHz, CDCl_3_) δ 183.0, 164.0, 162.6, 153.3, 153.2, 142.4, 139.3, 129.4, 128.4, 128.0, 126.3, 114.5, 107.7, 104.0 and 89.4. HRMS(Q-TOF) m/z calculated for C_32_H_25_NO_7_ [M + H]^+^: 536.1631 found: 536.1702.

#### 4.2.4. Synthesis of 4-(5,6,7-Trihydroxy-4-oxo-4H-chromen-2-yl) phenyl morpholine-4-carboxylate (**8a**)

Compound **7a** (0.3 g, 0.5 mmol) was stirred with 10% Pd/C catalyst (0.03 g, 0.2 mmol) in a mixture solution of EtOH (10 mL) and THF (10 mL) for 12 h under hydrogen atmosphere, followed by filtration over celite for removal of catalyst. Then the filtrate was evaporated in vacuo and the resulting residue was purified by column chromatography on silica gel using 20% ethyl acetate in petroleum ether as eluent to afford **8a** (0.15 g, 75.0% yield). ^1^H NMR (400 MHz, DMSO-d6) δ ppm 3.59–3.77 (m, 8 H), 6.58 (s, 1 H), 6.86 (s, 1 H), 7.24–7.41 (m, 2 H) and 7.97–8.16 (m, 2 H). HRMS(Q-TOF) m/z calculated for C_20_H_17_NO_8_ [M + H]^+^: 400.1184 found: 400.1215.

#### 4.2.5. General Procedure for the Synthesis of Compounds **10a**–**10e**

To a mixture solution of compound **3** and Et_3_N in anhydrous DCM (20 mL) at 0 °C, commercially available carbonyl chlorides **9a**–**9e** (0.29 mmol) were added dropwise, stirring well over 12 h. Then, the reaction was diluted with water (20 mL). The collected organic phase was dried over anhydrous Na_2_SO_4_, filtered and concentrated under reduced pressure. Purification of the crude residue was performed by flash column chromatography on silica gel using 25% ethyl acetate in petroleum ether as eluent to afford the desired compound **10a**–**10f**.

##### 4-(9-Hydroxy-8-oxo-2,2-diphenyl-8H-[1,3]dioxolo[4,5-g]chromen-6-yl)phenyl 4-chlorobutanoate (**10a**)

This compound (0.34 g, yield 34.7%) was prepared from important immediate compound **3** (0.8 g, 1.7 mmol), Et_3_N (0.35 g, 3.4 mmol) and 4-chlorobutyrylchloride **9a** (0.3 g, 2.1 mmol) according to the general procedure described above. ^1^H NMR (400 MHz, DMSO-d6) δ 12.98 (s, 1H), 8.08–8.23 (m, 2H), 7.54–7.60 (m, 4H), 7.46–7.52 (m, 6H), 7.36–7.42 (m, 2H), 7.15 (s, 1H), 7.09 (s, 1H), 3.76 (t, J = 6.48 Hz, 2H), 2.79 (t, J = 7.34 Hz, 2H) and 2.11 (t, J = 7.09 Hz, 2H). ^13^C NMR (101 MHz, DMSO-d6) δ 183.2, 171.3, 163.4, 153.8, 153.4, 141.8, 139.2, 130.3, 129.4, 129.2, 128.6, 128.5, 126.3, 123.2, 118.9, 107.6, 90.8, 44.9, 31.4, 27.8 and 19.4. HRMS(Q-TOF) m/z calculated for C_32_H_23_ClO_7_ [M + H]^+^: 555.1132 found: 555.1964.

##### 4-(9-Hydroxy-8-oxo-2,2-diphenyl-8H-[1,3]dioxolo[4,5-g]chromen-6-yl)phenyl 4-methylbenzenesulfonate (**10b**)

This compound (0.3 g, yield 75.0%) was prepared from important immediate compound **3** (0.3 g, 0.6 mmol), Et_3_N (0.88 g, 8.6 mmol) and tosyl chloride **9b** (0.19 g, 0.99 mmol) according to the general procedure described above. ^1^H NMR (400 MHz, DMSO-d6) δ 12.90 (s, 1H), 8.06–8.16 (m, 2H), 7.66–7.85 (m, 2H), 7.53–7.62 (m, 4H), 7.41–7.53 (m, 9H), 7.19–7.29 (m, 2H), 7.06 (s, 1H), 7.10 (s, 1H) and 2.42 (s, 3H). ^13^C NMR (101 MHz, DMSO-d6) δ 183.1, 162.7, 153.3, 153.3, 151.9, 146.6, 141.8, 139.2, 131.5, 130.8, 130.3, 130.1, 129.4, 129.2, 128.9, 128.8, 126.3, 123.3, 107.7, 90.7 and 21.7. HRMS(Q-TOF) m/z calculated for C_35_H_24_O_8_S [M + H]^+^: 605.1192 found: 605.1261.

##### 4-(9-Hydroxy-8-oxo-2,2-diphenyl-8H-[1,3]dioxolo[4,5-g]chromen-6-yl)phenyl 2-chloroacetate (**10c**)

This compound (0.37 g, yield 46.3%) was prepared from important immediate compound **3** (0.5 g, 1.1 mmol), Et_3_N (0.28 g, 2.7 mmol) and chloroacetyl chloride **9c** (0.15 g, 1.3 mmol) according to the general procedure described above. With 46.3% yield, ^1^H NMR (400 MHz, DMSO-d6) δ 12.96 (s, 1H), 8.07–8.32 (m, J = 8.80 Hz, 2H), 7.53–7.62 (m, 4H), 7.46–7.53 (m, 7H), 7.40–7.46 (m, J = 8.80 Hz, 2H), 7.14 (s, 1H) and 7.09 (s, 1H), 4.75 (s, 2H). ^13^C NMR (101 MHz, DMSO-d6) δ 183.2, 166.6, 163.3, 153.4, 153.4, 153.2, 141.8, 139.2, 130.3, 129.4, 129.2, 128.6, 126.3, 122.9, 118.9, 107.7, 105.6, 90.7 and 41.9. HRMS(Q-TOF) m/z calculated for C_30_H_19_ClO_7_ [M + H]^+^: 527.0819 found: 527.0889.

##### 4-(9-Hydroxy-8-oxo-2,2-diphenyl-8H-[1,3]dioxolo[4,5-g]chromen-6-yl)phenyl methanesulfonate (**10d**)

This compound (41 mg, yield 70.6%) was prepared from important immediate compound **3** (50 mg, 0.1 mmol), Et_3_N (20 mg, 0.1 mmol) and methanesulfonyl chloride **9d** (10 mg, 0.08 mmol) according to the general procedure described above. ^1^H NMR (400 MHz, DMSO-d6) δ 12.87 (s, 1H), 8.15 (d, J = 8.80 Hz, 2H), 7.47–7.56 (m, 6H), 7.35–7.47 (m, 6H), 7.08 (d, J = 7.34 Hz, 2H) and 3.42 (s, 3H). ^13^C NMR (101 MHz, DMSO-d6) δ ppm 183.18, 162.94, 153.39, 152.04, 141.80, 139.16, 130.25, 130.05, 129.19, 129.16, 129.06, 126.31, 118.98, 107.70, 106.01, 90.77, 38.20 and 19.34. HRMS(Q-TOF) m/z calculated for C_29_H_20_O_8_S [M + H]^+^: 529.0879 found: 529.0951.

##### 4-(9-Hydroxy-8-oxo-2,2-diphenyl-8H-[1,3]dioxolo[4,5-g]chromen-6-yl)phenyl acetate (**10e**)

This compound (0.02 g, yield 40.6%) was prepared from important immediate compound **3** (0.15 g, 0.1 mmol), Et_3_N (0.08 g, 0.7 mmol) and acetyl chloride **9e** (0.07 g, 0.9 mmol) according to the general procedure described above. ^1^H NMR (400 MHz, DMSO-d6) δ 12.93–13.22 (m, 1H), 8.19 (d, J = 7.82 Hz, 2H), 7.59–7.68 (m, 4H), 7.49–7.59 (m, 6H), 7.41 (d, J = 7.83 Hz, 2H), 7.15–7.20 (m, 1H), 7.11 (s, 1H) and 2.36 (s, 3H). ^13^C NMR (101 MHz, DMSO-d6) δ 183.2, 169.4, 163.4, 153.8, 153.3, 141.8, 139.2, 130.2, 129.4, 129.2, 128.5, 128.4, 126.3, 123.2, 107.6, 105.5, 90.7 and 21.4. HRMS(Q-TOF) m/z calculated for C_30_H_20_O_7_ [M + H]^+^: 493.1209 found: 493.1279.

#### 4.2.6. 9-Hydroxy-6-(4-methoxyphenyl)-2,2-diphenyl-8H-[1,3]dioxolo[4,5-g]chromen-8-one (**10f**) and 9-methoxy-6-(4-methoxyphenyl)-2,2-diphenyl-8H-[1,3]dioxolo[4,5-g]chromen-8-one (**11f**)

A mixture of compound **3** (0.2 g, 0.4 mmol), methyl iodide (0.14 g, 0.9 mmol) and excess potassium carbonate (0.25 g, 1.8 mmol) in dry DMF (20 mL) was stirred in ice bath under nitrogen atmosphere for 30 min in the presence of catalytic amount of KI. The solution was allowed to slowly warm to room temperature with stirring overnight. Then, the reaction was diluted with brine (20 mL). The collected organic phase was dried over anhydrous Na_2_SO_4_, filtered and concentrated under reduced pressure. Subsequent purification of the crude mixture was performed using the flash column chromatography on silica gel with gradient elution (EA/PE, 50% to 60%) to afford the target compound **10f** (0.03 g, 50.1% yield) and a side product compound **11f** (0.1 g, 49.8% yield). For **10f**: ^1^H NMR (400 MHz, CDCl_3_) δ 12.79 (s, 1H), 7.65–7.85 (m, 2H), 7.49–7.63 (m, 4H), 7.25–7.43 (m, 6H), 6.86–6.98 (m, 2H), 6.55 (s, 1H), 6.49 (s, 1H) and 3.80 (s, 3H). ^13^C NMR (101 MHz, CDCl_3_) d 183.0, 164.0, 162.6, 153.3, 153.2, 142.4, 139.3, 129.4, 128.4, 128.0, 126.3, 114.5, 107.7, 104.0, 89.4 and 55.5. HRMS(Q-TOF) m/z calculated for C_29_H_20_O_6_ [M + H]^+^: 465.1260 found: 465.1529. For **11f**: ^1^H NMR (400 MHz, CDCl_3_) δ 7.67–7.76 (m, 2H), 7.47–7.56 (m, 4H), 7.24–7.39 (m, 6H), 6.87–6.97 (m, 2H), 6.70 (s, 1H), 6.47 (s, 1H), 4.13 (s, 3H) and 3.79 (s, 3H). ^13^C NMR (101 MHz, CDCl_3_) d 162.1, 160.8, 154.7, 139.2, 129.6, 128.4, 127.6, 126.3, 123.8, 118.6, 114.4, 107.1, 93.3, 61.2 and 55.5. HRMS(Q-TOF) m/z calculated for C_30_H_22_O_6_ [M + H]^+^: 479.1416 found: 479.1498.

#### 4.2.7. 4-(5,6,7-Trihydroxy-4-oxo-4H-chromen-2-yl)phenyl 4-chlorobutanoate (**12a**)

Compound **10a** (0.3 g) was stirred with 10% Pd/C catalyst (0.02 g, 0.1 mmol) in EtOH (10 mL) and THF (10 mL) for 12 h under hydrogen at atmospheric pressure, then the Pd/C catalyst was removed by filtration over celite. After the filtrate was evaporated, the crude material was purified by column chromatography on silica gel using 50% ethyl acetate in petroleum ether as eluent to afford **12a**. With 90% yield, ^1^H NMR (400 MHz, DMSO-d6) δ 8.03–8.29 (m, 2H), 7.29–7.44 (m, 2H), 6.84–7.05 (m, 1H), 6.54–6.74 (m, 1H), 3.76 (t, J = 6.48 Hz, 2H), 2.79 (t, J = 7.21 Hz, 2H) and 2.12 (t, J = 7.09 Hz, 2H). ^13^C NMR (101 MHz, DMSO-d6) δ 182.3, 171.3, 162.4, 153.5, 150.3, 147.5, 129.8, 129.2, 128.3, 128.3, 123.1, 104.7, 44.9, 31.5 and 27.8. HRMS(Q-TOF) m/z calculated for C_19_H_15_ClO_7_ [M − H]^+^: 389.0506 found: 389.6161.

#### 4.2.8. 5-Hydroxy-6,7-dimethoxy-2-(4-methoxyphenyl)-4H-chromen-4-one (**13a**) and 5,6,7-trimethoxy-2-(4-methoxyphenyl)-4H-chromen-4-one (**14a**)

To a mixture solution of compound **2** (0.2 g, 0.7 mmol) and NaH (0.17 g, 7.1 mmol) in anhydrous DMF (15 mL) at 170 ℃ under nitrogen atmosphere commercially available methyl iodide (0.69 g, 4.8 mmol) was added dropwise, stirring well over 12 h. After the disappearance of starting material, as indicated by TLC, the reaction mixture was diluted with EA and water. The collected organic phase was dried over anhydrous Na_2_SO_4_, filtered and concentrated under reduced pressure. Purification of the crude residue was performed by flash column chromatography on silica gel using 50% ethyl acetate in petroleum ether as eluent to afford the desired compound **13a** (0.19 g, 82.9% yield) and a side product **14a** (0.18 g, 80.1% yield). For **13a**: ^1^H NMR (400 MHz, CDCl_3_) δ ppm 3.90 (s, 3 H) 3.93 (s, 3 H), 3.97 (s, 3 H), 6.59 (s, 1 H), 6.55 (s, 1 H), 7.02 (m, J = 9.05 Hz, 2 H), 7.85 (m, J = 9.05 Hz, 2 H) and 12.78 (s, 1 H). ^13^C NMR (101 MHz, CDCl_3_) δ ppm 182.68, 164.02, 162.62, 158.73, 153.23, 153.09, 128.00, 123.58, 114.53, 106.16, 104.15, 90.57, 60.86, 56.32 and 55.55. HRMS(Q-TOF) m/z calculated for 328.32 [M + Na]^+^: 351.3160 found: 351.0843. For **14a**: ^1^H NMR (400 MHz, CDCl_3_) δ ppm 3.87 (s, 3 H), 3.91 (s, 3 H), 3.97 (s, 3 H), 3.98 (s, 3 H), 6.57 (s, 1 H), 6.79 (s, 1 H), 6.95–7.03 (m, 2 H) and 7.75–7.87 (m, 2 H). ^13^C NMR (101 MHz, CDCl_3_) δ ppm 177.18, 162.13, 161.14, 157.61, 154.49, 152.57, 127.63, 123.86, 114.39, 112.87, 107.05, 96.26, 62.19, 61.53, 56.28 and 55.48. HRMS(Q-TOF) m/z calculated for C_19_H_18_O_6_ [M + Na]^+^: 365.3426 found: 365.0996.

#### 4.2.9. 2-(4-Acetoxyphenyl)-5-hydroxy-4-oxo-4H-chromene-6,7-diyl diacetate (**13b**) and 2-(4-acetoxyphenyl)-4-oxo-4H-chromene-5,6,7-triyl triacetate (**14b**)

Compound **2** (0.2 g, 0.6 mmol) was dissolved in pyridine (15 mL) and mixed with acetic anhydride (0.32 g, 3.1 mmol) and a small amount of DMAP as catalyst under nitrogen atmosphere. The reaction mixture was stirred at room temperature overnight. After the disappearance of starting material, as indicated by TLC, the reaction mixture was diluted with DCM and brine. The collected organic phase was dried over anhydrous Na_2_SO_4_, filtered and concentrated under reduced pressure. Purification of the crude residue was performed by flash column chromatography on silica gel using 50% ethyl acetate in petroleum ether as eluent to afford the desired compound **13b** (1.78 g, 56.2% yield) and a side product **14b** (0.26 g, 57.1% yield). For **13b**: ^1^H NMR (400 MHz, CDCl_3_) δ ppm 2.28 (s, 6 H), 2.30 (s, 3 H), 6.63 (s, 1 H), 6.89 (s, 1 H), 7.13–7.31 (m, 3 H), 7.84 (d, J = 8.80 Hz, 2 H) and 12.83 (s, 1 H). ^13^C NMR (101 MHz, CDCl_3_) δ ppm 182.68, 164.02, 162.62, 158.73, 153.23, 153.09, 128.00, 123.58, 114.53, 106.16, 104.15, 90.57, 60.86, 56.32 and 55.55. HRMS(Q-TOF) m/z calculated for C_21_H_16_O_9_ [M + Na]^+^: 435.3463 found: 435.0687. For **14b**: ^1^H NMR (400 MHz, CDCl_3_) δ ppm 2.25–2.30 (m, 10 H), 2.37 (s, 3 H), 6.55 (s, 1 H), 7.19 (t, J = 4.40 Hz, 3 H), 7.42 (s, 1 H) and 7.81 (d, J = 9.05 Hz, 2 H). ^13^CNMR (101 MHz, CDCl_3_) δ ppm 176.08, 168.89, 168.30, 167.25, 166.99, 161.93, 154.18, 153.42, 146.95, 142.19, 132.80, 128.54, 127.63, 122.44, 115.56, 110.27, 108.25, 21.15, 20.82 and 20.09. HRMS(Q-TOF) m/z calculated for C_23_H_18_O_10_ [M + H]^+^: 455.0900 found: 455.0976.

### 4.3. Antiarrhythmic Assay

#### 4.3.1. Antiarrhythmic Effects of Compounds on BaCl_2_-Induced Arrhythmia in Rats

**Figure 11:**
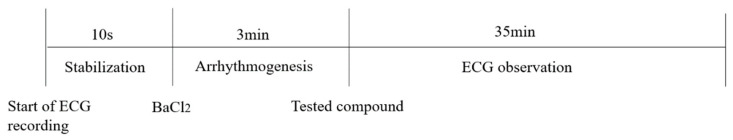
The experimental setup for investigating antiarrhythmic activity of tested compounds in rat models of arrhythmia induced by BaCl_2_.

SD rats (200–220 g) were purchased from Beijing Sibefu Biotechnology Co., LTD (Beijing, China) (SCXK(JING)2019-0010). They were kept in plastic cages at 22 ± 2 °C with free access to pellet food and water on a 12 h light/dark cycle. All of animal experimental procedures were carried out in accordance with the guide for the care and use of laboratory animals (National Research Council of USA, 1996) and related ethical regulations of Yunnan University of Chinese Medicine. As shown in Figure 11, rats were anesthetized with pentobarbital sodium (50 mg/kg), then a normal electrocardiogram was recorded in lead II and barium chloride (4 mg/kg) was injected through sublingual vein. After 3 min of duration of arrhythmia, the rats were randomly divided into negative control groups (NS and DMSO), positive control groups (scutellarein and Ver) and drug administration groups (*n* = 6, respectively). DMSO solution lacking any compounds was used as a negative control as all of tested compounds were dissolved in DMSO. Saline (0.27 mL/kg), DMSO (0.27 mL/kg), Scutellarein (8 mg/kg), Ver (2 mg/kg) and the synthesized compounds (8 mg/kg) were immediately injected into the sublingual vein of each group of rats, respectively. ECG recordings were conducted for 35 min following anesthesia. ECG data recorded for 10 s prior to administration of BaCl_2_ were served as the Normal group. Stable ECG data documented 3 min following administration of BaCl_2_ were acted as the pre-dose group. Stable ECG recovery data posted to administration of investigated compound acted as the post-dose group. The rat number of arrhythmia recovery, corresponding recovery onset time and maintenance time were recorded for antiarrhythmic activity evaluation. The ECG parameters including the HR, RR, QRS and QT intervals were recorded. The QTc interval was also established through using Bazett’s formula: QTc = QT/√RR. All of these aforementioned parameters derived from normal, pre-dose and post-dose groups were compared. In addition, the following parameters including R wave amplitude and T wave amplitude were also measured.

#### 4.3.2. Effects of Compounds on Cardiac Arrhythmia Induced by Aconitine in Rats

**Figure 12:**
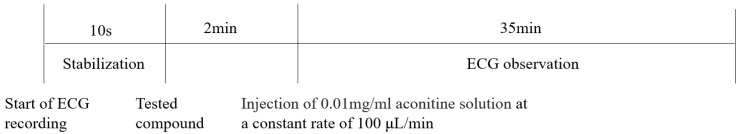
The experimental setup for investigating antiarrhythmic activity of tested compounds in rat models of arrhythmia induced by aconitine.

As shown in Figure 12, rats were anesthetized with pentobarbital sodium (50 mg/kg), then a normal electrocardiogram was recorded in lead II. Saline (0.8 mL/kg), Ver (2 mg/kg) and the synthesized compounds (8 mg/kg) were injected into the sublingual vein. After 2 min, one side of the femoral vein was separated and aconitine (0.01 mg/mL) was injected into the femoral vein at a constant rate of 100 μL/min using an infusion pump to induce ventricular arrhythmia. The time required for onset of VP, VT, VF and CA were recorded and then converted into the cumulative aconitine dosage required to induce VP, VT, VF and CA.

#### 4.3.3. The Effect of Investigated Compounds on Normal Electrocardiogram in Rats

**Figure 13:**
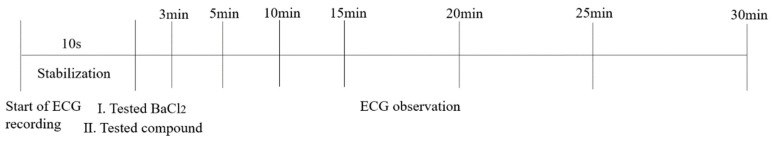
The experimental setup for investigating proarrhythmic effect of tested compounds in normal rat.

As shown in Figure 13, in vivo electrocardiographic investigations were carried out using an BL-420 multiple physiological signal acquisition and analysis system with a standard lead II mode. The tested compounds were administered through sublingual vein at a dose of 8 mg/kg after 3 min following general anesthesia with pentobarbital sodium (50 mg/kg), while the BaCl_2_ as the positive control was given into sublingual vein at a dose of 4 mg/kg, so as to NS and DMSO as the negative control. The ECG was recorded just 3, 5, 10, 15, 20, 25 and 30 min following general anesthesia, since the rat would be awake after 35 min following general anesthesia.

### 4.4. Cytotoxicity Assay

The cell proliferation assay was based on the reduction of (2-(2-Methoxy-4-nitrophenyl)-3-(4-nitrophenyl)-5-(2,4-disulfophenyl)-2H-tetrazolium Sodium Salt (WST-8) to yellow formazan dye by succinate dehydrogenase in living cells. Cells in 100 μL of culture media were seeded into 96-well plate at a density of 5000 cells per well and incubated for 4 h at 37 °C. Then, the cells were exposed to various concentrations of compound **10e** in DMSO for 48 h before CCK-8 assay. Medium containing 0.5% DMSO was used as a negative control. Medium without cells was used as blank control. After treatment, CCK-8 reagent (HB-CCK8-1, Hanbio Biotechnology, China) in PBS was added to each well and the cells were further incubated for 1 h at 37 °C according to the manufacturer’s instructions. The optical density was measured at 450 nm using a Microplate Reader (Clariostar, BMG, Ortenberg, Germany). The percentage of surviving cells was calculated using the following formula: (corrected reading from the test well/corrected reading from the blank well)/(corrected reading from negative well-corrected reading from the blank well) × 100%.

### 4.5. Patch Clamp Assay for Nav1.5, Cav1.2 and hERK

#### 4.5.1. Cell Culture

Human embryonic kidney cells (HEK293) that showed no macroscopic sodium currents were used for patch clamp assay and purchased from the Shanghai Institute of Biological Sciences, Chinese Academy of Sciences (Shanghai, China). HEK293 cells were cultured in medium consisting of 90% DMEM, 10% fetal bovine serum, 1% penicillin and streptomycin at 37 °C and 5% CO_2_ atmosphere.

#### 4.5.2. Cell Transfection

The Nav1.5 (SCN5A) plasmid was purchased from Addgene and the GFP plasmid was purchased from Shenzhen Yanming Biotechnology. According to the manufacturer’s instructions, HEK293 cells were transfected with GFP plasmids using pCDNA3.1hSCN5A for 6–8 h. Subsequently, patch clamp assays were carried out in whole-cell mode after about 24 h transfection.

The Cav1.2 (CACNA2D) plasmid was purchased from Youbio and the GFP plasmid was purchased from Beijing Bozhiyuan Biotechnology Co., Ltd. (Beijing, China). According to the manufacturer’s instructions, HEK293 cells were transfected with GFP plasmids using pCDNA3.1hCACNA2D for 6–8 h. Subsequently, patch clamp assays were carried out in whole-cell mode after about 24 h transfection.

The HEK293 cell lines were transfected with pcmv-kcnh2-egfp-neo plasmid purchased from Shenzhen Dingke Biotechnology Co., Ltd. (Shenzhen, China) using jetPRIME (polyplus, 101000046) when cell confluence reached 60–80%. Electrophysiological experiments were performed 24–48 h after cell transfection.

#### 4.5.3. Patch Clamp Measurements for Nav1.5

Patch clamp experiments were conducted by using an EPC-10.2 patch-clamp amplifier (HEKA Elektronik, Reutlingen, Germany) and patchmaster v2x91 acquisition software in transfected HEK293 cells with heterologous expression of human Nav1.5 channels. Before membrane Nav1.5 currents recorded, cells were replated on glass coverslips and mounted on a recording chamber. The extracellular recording solution for recording sodium current consisted of (in mM) 140 NaCl, 30 tetraethylammonium chloride, 10 D-glucose, 3 KCl, 1 CaCl_2_, 0.5 CdCl_2_, 1 MgCl_2_ and 10 HEPES. The pH of the solution was adjusted to 7.3 by using NaOH, meanwhile the osmolality was maintained at 310–315 mOsm/L. The composition of the intracellular recording solution was (in mM) 140 CsF, 10 NaCl, 15 HEPES and 1.1 Cs-EGTA, in which pH was adjusted to 7.3 with CsOH. HEK293 cells were used for all records. The current voltage (I-V) measurement was recorded by 50 ms square pulses ranging from −100 to +60 mV, with 5 mV increment from a holding potential of −120 mV. The corresponding current density was obtained. Voltage dependency for sodium current activation was recorded by 50 ms square pulses ranging from −100 to +40 mV, with 5 mV increment from a holding potential of −120 mV. Data were fitted with the modified Boltzman function $G=\frac{G_{max}}{1+e^{\left(\frac{m-m_{a}}{ka}\right)}}$, where V_1/2_ was the voltage in which half of the current is activated. Em was the membrane potential and K was the slope factor. Voltage-dependent inactivation was measured in a 50 ms duration test pulse to −30 mV amplitude immediately after 5000 ms conditioning pulse ranging from −140 mV to −35 mV, with 5 mV increment from a holding potential of −120 mV. Peak currents from the test pulse were normalized by maximum current (I/I_MAX_) and also fitted with the modified Boltzman function $\frac{I}{Imax}=\frac{1}{1+e^{\left(\frac{Em-\mathrm{Vh}}{kh}\right)}}$ to calculate the potential Vh related to the half of inactivation and slope factor kh.

#### 4.5.4. Patch Clamp Measurements for Cav1.2

The extracellular recording solution for recording calcium current consisted of (in mM) 140 TEA-Cl, 1 MgCl_2_•6H_2_O, 4 KCl, 10 CaCl_2_•2H_2_O, 10 D-Glucose and 5 mM HEPES. The pH of the solution was adjusted to 7.4 using TEA-OH. The composition of the intracellular recording solution was (in mM) 110 CsCl, 10 EGTA, 5 HEPES and 5 Mg-ATP, in which pH was adjusted to 7.2 with CsOH. Cav1.2 cells were used for all records. The stimulation procedure is shown below. The clamping voltage was set to −80 mV, then depolarized to 10 mV over a time range of 400 ms, then repolarized to −80 mV. The current was recorded every 20 s. The original data Cav1.2 current peak was extracted from the PatchMaster v2x91 software, and the current inhibition rate was calculated as follows: peak calcium current inhibition rate = 1 − (Peak current compound/Peak current vehicle). The mean and standard error were calculated for each concentration and the concentration–effect relationship was obtained. The analysis statistics were completed using Graphpad Prism 5.0 software.

#### 4.5.5. Patch Clamp Measurements for hERG

The extracellular recording solution for recording calcium current consisted of (in mM) 140 NaCl, 3.5 KCl, 1 MgCl_2_•6H_2_O, 2 CaCl_2_•2H_2_O, 10 D-Glucose, 10 HEPES and 1.25 NaH_2_PO_4_•2H_2_O. The pH of the solution was adjusted to 7.4 using NaOH. The composition of the intracellular recording solution was (in mM) 10 NaCl, 50 CsCl, 60 CsF, 10 HEPES and 20 EGTA, in which pH was adjusted to 7.2 with CsOH. The current was recorded by depolarizing the voltage to +40 mV for 2.5 s from the holding potential at −80 mV, followed by a hyperpolarization step to −50 mV lasting for 4 s, which elicited the peak tail current of hERG channel. The analysis statistics were completed by using Graphpad Prism 5.0 software.

### 4.6. Computational Docking Studies

Autodock vina software was used for docking study. The 3D structure of compound **10e** were generated from ChemDraw 22.0 software and imported into the Autodock vina software. The X-ray crystal structure of the human-derived protein Nav1.5 (PDB ID:6LQA) and Cav1.2 (PDB ID 8FD7) obtained from the Protein Data Bank were used for docking after removal of the water molecules and ions or small molecule compounds bound to the surface of protein. The position of the self-matched antibody single-chain variable fragment in the crystal was identified as the substrate-binding site of Nav1.5 or Cav1.2 protein. Compound **10e** performed energy optimization before docking. The potential binding result positioned compound **10e** into the substrate-binding site of Nav1.5 and Cav1.2 with hydrogen bonding interactions, respectively.

### 4.7. Statistical Analysis

Results were presented as either means ± SD. In our analysis, statistical analyses were performed using single-factor ANOVA test or non-parametric test followed by Welch test or Kruskal–Wallis test.

## Figures and Tables

**Table 1:** Onset time and duration of efficacy of scutellarein derivatives on BaCl_2_-induced arrhythmia in rats.

Group	Onset Time(s)	Duration of Efficacy(s)	Number of Rats	
			Duration of Efficacy ≤ 10 min	Duration of Efficacy ≥ 20 min
NS	>2100.00	0.00	0	0
DMSO	>2100.00	0.00	0	0
Ver (2 mg/kg)	17.00 ± 17.53 ***+++▲▲▲	1261.00 ± 631.59 ***+++	1	4
Scutellarein (8 mg/kg)	585.83 ± 393.07 *+##	1100.00 ± 462.37 ***+++	0	2
**3** (8 mg/kg)	9.16 ± 9.37 ***+++▲▲▲	585.66 ± 520.91 *+	3	1
**7a** (8 mg/kg)	11.50 ± 6.92 ***+++▲▲▲	889.50 ± 730.54 **++	3	3
**7b** (8 mg/kg)	12.50 ± 16.28 ***+++▲▲▲	1061.33 ± 524.28 ***+++	2	2
**7c** (8 mg/kg)	21.26 ± 18.55 ***+++▲▲▲	338.00 ± 359.30 *+	5	0
**7d** (8 mg/kg)	237.83 ± 286.13 ***+++▲▲▲	394.33 ± 399.63 *+	4	0
**7e** (8 mg/kg)	16.50 ± 5.70 ***+++▲▲▲	554.66 ± 354.68 **++	3	0
**7f** (8 mg/kg)	13.83 ± 12.49 ***+++▲▲▲	558.00 ± 477.65 **++	3	1
**7g** (8 mg/kg)	7.16 ± 8.98 ***+++▲▲▲	780.83 ± 254.22 **++	1	1
**8a** (8 mg/kg)	18.66 ± 10.22 ***+++▲▲▲	530.66 ± 526.69 *+	3	1
**10a** (8 mg/kg)	43.00 ± 19.47 ***+++▲▲▲	1303.50 ± 491.97 ***+++	0	4
**10b** (8 mg/kg)	48.00 ± 33.68 ***+++▲▲▲	686.50 ± 719.42 **++	4	2
**10c** (8 mg/kg)	35.50 ± 21.73 ***+++▲▲▲	368.83 ± 673.02	5	1
**10d** (8 mg/kg)	19.83 ± 15.07 ***+++▲▲▲	770.33 ± 575.33 **++	3	1
**10e** (8 mg/kg)	25.00 ± 16.46 ***+++▲▲▲	1677.33 ± 357.66 ***+++▲	1	5
**10f** (8 mg/kg)	76.83 ± 57.62 ***+++▲▲▲	337.16 ± 435.75	5	1
**11f** (8 mg/kg)	107.00 ± 131.10 ***+++▲▲▲	1336.00 ± 230.05 ***+++	0	5
**12a** (8 mg/kg)	42.00 ± 73.35 ***+++▲▲▲	896.66 ± 681.59 **++	3	3
**13a** (8 mg/kg)	10.33 ± 9.21 ***+++▲▲▲	25.66 ± 18.31	6	0
**13b** (8 mg/kg)	71.33 ± 76.50 ***+++▲▲▲	1063.83 ± 645.22 ***+++	2	3
**14a** (8 mg/kg)	39.00 ± 49.13 ***+++▲▲▲	20.00 ± 11.64	6	0
**14b** (8 mg/kg)	59.83 ± 38.55 ***+++▲▲▲	229.33 ± 149.75 *+	6	0

Compared with NS: * *p* < 0.05 ** *p *< 0.01 *** *p *< 0.001; Compared with DMSO: + *p *< 0.05 ++ *p *< 0.01 +++ *p *< 0.001; Compared with Ver: ## *p *< 0.01; Compared with scutellarein: ▲ *p *< 0.05 ▲▲▲ *p *< 0.001; Data represent mean ± SD. *n* = 6.

**Table 2:** Influence of the synthetic compounds on ECG intervals QT. Normal group referring to anesthesia group without any treatments, pre-dose group referring to BaCl_2_ treated group, post-dose group referring to compounds-treated group.

Group	QT (ms)		
	Normal	Pre-Dose	Post-Dose
NS	38.78 ± 2.03	43.51 ± 3.61 ***	45.18 ± 2.54 ***
DMSO	40.65 ± 0.84	44.89 ± 1.78 ***	46.93 ± 2.53 ***
Ver (2 mg/kg)	39.72 ± 1.36	43.51 ± 3.61 ***	45.18 ± 2.54 ***
Scutellarein (8 mg/kg)	42.48 ± 2.10	45.16 ± 1.62 *	41.14 ± 3.19 ###▲▲▲
**3** (8 mg/kg)	37.88 ± 1.14	43.21 ± 2.05 *	38.39 ± 2.74 ###▲
**7a** (8 mg/kg)	39.75 ± 1.44	47.45 ± 3.13 *	41.49 ± 2.60 #▲
**7b** (8 mg/kg)	38.08 ± 1.97	44.37 ± 2.99 *	38.49 ± 3.36 ###
**7c** (8 mg/kg)	39.96 ± 2.84	45.68 ± 3.38 *	40.56 ± 3.11 ##
**7d** (8 mg/kg)	38.54 ± 1.09	43.46 ± 4.43	39.82 ± 3.76 ###
**7e** (8 mg/kg)	37.33 ± 2.89	43.38 ± 3.68 *	37.55 ± 4.31 ###
**7f** (8 mg/kg)	40.87 ± 4.46	45.87 ± 3.23 *	41.76 ± 1.93 ##▲
**7g** (8 mg/kg)	39.08 ± 2.58	44.89 ± 4.16 *	39.77 ± 2.17 ###▲
**8a** (8 mg/kg)	37.85 ± 1.14	41.79 ± 2.93	39.44 ± 3.31 ###
**10a** (8 mg/kg)	40.01 ± 3.45	44.55 ± 3.26 *	40.90 ± 5.43 ##
**10b** (8 mg/kg)	39.47 ± 2.19	45.53 ± 2.79	42.77 ± 3.52 *#
**10c** (8 mg/kg)	38.78 ± 2.00	44.53 ± 2.67 *	39.37 ± 4.12 ###
**10d** (8 mg/kg)	39.72 ± 1.39	44.79 ± 3.73	41.05 ± 2.64 ##
**10e** (8 mg/kg)	37.50 ± 2.06	42.52 ± 2.02	40.66 ± 2.17 ##▲
**10f** (8 mg/kg)	36.54 ± 2.29	43.31 ± 3.74 *	39.67 ± 1.49 ###▲▲
**11f** (8 mg/kg)	37.62 ± 1.10	44.68 ± 2.92 *	41.74 ± 1.95 *##▲
**12a** (8 mg/kg)	38.29 ± 0.77	41.39 ± 1.45 *	41.93 ± 2.98 *#
**13a** (8 mg/kg)	40.95 ± 3.02	46.46 ± 3.85	39.28 ± 6.57 ###
**13b** (8 mg/kg)	39.77 ± 0.88	42.85 ± 5.19 *	39.77 ± 3.89
**14a** (8 mg/kg)	41.37 ± 2.93	47.40 ± 1.91	43.46 ± 3.19 ###
**14b** (8 mg/kg)	40.36 ± 1.68	44.71 ± 2.32 *	40.26 ± 3.44 ##▲▲

Compared with Normal: * *p *< 0.05 *** *p *< 0.001; Compared with NS: # *p *< 0.05 ## *p *< 0.01 ### *p *< 0.001; Compared with DMSO: ▲ *p *< 0.05 ▲▲ *p *< 0.01 ▲▲▲ *p *< 0.001; Data represent mean ± SD. *n* = 6.

**Table 3:** Influence of the synthetic compounds on ECG intervals QTc. Normal group referring to anesthesia group without any treatments, pre-dose group referring to BaCl_2_-treated group and post-dose group referring to compounds-treated group.

Group	QTc (ms)		
	Normal	Pre-Dose	Post-Dose
NS	0.10 ± 0.01	0.12 ± 0.02 **	0.12 ± 0.01 *
DMSO	0.11 ± 0.002	0.11 ± 0.01 **	0.12 ± 0.02
Ver (2 mg/kg)	0.10 ± 0.01	0.12 ± 0.02 **	0.12 ± 0.01 *
Scutellarein (8 mg/kg)	0.11 ± 0.01	0.12 ± 0.02 *	0.10 ± 0.01 ##▲▲
**3** (8 mg/kg)	0.11 ± 0.01	0.12 ± 0.01 *	0.10 ± 0.01 #▲
**7a** (8 mg/kg)	0.10 ± 0.01	0.12 ± 0.02 *	0.10 ± 0.02 #▲
**7b** (8 mg/kg)	0.11 ± 0.01	0.13 ± 0.01 **	0.10 ± 0.01 #▲
**7c** (8 mg/kg)	0.11 ± 0.01	0.13 ± 0.01 **	0.11 ± 0.01
**7d** (8 mg/kg)	0.09 ± 0.004	0.12 ± 0.01 ***	0.09 ± 0.01 ###▲▲▲
**7e** (8 mg/kg)	0.10 ± 0.01	0.12 ± 0.01 **	0.09 ± 0.01 ##▲▲
**7f** (8 mg/kg)	0.11 ± 0.01	0.14 ± 0.01 **	0.11 ± 0.02
**7g** (8 mg/kg)	0.10 ± 0.01	0.13 ± 0.01 ***	0.10 ± 0.01 ##▲▲
**8a** (8 mg/kg)	0.09 ± 0.01	0.12 ± 0.01 ***	0.10 ± 0.01 ##▲
**10a** (8 mg/kg)	0.11 ± 0.01	0.13 ± 0.01 ***	0.11 ± 0.01 #▲
**10b** (8 mg/kg)	0.10 ± 0.01	0.12 ± 0.01 *	0.11 ± 0.01
**10c** (8 mg/kg)	0.10 ± 0.01	0.13 ± 0.01 **	0.10 ± 0.02
**10d** (8 mg/kg)	0.11 ± 0.01	0.12 ± 0.02 **	0.11 ± 0.01
**10e** (8 mg/kg)	0.10 ± 0.01	0.11 ± 0.01	0.10 ± 0.01 ##▲
**10f** (8 mg/kg)	0.09 ± 0.003	0.10 ± 0.02	0.10 ± 0.01 ##▲▲
**11f** (8 mg/kg)	0.09 ± 0.01	0.10 ± 0.03 *	0.10 ± 0.01 ##▲▲
**12a** (8 mg/kg)	0.10 ± 0.003	0.11 ± 0.01 *	0.10 ± 0.01 ##▲▲
**13a** (8 mg/kg)	0.11 ± 0.01	0.14 ± 0.01 ***	0.09 ± 0.01 *###▲▲▲
**13b** (8 mg/kg)	0.11 ± 0.002	0.10 ± 0.01 *	0.10 ± 0.01 #▲
**14a** (8 mg/kg)	0.10 ± 0.003	0.13 ± 0.01 **	0.09 ± 0.01 *###▲▲▲
**14b** (8 mg/kg)	0.10 ± 0.004	0.10 ± 0.01	0.09 ± 0.01 ###▲▲▲

Compared with Normal: * *p *< 0.05 ** *p *< 0.01 *** *p *< 0.001; Compared with NS: # *p *< 0.05 ## *p *< 0.01 ### *p *< 0.001; Compared with DMSO: ▲ *p *< 0.05 ▲▲ *p *< 0.01 ▲▲▲ *p *< 0.001; Data represent mean ± SD. *n* = 6.

**Table 4:** Influence of the synthetic compounds on ECG amplitude P wave. Normal group referring to anesthesia group without any treatments, pre-dose group referring to BaCl_2_-treated group and post-dose group referring to compounds-treated group.

Group	P Wave (mV)		
	Normal	Pre-Dose	Post-Dose
NS	0.096 ± 0.026	0.060 ± 0.012 **	0.150 ± 0.041
DMSO	0.105 ± 0.012	0.071 ± 0.003 **	0.100 ± 0.020 *
Ve r(2 mg/kg)	0.116 ± 0.011	0.076 ± 0.027 **	0.228 ± 0.027 ▲
Scutellarein (8 mg/kg)	0.116 ± 0.017	0.073 ± 0.013 ***	0.188 ± 0.040
**3** (8 mg/kg)	0.128 ± 0.015	0.053 ± 0.021 ***	0.215 ± 0.044 #▲▲
**7a** (8 mg/kg)	0.121 ± 0.017	0.063 ± 0.015 ***	0.171 ± 0.052 *
**7b** (8 mg/kg)	0.136 ± 0.013	0.098 ± 0.032 **	0.200 ± 0.042 *
**7c** (8 mg/kg)	0.091 ± 0.033	0.070 ± 0.015	0.145 ± 0.038
**7d** (8 mg/kg)	0.146 ± 0.019	0.088 ± 0.033 ***	0.183 ± 0.051 ##▲▲▲
**7e** (8 mg/kg)	0.118 ± 0.010	0.065 ± 0.009 ***	0.156 ± 0.033
**7f** (8 mg/kg)	0.178 ± 0.024	0.108 ± 0.006 ***	0.133 ± 0.020 **###▲▲▲
**7g** (8 mg/kg)	0.163 ± 0.037	0.086 ± 0.011 ***	0.121 ± 0.022 ###▲▲▲
**8a** (8 mg/kg)	0.110 ± 0.015	0.083 ± 0.014 *	0.170 ± 0.053
**10a** (8 mg/kg)	0.128 ± 0.017	0.096 ± 0.015 **	0.188 ± 0.042 #▲
**10b** (8 mg/kg)	0.115 ± 0.026	0.071 ± 0.022 **	0.153 ± 0.054 ▲
**10c** (8 mg/kg)	0.110 ± 0.027	0.073 ± 0.024 ***	0.186 ± 0.045
**10d** (8 mg/kg)	0.111 ± 0.013	0.048 ± 0.010 ***	0.170 ± 0.061
**10e** (8 mg/kg)	0.130 ± 0.025	0.066 ± 0.024 ***	0.135 ± 0.016
**10f** (8 mg/kg)	0.116 ± 0.025	0.045 ± 0.013 ***	0.138 ± 0.059 #▲▲
**11f** (8 mg/kg)	0.115 ± 0.012	0.083 ± 0.017 *	0.156 ± 0.038 ##▲▲▲
**12a** (8 mg/kg)	0.118 ± 0.016	0.061 ± 0.006 ***	0.136 ± 0.051 **
**13a** (8 mg/kg)	0.090 ± 0.012	0.025 ± 0.007 ***	0.123 ± 0.015
**13b** (8 mg/kg)	0.118 ± 0.012	0.071 ± 0.010 ***	0.161 ± 0.017
**14a** (8 mg/kg)	0.098 ± 0.017	0.060 ± 0.014 **	0.111 ± 0.008
**14b** (8 mg/kg)	0.111 ± 0.006	0.083 ± 0.012 *	0.128 ± 0.008

Compared with Normal: * *p *< 0.05 ** *p *< 0.01 *** *p *< 0.001; Compared with NS: # *p *< 0.05 ## *p *< 0.01 ### *p *< 0.001; Compared with DMSO: ▲ *p *< 0.05 ▲▲ *p *< 0.01 ▲▲▲ *p *< 0.001; Data represent mean ± SD. *n* = 6.

**Table 5:** Influence of the synthetic compounds on ECG amplitude T wave. Normal group referring to anesthesia group without any treatments, pre-dose group referring to BaCl_2_-treated group and post-dose group referring to compounds-treated group.

Group	T Wave (mV)		
	Normal	Pre-Dose	Post-Dose
NS	0.180 ± 0.035	0.108 ± 0.025 ***	0.150 ± 0.041
DMSO	0.121 ± 0.052	0.095 ± 0.022	0.100 ± 0.020
Ver (2 mg/kg)	0.211 ± 0.033	0.141 ± 0.049 ***	0.228 ± 0.027 ##▲▲▲
Scutellarein (8 mg/kg)	0.198 ± 0.026	0.118 ± 0.021 ***	0.188 ± 0.040 ▲▲▲
**3** (8 mg/kg)	0.215 ± 0.022	0.103 ± 0.021 ***	0.215 ± 0.044 ##▲▲▲
**7a** (8 mg/kg)	0.203 ± 0.035	0.151 ± 0.040 **	0.171 ± 0.052 *▲
**7b** (8 mg/kg)	0.210 ± 0.011	0.178 ± 0.038	0.200 ± 0.042 #▲▲▲
**7c** (8 mg/kg)	0.163 ± 0.058	0.138 ± 0.052	0.145 ± 0.038 ▲
**7d** (8 mg/kg)	0.158 ± 0.034	0.125 ± 0.041 *	0.183 ± 0.051 ▲▲▲
**7e** (8 mg/kg)	0.200 ± 0.023	0.118 ± 0.015 ***	0.156 ± 0.033 ▲
**7f** (8 mg/kg)	0.143 ± 0.033	0.113 ± 0.017	0.133 ± 0.020
**7g** (8 mg/kg)	0.111 ± 0.031	0.066 ± 0.022 **	0.121 ± 0.022
**8a** (8 mg/kg)	0.186 ± 0.040	0.155 ± 0.057 ***	0.170 ± 0.053 **▲▲
**10a** (8 mg/kg)	0.193 ± 0.023	0.156 ± 0.030	0.188 ± 0.042 #▲▲▲
**10b** (8 mg/kg)	0.200 ± 0.048	0.103 ± 0.033 **	0.153 ± 0.054
**10c** (8 mg/kg)	0.183 ± 0.031	0.105 ± 0.031 ***	0.186 ± 0.045 ▲▲▲
**10d** (8 mg/kg)	0.190 ± 0.042	0.075 ± 0.013 ***	0.170 ± 0.061 ▲▲
**10e** (8 mg/kg)	0.183 ± 0.049	0.100 ± 0.040 ***	0.135 ± 0.016
**10f** (8 mg/kg)	0.158 ± 0.032	0.071 ± 0.017 ***	0.138 ± 0.059
**11f** (8 mg/kg)	0.146 ± 0.015	0.105 ± 0.029	0.156 ± 0.038 ▲
**12a** (8 mg/kg)	0.200 ± 0.016	0.085 ± 0.018 ***	0.136 ± 0.051 ***
**13a** (8 mg/kg)	0.118 ± 0.014	0.073 ± 0.017 *	0.123 ± 0.015
**13b** (8 mg/kg)	0.155 ± 0.009	0.081 ± 0.019 ***	0.161 ± 0.017 ▲
**14a** (8 mg/kg)	0.138 ± 0.024	0.080 ± 0.023 **	0.111 ± 0.008
**14b** (8 mg/kg)	0.165 ± 0.017	0.101 ± 0.013 **	0.128 ± 0.008

Compared with Normal: * *p *< 0.05 ** *p *< 0.01 *** *p *< 0.001; Compared with NS: # *p *< 0.05 ## *p *< 0.01; Compared with DMSO: ▲ *p *< 0.05 ▲▲ *p *< 0.01 ▲▲▲ *p *< 0.001; Data represent mean ± SD. *n* = 6.

**Table 6:** Onset time and duration of efficacy of compounds on arrhythmia induced by BaCl_2_ in rats.

Group	Onset Time(s)	Duration of Efficacy(s)	Number of Rats	
			Duration of Efficacy ≤ 10 min	Duration of Efficacy ≥ 20 min
NS	>2100.00	0.00	0	0
DMSO	>2100.00	0.00	0	0
Ver (2 mg/kg)	17.00 ± 17.53	1261.00 ± 631.59	1	4
Scutellarein (8 mg/kg)	585.83 ± 393.07	1100.00 ± 462.37	0	2
**10e** (2 mg/kg)	47.00 ± 13.31 *++▲▲▲	589.83 ± 68.81 **++	3	0
**10e** (4 mg/kg)	33.50 ± 13.04 **+++▲▲▲	987.00 ± 65.10 ***+++	0	0
**10e** (8 mg/kg)	25.00 ± 16.46 **++▲▲▲	1677.33 ± 357.66 ***+++	1	5
**10e** (16 mg/kg)	34.66 ± 21.87 **++▲▲▲	1092.33 ± 93.73 ***+++	0	1

Compared with NS: * *p *< 0.05 ** *p *< 0.01 *** *p *< 0.001; Compared with DMSO: ++ *p *< 0.01 +++ *p *< 0.001; Compared with scutellarein: ▲▲▲ *p *< 0.001; Data represent mean ± SD. *n* = 6.

**Table 7:** Effects of compounds on cardiac arrhythmia induced by aconitine in rats. VP: Ventricular premature, VT: ventricular tachycardia, VF: ventricular fibrillation, CA: cardiac arrest.

Group	Dosage of Aconitine (μg)			
	VP	VT	VF	CA
NS	3.78 ± 3.22	6.15 ± 3.67	8.54 ± 3.44	11.90 ± 5.00
DMSO	1.48 ± 1.48	1.83 ± 1.83	6.43 ± 6.43	8.83 ± 8.83
Ver (2 mg/kg)	1.84 ± 1.27	4.74 ± 3.04	9.39 ± 3.01	11.65 ± 3.53
Scutellarin (12 mg/kg)	7.22 ± 5.76	9.09 ± 6.28	18.34 ± 7.08	21.78 ± 5.85
Scutellarin (8 mg/kg)	4.89 ± 5.87	6.44 ± 5.80	18.01 ± 6.34	19.08 ± 5.61
Scutellarein (12 mg/kg)	8.25 ± 5.60	13.53 ± 5.06	31.11 ± 9.43	36.88 ± 10.61
Scutellarein (8 mg/kg)	1.13 ± 0.27	4.77 ± 0.73	7.46 ± 1.06	10.38 ± 1.02
**10a** (8 mg/kg)	3.59 ± 1.52 +	6.35 ± 3.23 +	8.79 ± 3.64	31.23 ± 18.67 *+
**10e** (2 mg/kg)	0.54 ± 0.08	3.77 ± 1.02	5.45 ± 1.23	8.68 ± 1.28
**10e** (4 mg/kg)	0.91 ± 0.48	3.67 ± 0.29	5.81 ± 0.88	8.01 ± 1.49
**10e** (8 mg/kg)	1.64 ± 0.69	3.39 ± 0.76	22.47 ± 6.00 ***+++##▲	77.24 ± 26.84 ***+++###▲▲
**10e** (16 mg/kg)	1.35 ± 0.25	4.03 ± 0.29	6.50 ± 1.49	9.36 ± 0.47
**11f** (8 mg/kg)	1.69 ± 0.24	3.52 ± 0.57	7.06 ± 3.82	50.84 ± 14.42 **++##

Compared with NS: * *p *< 0.05 ** *p *< 0.01 *** *p *< 0.001; Compared with DMSO: + *p *< 0.05 ++ *p *< 0.01 +++ *p *< 0.001; Compared with Ver: ## *p *< 0.01 ### *p *< 0.001; Compared with scutellarein (8 mg/kg): ▲ *p *< 0.05 ▲▲ *p *< 0.01; Data represent mean ± SD. *n* = 6.

## Data Availability

The data will be provided upon request.

## Supplementary Materials

Supporting information including NMR spectrum, ECG and morphological changes can be downloaded at: https://www.mdpi.com/article/10.3390/molecules28217417/s1.
