# Supplementary material for: Synthesis and In Vivo Antiarrhythmic Activity Evaluation of Novel Scutellarein Analogues as Voltage-Gated Nav1.5 and Cav1.2 Channels Blockers
Source: Molecules. 2023 Nov 3;28(21):7417. doi: 10.3390/molecules28217417 (PMC10650756; doi:10.3390/molecules28217417)

Supporting Information

Table of Contents

S1. Normal rat ECG and arrhythmia ECG

S2. HR of normal group, Pre-dose group and Post-dose group

S3. RR of normal group, Pre-dose group and Post-dose group

S4. QRS of normal group, Pre-dose group and Post-dose group

S5. Investigated compounds induced alterations of HR(bpm), QT(ms), QTc-Bazett's and RR (ms) in anesthetized rats

S6. Patch clamp assay for Cav1.2

S7. Patch clamp assay for Nav1.5

S8. Morphological changes in H9c2 cells following administration of 10e.

S9. 1H NMR and 13C NMR spectrum of target compounds

S1. Normal rat ECG (A), barium chloride-induced arrhythmia ECG (B), Aconitine-induced VP ECG (C), Aconitine-induced VT ECG (D), Aconitine-induced VF ECG (E).

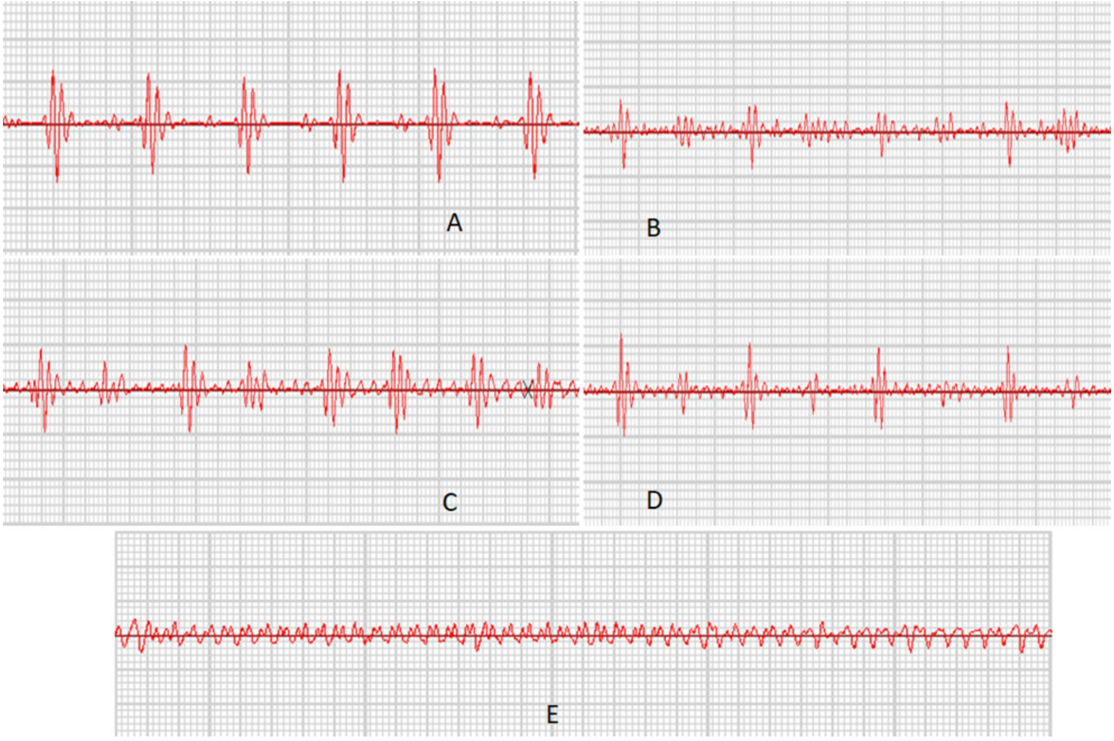

S2. HR of normal group, Pre-dose group and Post-dose group

Group	HR（bpm)		
	Normal	Pre-dose	Post-dose
NS	457.69±29.37	496.96±89.63**	455.07±65.56
DMSO	480.76±19.23	487.08±123.15	416.62±114.72
Ver(2mg/kg)	457.69±29.37	496.96±89.63	455.07±65.56
Scutellarein(8mg/kg)	438.16±58.49	417.74±130.33*	447.61±69.33
**3**(8mg/kg)	447.11±32.25	473.02±88.66	395.97±54.10
**7a**(8mg/kg)	434.79±21.43	415.40±136.69	408.85±121.57
**7b**(8mg/kg)	506.06±51.60	540.90±109.56**	458.00±85.39
**7c**(8mg/kg)	487.17±18.13	551.16±54.52*	551.16±54.52##▲▲▲
**7d**(8mg/kg)	388.58±24.14	535.06±51.62***	336.95±78.60#
**7e**(8mg/kg)	474.35±18.13	504.76±111.77*	404.18±107.98*#
**7f**(8mg/kg)	439.56±15.54	590.90±20.32**	425.82±113.45
**7g**(8mg/kg)	459.60±36.01	563.63±25.71**	403.45±92.94*#
**8a**(8mg/kg)	411.83±51.77	550.00±76.37**	427.51±42.19
**10a**(8mg/kg)	439.81±64.75	531.106±106.50***	426.60±105.96
**10b**(8mg/kg)	473.92±52.55	558.74±49.84**	463.50±84.38
**10c**(8mg/kg)	468.42±55.21	503.03±124.94**	532.98±43.10#▲▲
**10d**(8mg/kg)	479.33±73.05	470.34±127.04	467.09±96.41
**10e**(8mg/kg)	481.68±27.59	456.47±119.39*	401.48±96.42*
**10f**(8mg/kg)	436.85±35.64	340.19±94.56**	400.79±70.65
**11f**(8mg/kg)	402.25±62.74	376.02±176.95*	348.81±55.95##
**12a**(8mg/kg)	487.17±18.13	486.36±117.33	398.60±94.84*
**13a**(8mg/kg)	456.08±50.88	562.50±83.85**	392.74±126.73
**13b**(8mg/kg)	434.09±47.65	287.26±21.40***	379.61±65.55
**14a**(8mg/kg)	457.87±31.98	558.74±49.84**	362.13±109.21*#
**14b**(8mg/kg)	439.42±50.14	325.49±58.85**	339.85±60.55*##

Compared with Normal： *P＜0.05 **P＜0.01 ***P＜0.001

Compared with NS： #P < 0.05 ##P < 0.01 ###P < 0.001

Compared with DMSO： ▲P < 0.05 ▲▲P < 0.01 ▲▲▲P < 0.001

Data represent mean ± SD. n = 6

S3. RR of normal group, Pre-dose group and Post-dose group

Group	RR（s）		
	Normal	Pre-dose	Post-dose
NS	0.132±0.009	0.126±0.033	0.135±0.022
DMSO	0.125±0.005	0.133±0.040**	0.158±0.052
Ver(2mg/kg)	0.132±0.009	0.126±0.033**	0.135±0.022
Scutellarein(8mg/kg)	0.135±0.011	0.133±0.035*	0.155±0.025
**3**(8mg/kg)	0.123±0.007	0.122±0.032*	0.148±0.058
**7a**(8mg/kg)	0.138±0.007	0.160±0.048**	0.163±0.058
**7b**(8mg/kg)	0.120±0.014	0.118±0.036**	0.136±0.030
**7c**(8mg/kg)	0.123±0.005	0.110±0.011*	0.128±0.050#▲▲
**7d**(8mg/kg)	0.155±0.010	0.113±0.012***	0.193±0.067#
**7e**(8mg/kg)	0.127±0.005	0.126±0.035*	0.160±0.045*#
**7f**(8mg/kg)	0.137±0.005	0.101±0.003**	0.156±0.061
**7g**(8mg/kg)	0.132±0.011	0.106±0.004*	0.155±0.027
**8a**(8mg/kg)	0.148±0.021	0.111±0.018***	0.141±0.013
**10a**(8mg/kg)	0.140±0.024	0.120±0.036**	0.150±0.037
**10b**(8mg/kg)	0.128±0.016	0.108±0.010**	0.135±0.030
**10c**(8mg/kg)	0.130±0.016	0.130±0.044*	0.113±0.009▲
**10d**(8mg/kg)	0.128±0.021	0.140±0.046**	0.135±0.032
**10e**(8mg/kg)	0.125±0.008	0.141±0.039**	0.160±0.045
**10f**(8mg/kg)	0.138±0.012	0.186±0.037***	0.155±0.030
**11f**(8mg/kg)	0.153±0.027	0.211±0.123	0.176±0.029##▲
**12a**(8mg/kg)	0.123±0.005	0.133±0.042*	0.161±0.047*
**13a**(8mg/kg)	0.133±0.016	0.110±0.022**	0.171±0.060
**13b**(8mg/kg)	0.140±0.016	0.210±0.015***	0.163±0.030#
**14a**(8mg/kg)	0.132±0.009	0.108±0.010**	0.186±0.069#
**14b**(8mg/kg)	0.138±0.016	0.190±0.031**	0.183±0.038*##▲

Compared with Normal：*P＜0.05 **P＜0.01 ***P＜0.001

Compared with NS：#P < 0.05 ##P < 0.01 ###P < 0.001

Compared with DMSO：▲P < 0.05 ▲▲P < 0.01 ▲▲▲P < 0.001

Data represent mean ± SD. n = 6

S4. QRS of normal group, Pre-dose group and Post-dose group

Group	QRS （ms)		
	Normal	Pre-dose	Post-dose
NS	13.963±0.835	13.851±2.071	14.186±2.126
DMSO	13.923±1.079	16.351±1.862**	15.193±3.315
Ver(2mg/kg)	13.103±0.481	15.231±1.677**	13.811±0.874
Scutellarein(8mg/kg)	13.515±0.403	12.393±0.420	14.188±0.332
**3**(8mg/kg)	12.953±0.327	12.768±3.229	13.813±0.705
**7a**(8mg/kg)	13.366±0.704	14.766±2.445	15.140±1.911*
**7b**(8mg/kg)	14.373±0.969	16.540±1.687**	15.348±1.167
**7c**(8mg/kg)	14.185±1.117	15.456±2.034	16.611±1.956*##
**7d**(8mg/kg)	14.598±0.324	16.675±1.245	15.985±2.061
**7e**(8mg/kg)	14.111±0.581	15.986±2.301*	14.195±1.418
**7f**(8mg/kg)	14.356±0.458	16.768±1.386**	15.316±0.914
**7g**(8mg/kg)	14.410±0.277	16.343±2.100**	15.998±1.380#
**8a**(8mg/kg)	13.996±0.630	14.638±0.752	14.223±1.134
**10a**(8mg/kg)	13.851±0.760	15.158±0.873	14.673±1.305
**10b**(8mg/kg)	13.233±0.579	15.661±1.759**	13.796±1.631*
**10c**(8mg/kg)	13.868±0.885	16.185±2.267**	14.265±1.888
**10d**(8mg/kg)	14.080±1.529	16.333±2.128***	14.130±1.609
**10e**(8mg/kg)	13.536±0.466	16.185±1.000***	14.411±0.527
**10f**(8mg/kg)	14.708±0.823	17.343±0.869**	15.896±1.249
**11f**(8mg/kg)	14.183±0.665	15.850±0.943*	14.750±0.496
**12a**(8mg/kg)	13.216±0.625	15.451±2.057	14.021±1.026
**13a**(8mg/kg)	14.016±0.538	15.615±0.725*	17.175±1.010**#▲
**13b**(8mg/kg)	13.886±0.650	17.488±1.524***	15.101±0.393*
**14a**(8mg/kg)	14.615±0.756	16.796±1.208**	15.850±1.200
**14b**(8mg/kg)	13.773±0.662	16.088±0.854**	15.126±0.621*

Compared with Normal：*P＜0.05 **P＜0.01 ***P＜0.001

Compared with NS：#P < 0.05 ##P < 0.01 ###P < 0.001

Compared with DMSO：▲P < 0.05 ▲▲P < 0.01 ▲▲▲P < 0.001

S5. Investigated compounds induced alterations of HR(bpm), QT(ms), QTc-Bazett's and RR (ms) in anesthetized rats

Group	Time interval（min）						
	3	5	10	15	20	25	30
HR(bpm)							
NS	504.41±45.93	496.83±42.13	475.27±27.05	463.37±29.18	458.60±36.01	445.78±23.75	445.78±23.75
DMSO	466.34±44.32	503.84±46.31	480.76±19.23	480.76±19.23	480.76±19.23	480.76±19.23	468.86±24.91
$BaCl_2$	600±0	600±0	535.66±56.94	534.49±77.89	544.75±61.92	499.51±57.91	501.16±62.63
**10e**(8mg/kg)	418.68±27.59▲▲	482.85±37.33▲▲	480.76±19.23	461.53±0	462.45±20.66	467.94±14.33	461.53±0
QT(ms)							
NS	38.73±1.09	39.08±2.66	39.18±0.69	38.78±0.89	38.78±3.21	39.96±1.80	40.01±3.51
DMSO	38.24±1.24	39.69±2.74	38.96±1.85	38.73±2.49	38.91±2.49	38.66±2.58	38.56±2.83
$BaCl_2$	47.17±6.32	48.65±3.50	46.45±5.51	43.93±3.71	45.98±3.46	44.64±4.23	46.72±4.08
**10e**(8mg/kg)	39.57±1.21▲	39.38±2.43▲▲	39.57±3.03▲▲	38.69±1.60▲▲	38.69±3.56▲▲	41.39±3.47	39.51±3.05▲
QTc(ms)							
NS	0.11±0.005	0.11±0.009	0.11±0.004	0.10±0.003	0.10±0.01	0.10±0.006	0.10±0.01
DMSO	0.10±0.01	0.11±0.01	0.11±0.006	0.10±0.008	0.11±0.009	0.11±0.009	0.10±0.009
$BaCl_2$	0.14±0.02	0.15±0.01	0.13±0.02	0.13±0.02	0.13±0.01	0.12±0.01	0.13±0.01
**10e**(8mg/kg)	0.11±0.003▲▲	0.11±0.01▲▲	0.11±0.01▲	0.10±0.004	0.10±0.01▲▲	0.11±0.01	0.10±0.008▲
RR(S)							
NS	0.12±0.012	0.12±0.01	0.13±0.007	0.13±0.008	0.13±0.01	0.14±0.007	0.14±0.008
DMSO	013±0.014	0.12±0.01	0.13±0.005	0.13±0.005	0.13±0.005	0.13±0.005	0.13±0.007
$BaCl_2$	0.10±0.004	0.10±0.004	0.11±0.012	0.12±0.012	0.11±0.013	0.12±0.01	0.12±0.02
**10e**(8mg/kg)	0.125±0.008▲▲	0.13±0.01▲▲▲	0.13±0.005▲▲▲	0.13±0	0.13±0.006▲	0.13±0.004	0.13±0

Compared with NS：*P < 0.05 **P < 0.01 ***P < 0.001

Compared with DMSO：+P＜0.05 ++P＜0.01 +++P＜0.001

Compared with $BaCl_2$：▲P＜0.05 ▲▲P＜0.01 ▲▲▲P＜0.001

S6. Patch clamp assay for Cav1.2

HEK Cav1.2 current (pA)						
Bath solution	**10e**(0.3μM)	**10e**(1μM)	**10e**(3μM)	**10e**(10μM)	**10e**(30μM)	Nifedipine(1μM)
-700.528	-750.45	-719.5	-647.9	-589.214	-432.85	-6.873
-453.409	-489.37	-495.73	-478.42	-394.5	-326.05	-12.73
-518.48	-540.46	-528.22	-497.23	-446.6	-355.49	-12.482

Current inhibition(%)					
**10e**(0.3μM)	**10e**(1μM)	**10e**(3μM)	**10e**(10μM)	**10e**(30μM)	Nifedipine 1μM
-7.13%	-2.71%	7.51%	15.89%	38.21%	99.02%
-7.93%	-9.33%	-5.52%	12.99%	28.09%	97.19%
-4.24%	-1.88%	4.10%	13.86%	31.44%	97.59%
Average%					
-6.43%	-4.64%	2.03%	14.25%	32.58%	97.93%
SD					
1.94%	4.09%	6.76%	1.49%	5.16%	0.96%

S7. Patch clamp assay for Nav1.5

	Control	**10e**（1μM）	**10e**（30μM）
N	7	9	8
Activation			
$V_{1/2}$	-33.6±1.1	-34.2±0.9（p=0.6535)	-30.1±0.9(p=0.0263)*
k	8.6±0.9	8.5±0.8(p=0.9309)	8.1±0.8(p=0.7241)
Inactivation			
$V_{1/2}$	-84.4±0.6	-80.8±0.4(p=0.0002)***	-94.1±1.7(p=0.0002)***
k	-7.7±0.5	-6.6±0.4	-10.9±1.6

Compared with control group：*P<0.05, **P<0.01, ***P<0.001

S8. Morphological changes in H9c2 cells following administration of 10e.

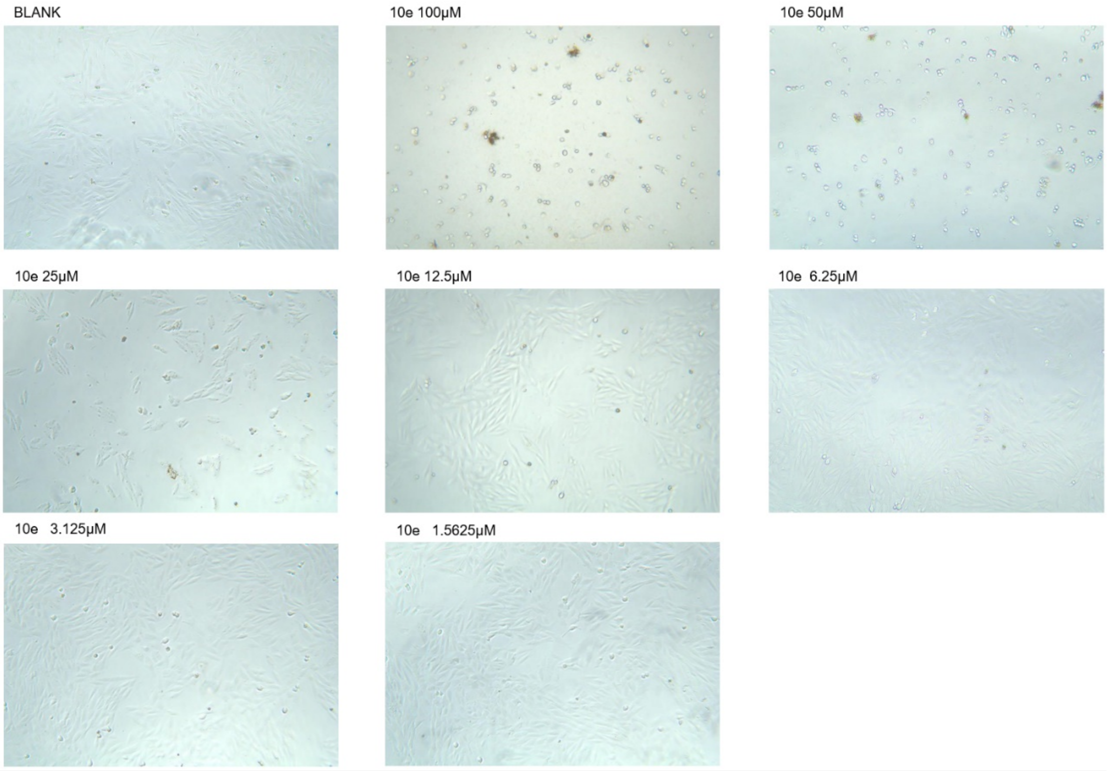

S9. ^{1}H NMR and ^{13}C NMR spectrum of target compounds

^{1}H NMR spectrum of compound **2**

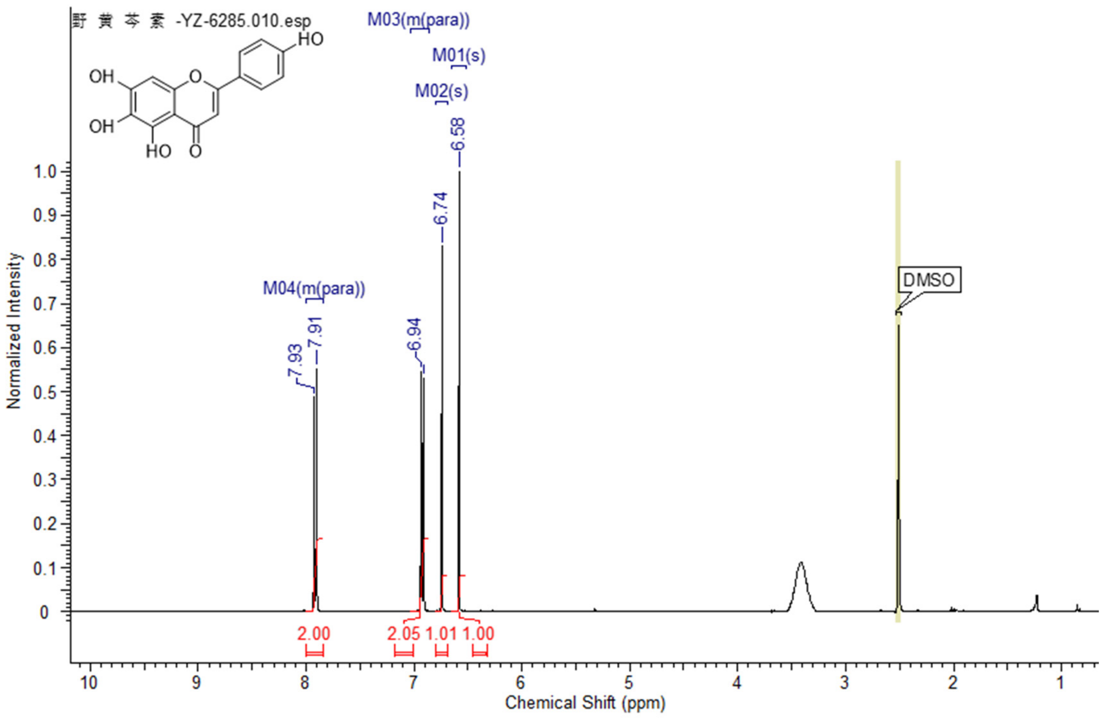

^{13}C NMR spectrum of compound **2**

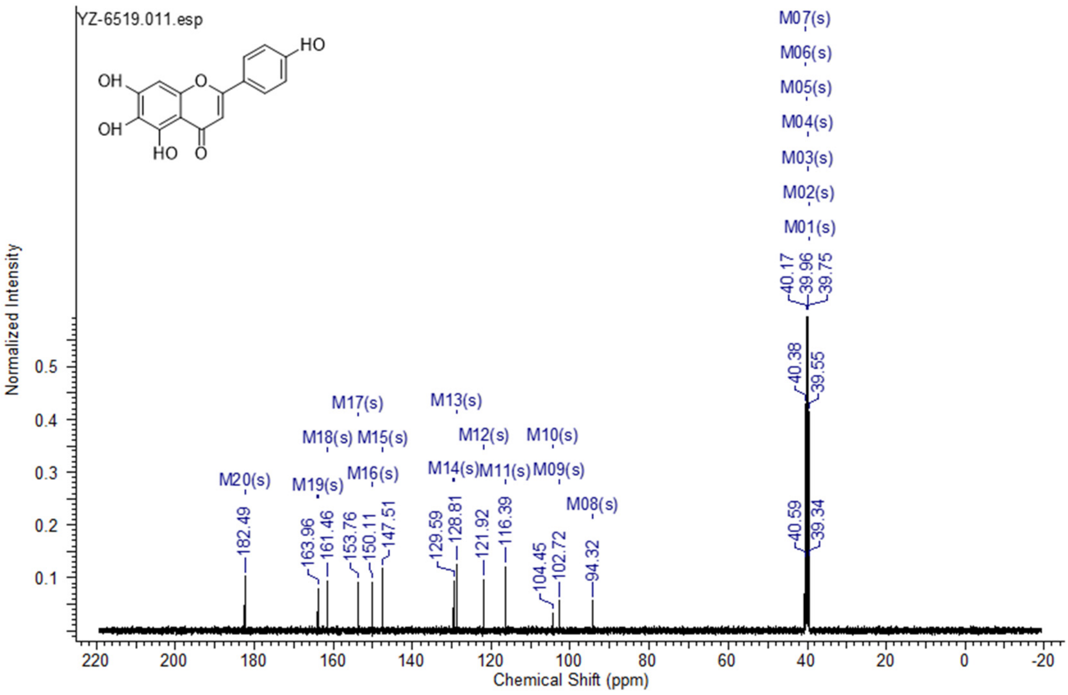

^{1}H NMR spectrum of compound **3**

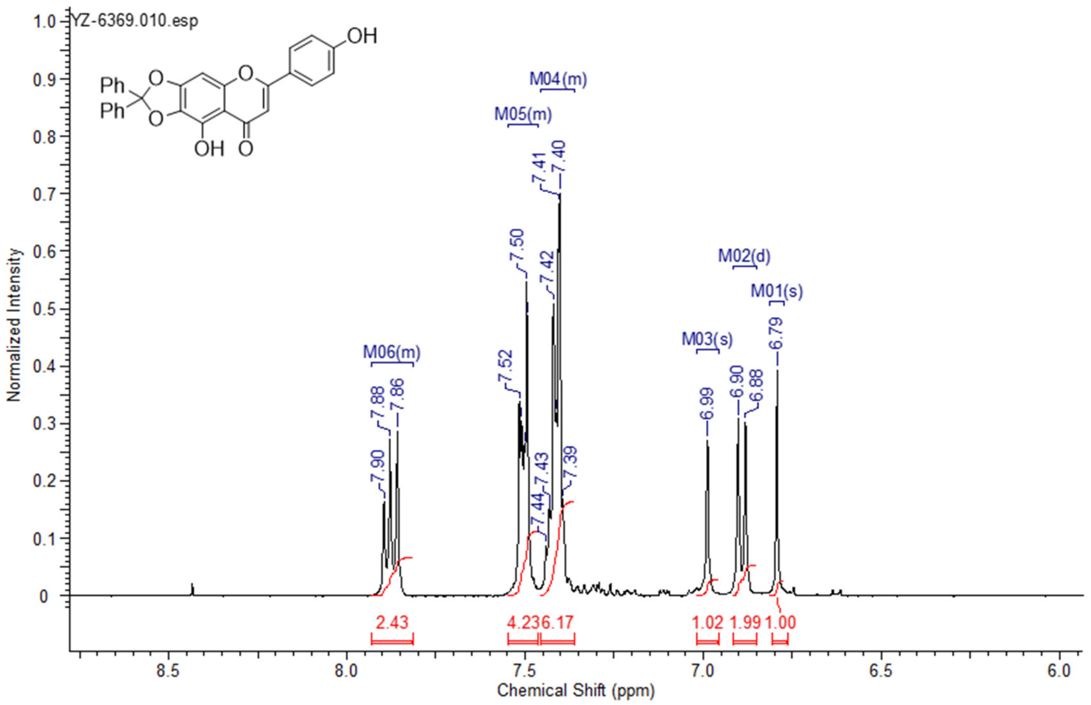

^{13}C NMR spectrum of compound **3**

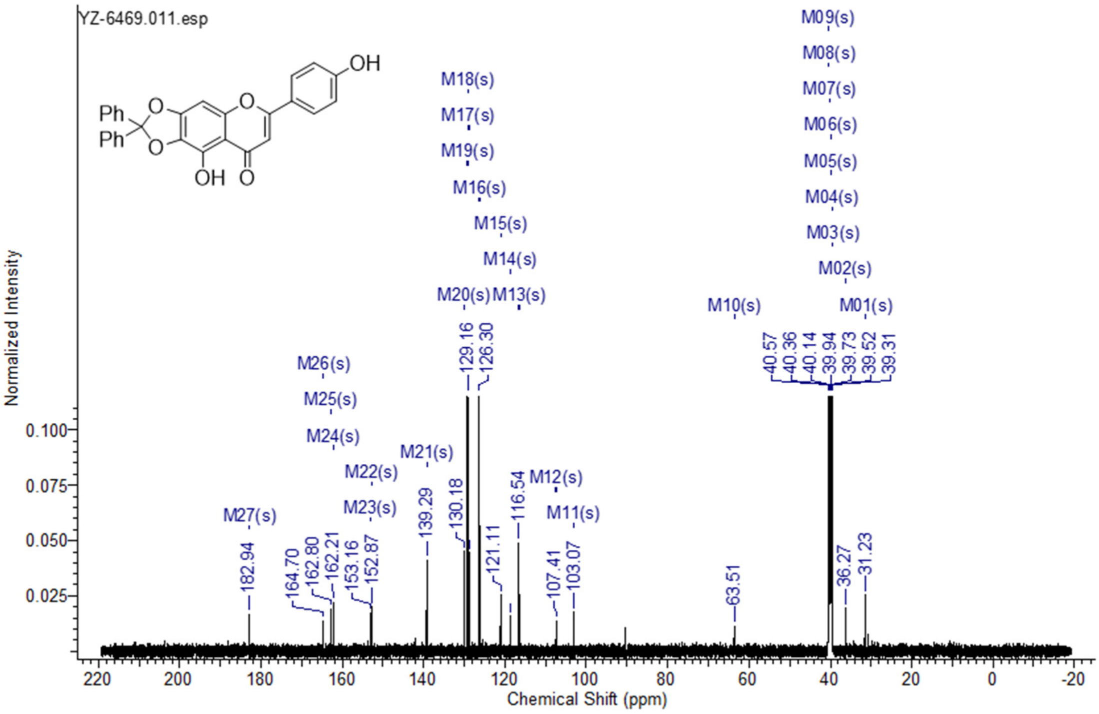

^{1}H NMR spectrum of compound **7a**

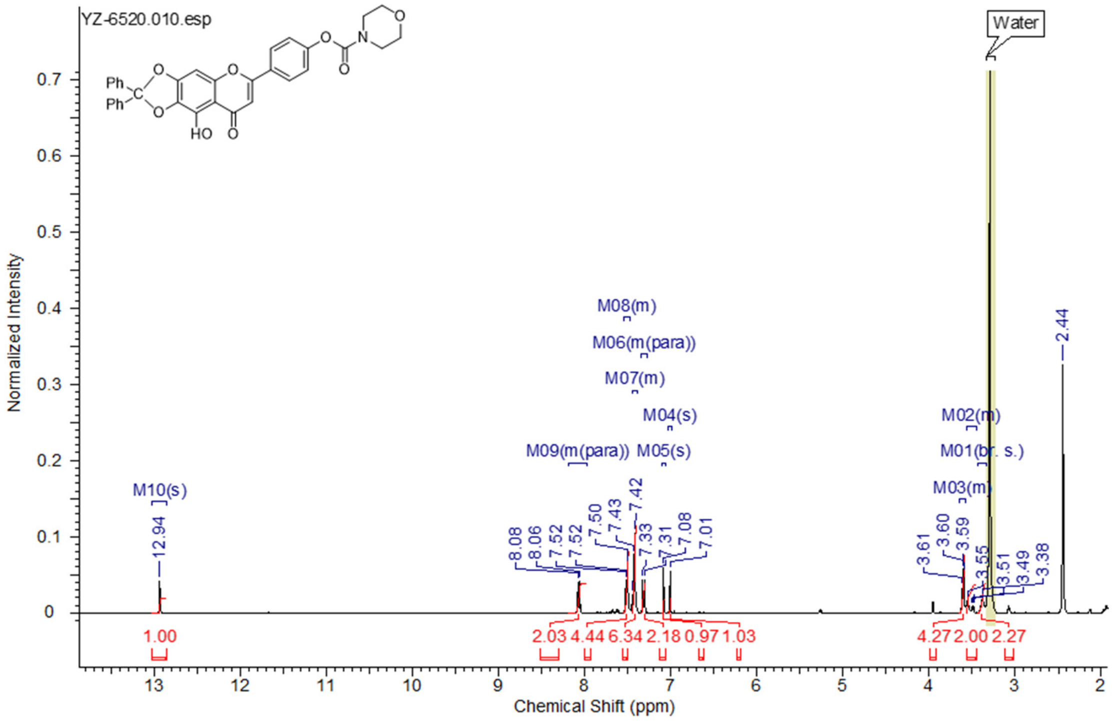

^{13}C NMR spectrum of compound **7a**

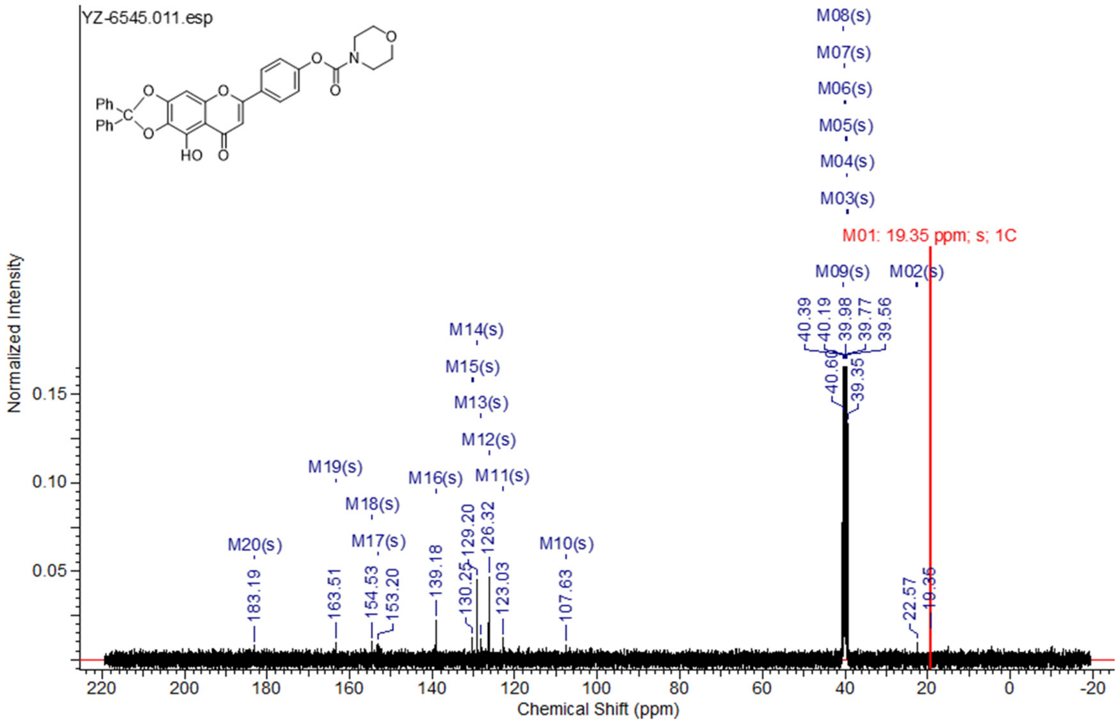

^{1}H NMR spectrum of compound **7b**

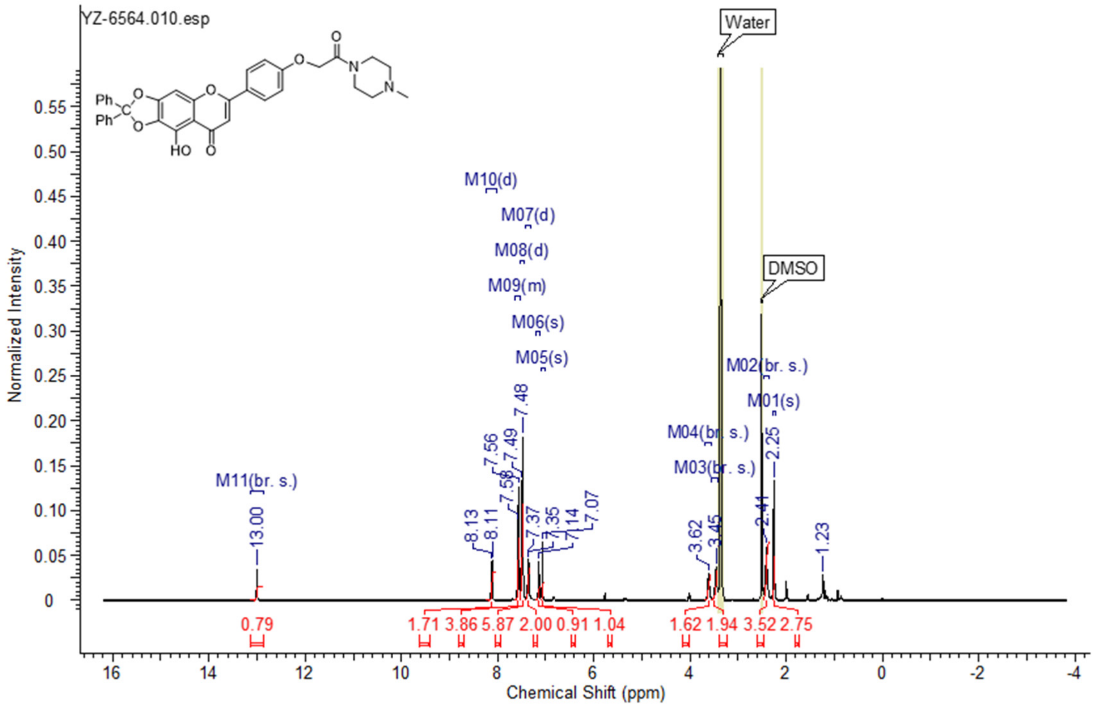

^{13}C NMR spectrum of compound **7b**

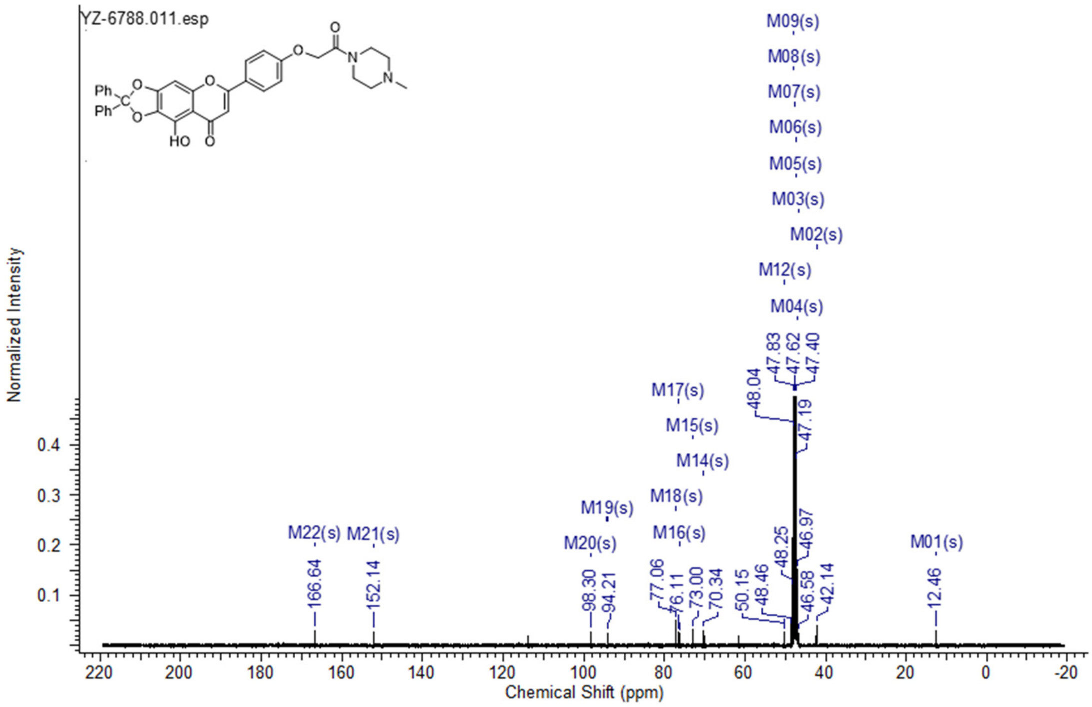

^{1}H NMR spectrum of compound **7c**

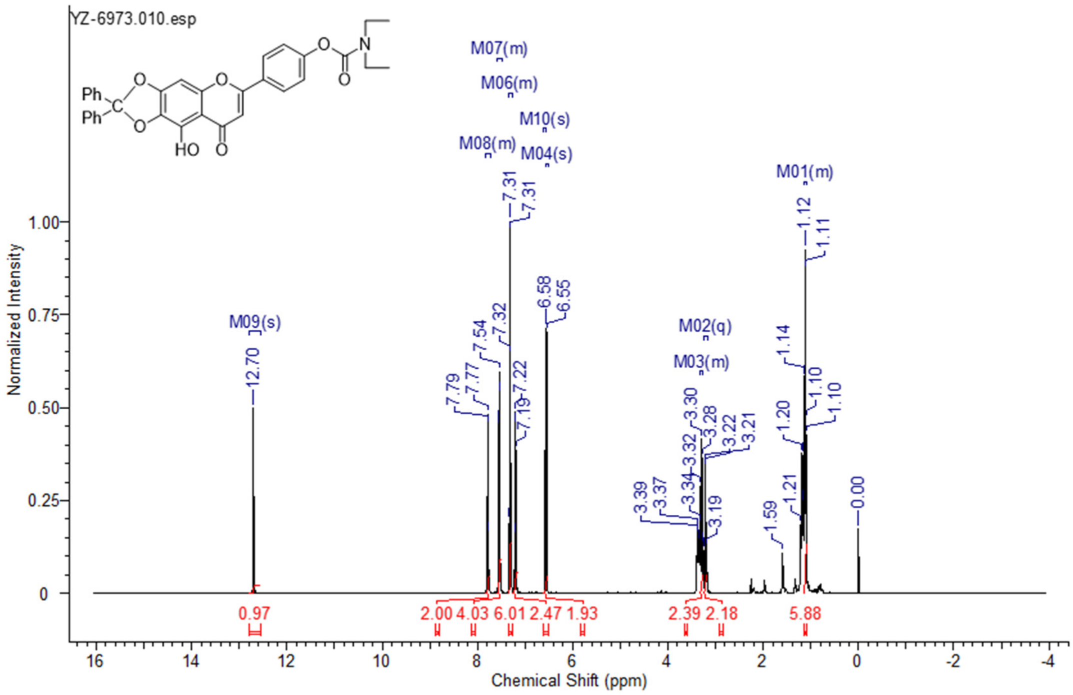

^{13}C NMR spectrum of compound **7c**

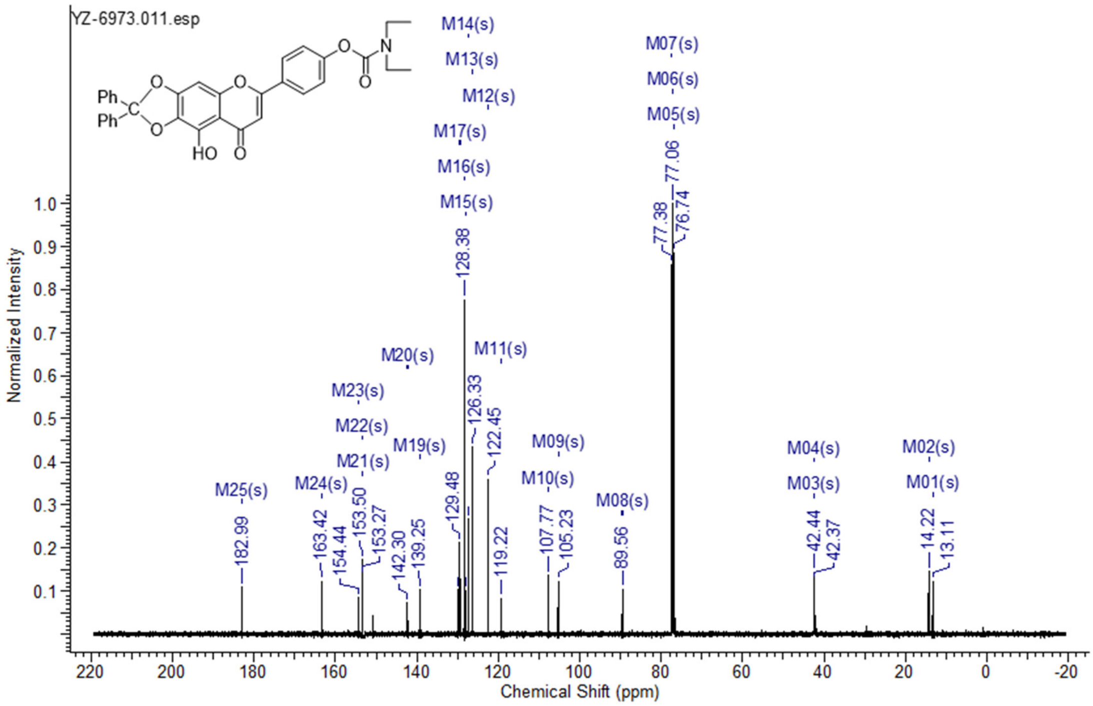

^{1}H NMR spectrum of compound **7d**

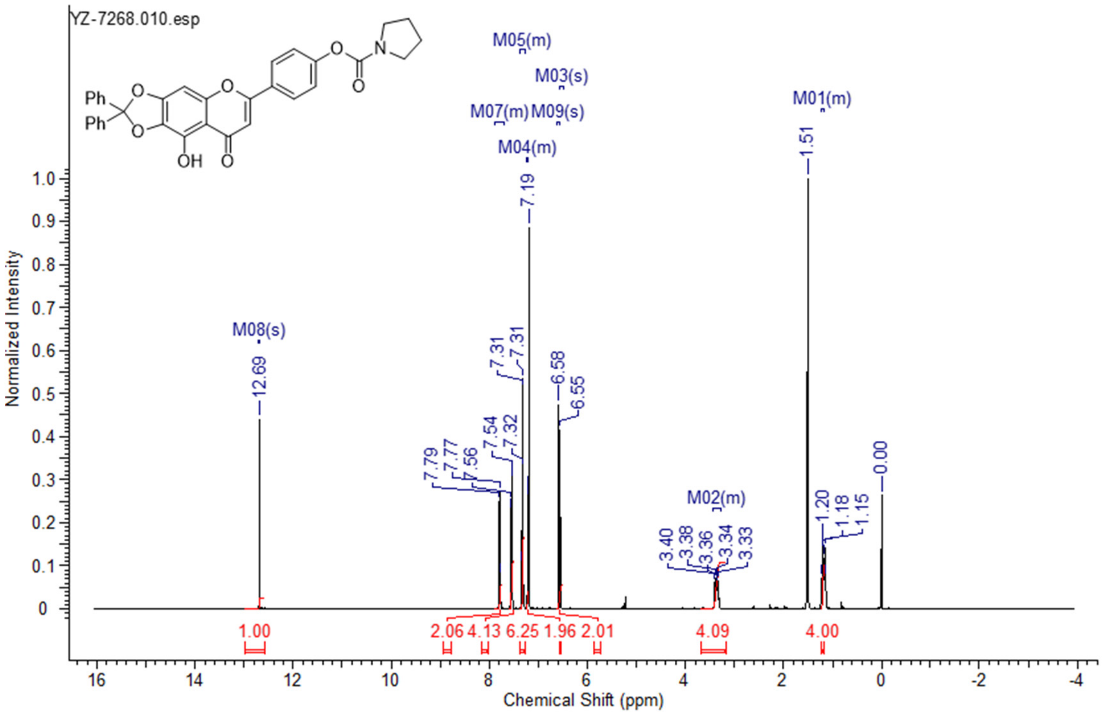

^{13}C NMR spectrum of compound **7d**

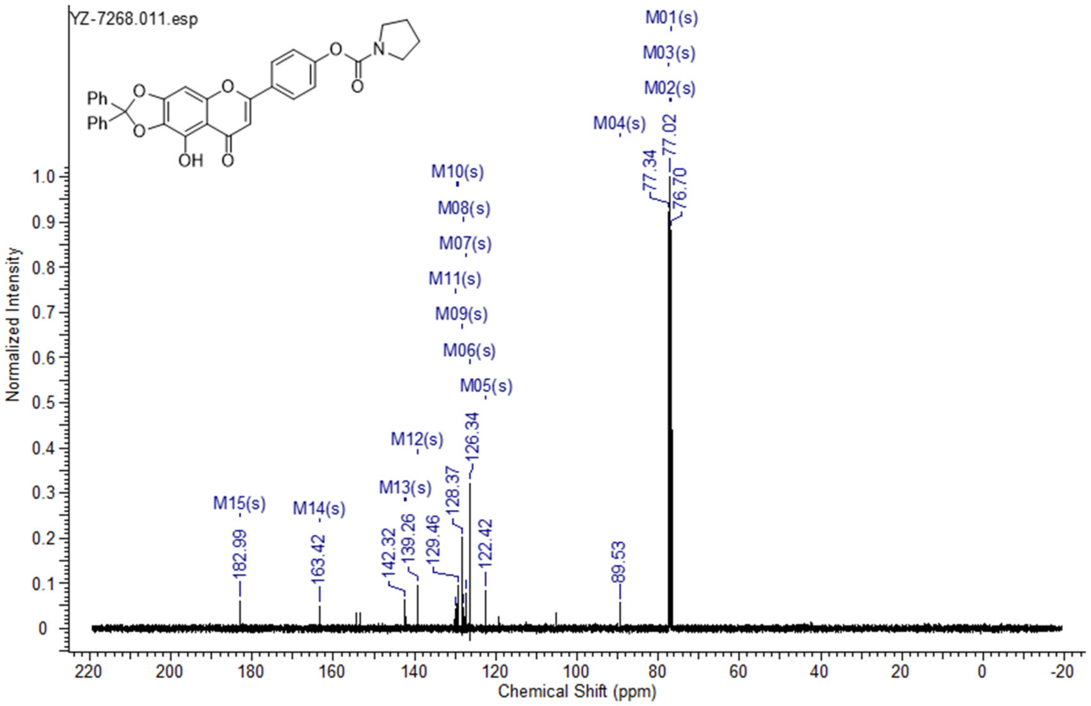

^{1}H NMR spectrum of compound **7e**

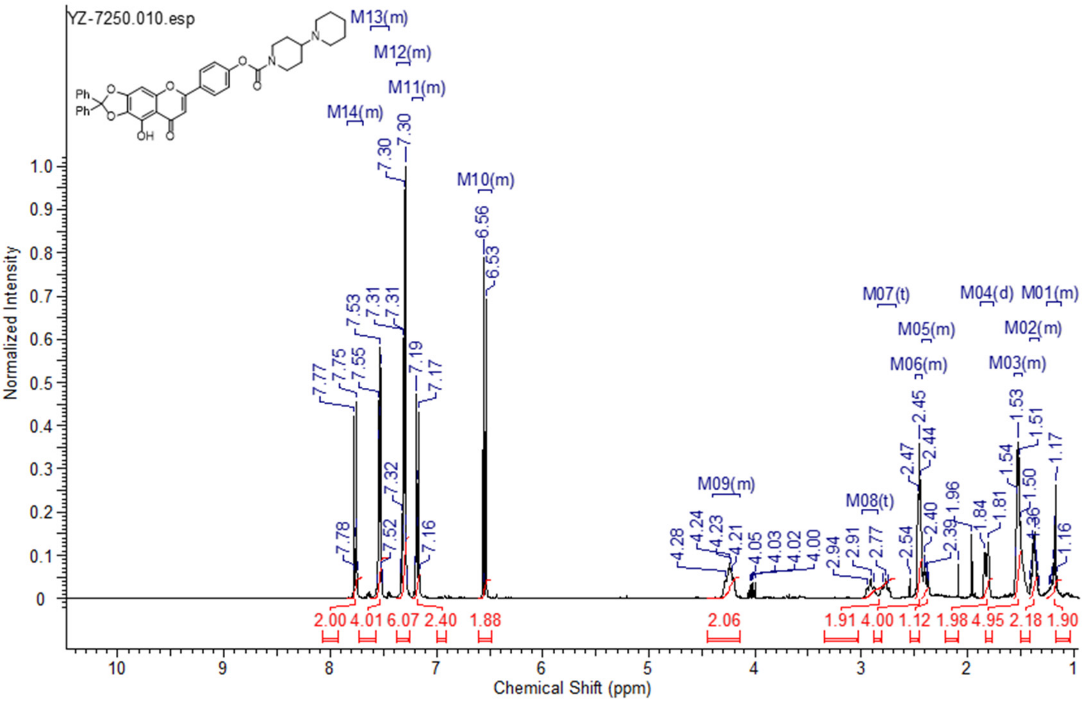

^{13}C NMR spectrum of compound **7e**

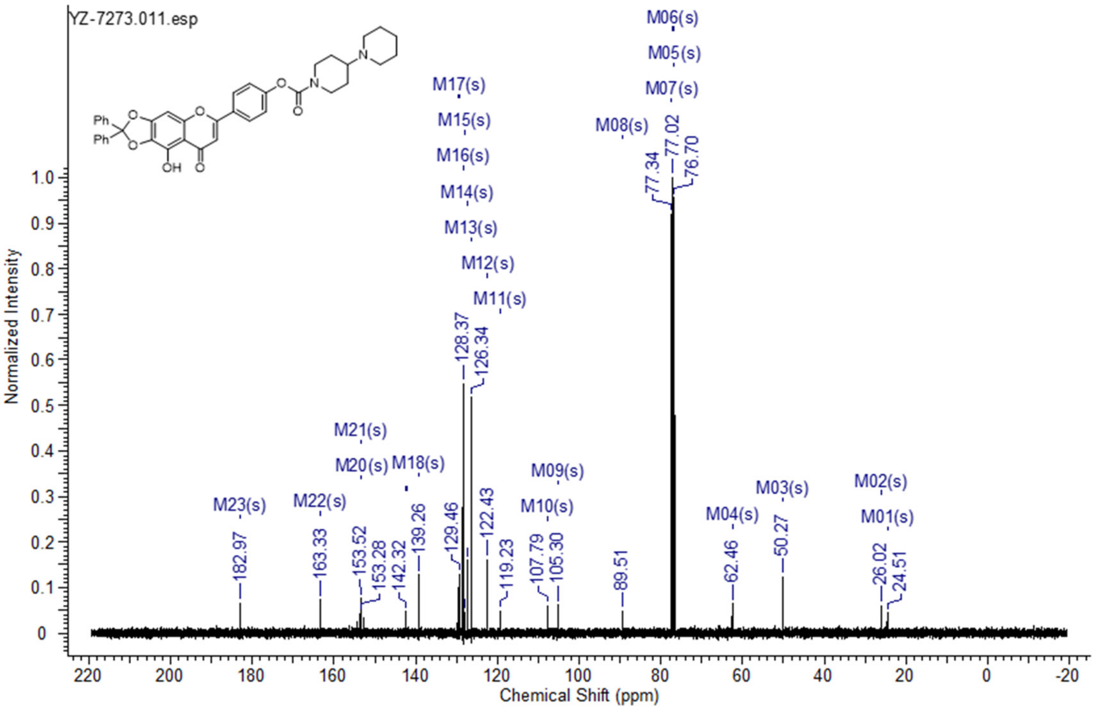

^{1}H NMR spectrum of compound **7f**

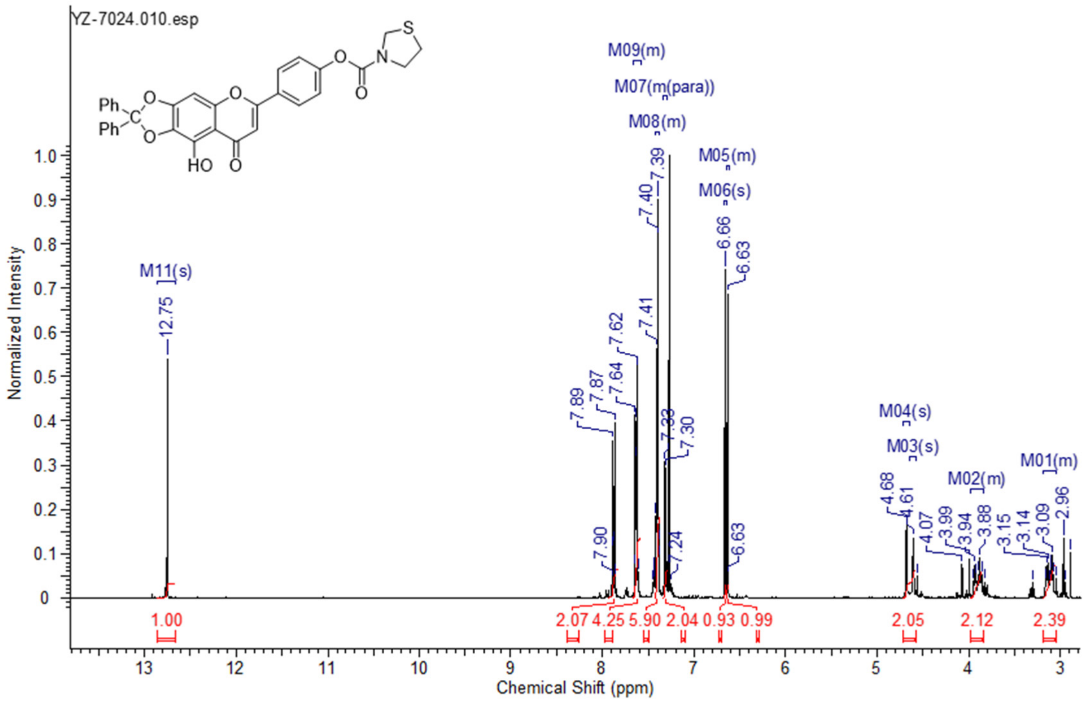

^{13}C NMR spectrum of compound **7f**

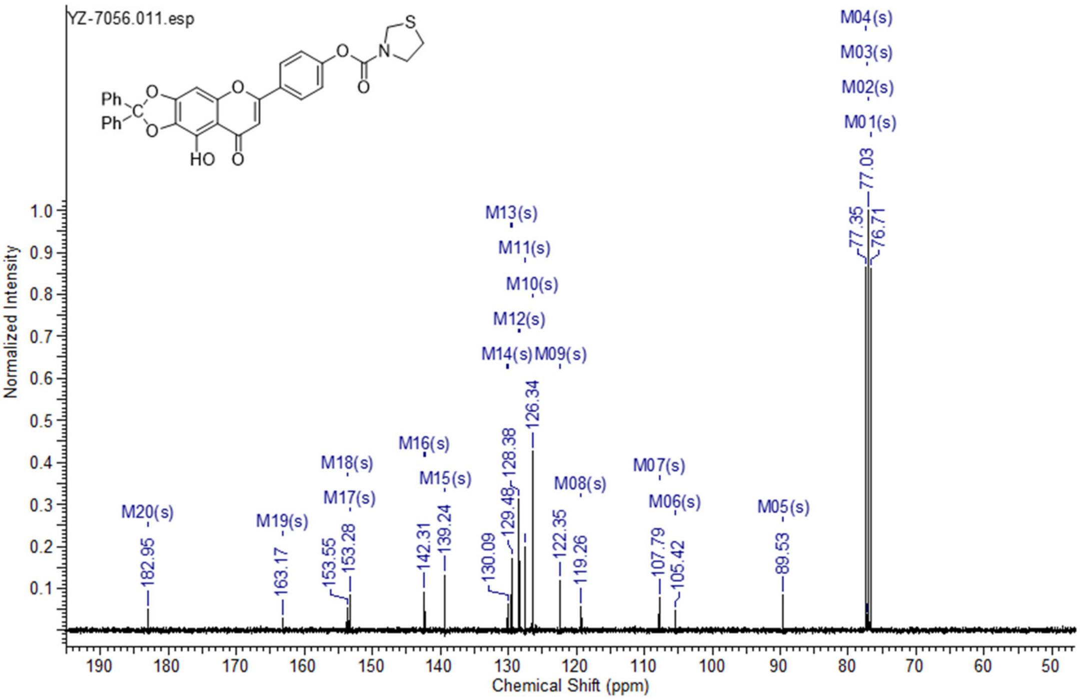

^{1}H NMR spectrum of compound **7g**

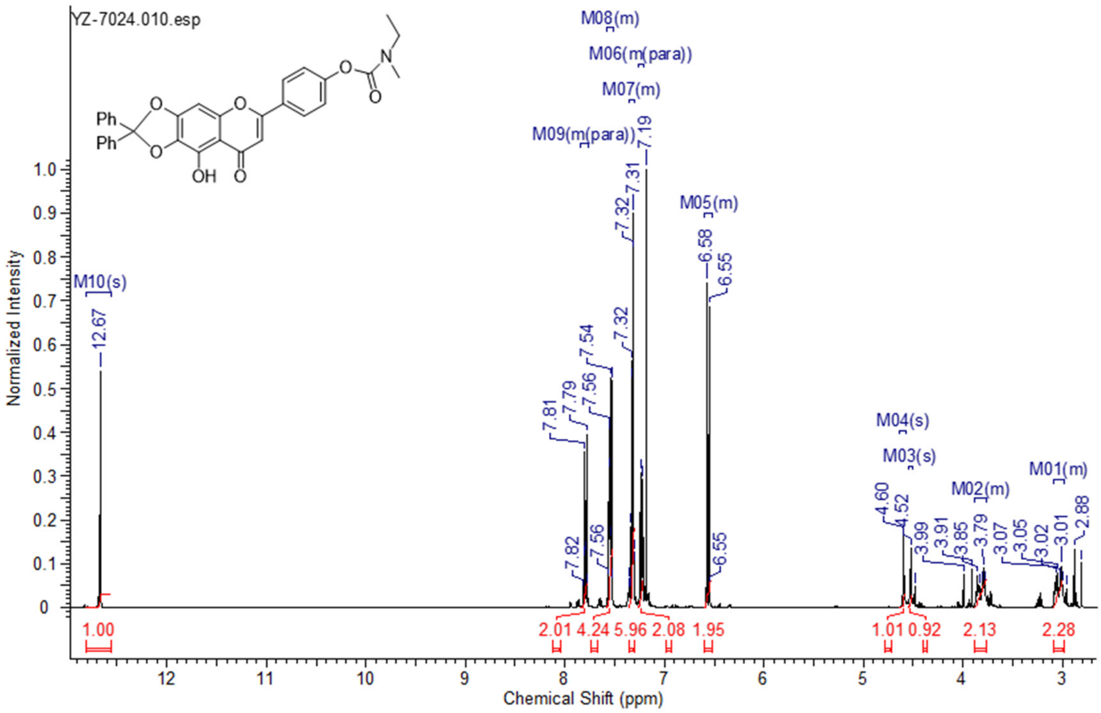

^{13}C NMR spectrum of compound **7g**

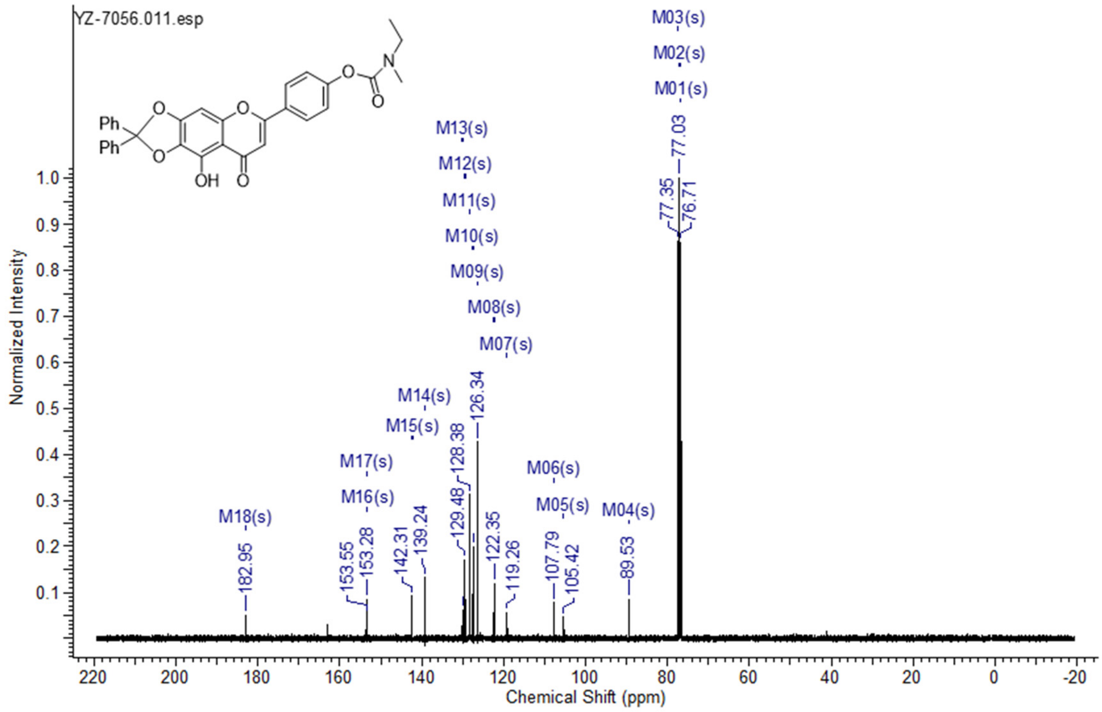

^{1}H NMR spectrum of compound **8a**

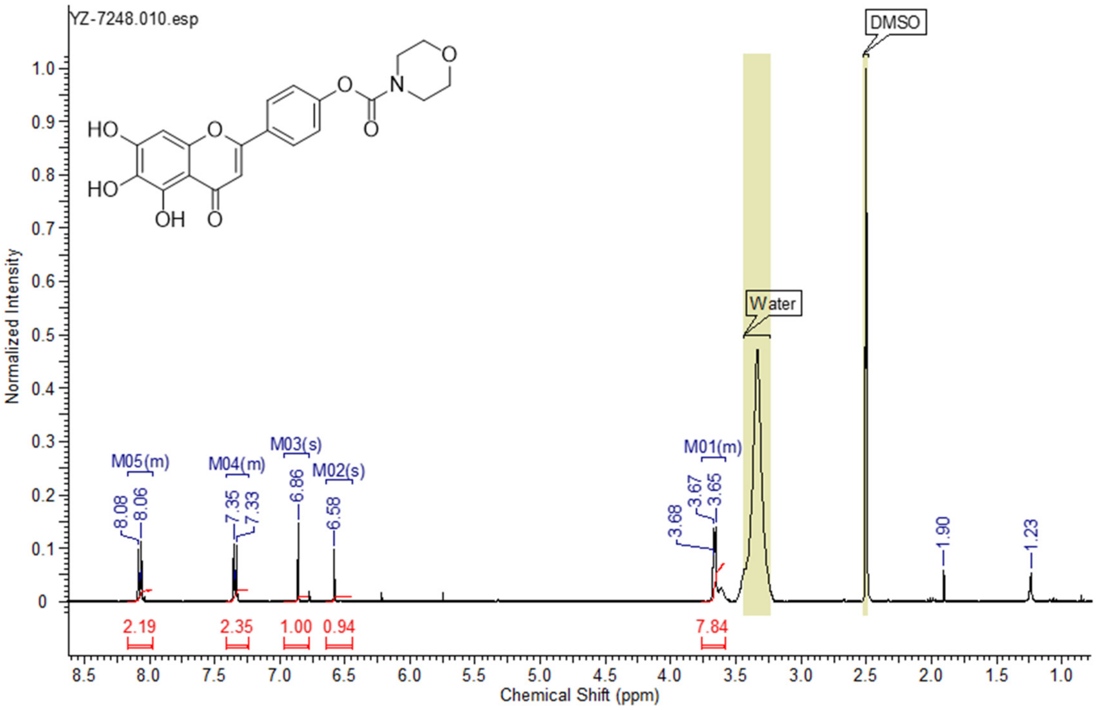

^{1}H NMR spectrum of compound **10a**

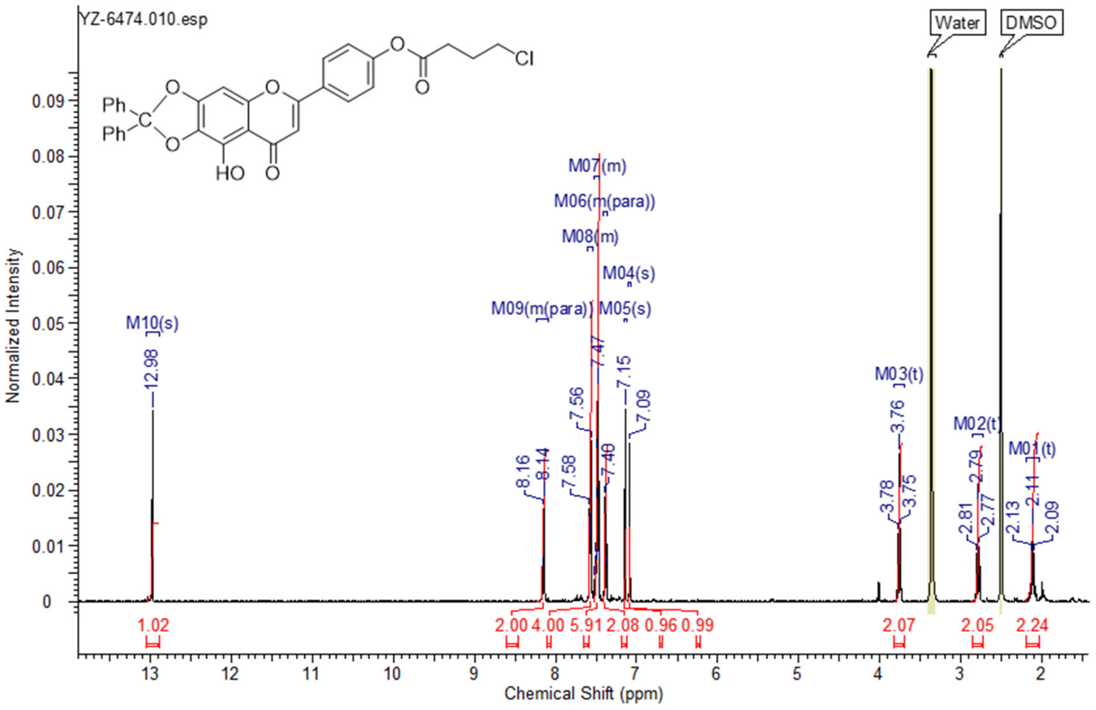

^{13}C NMR spectrum of compound **10a**

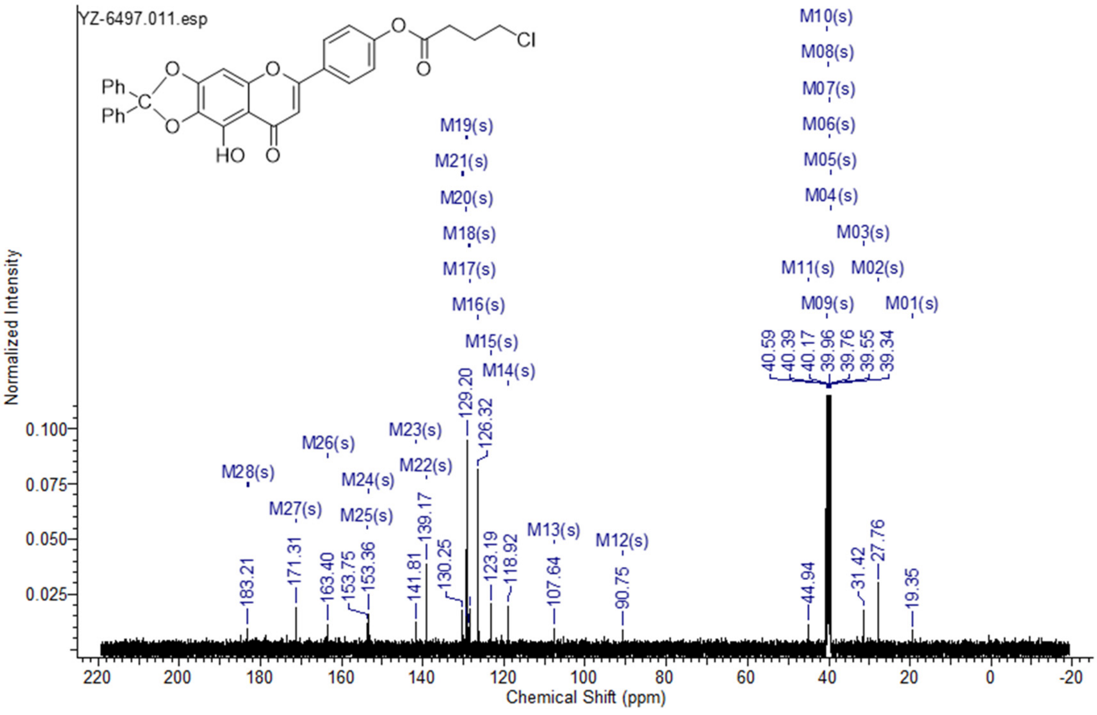

^{1}H NMR spectrum of compound **10b**

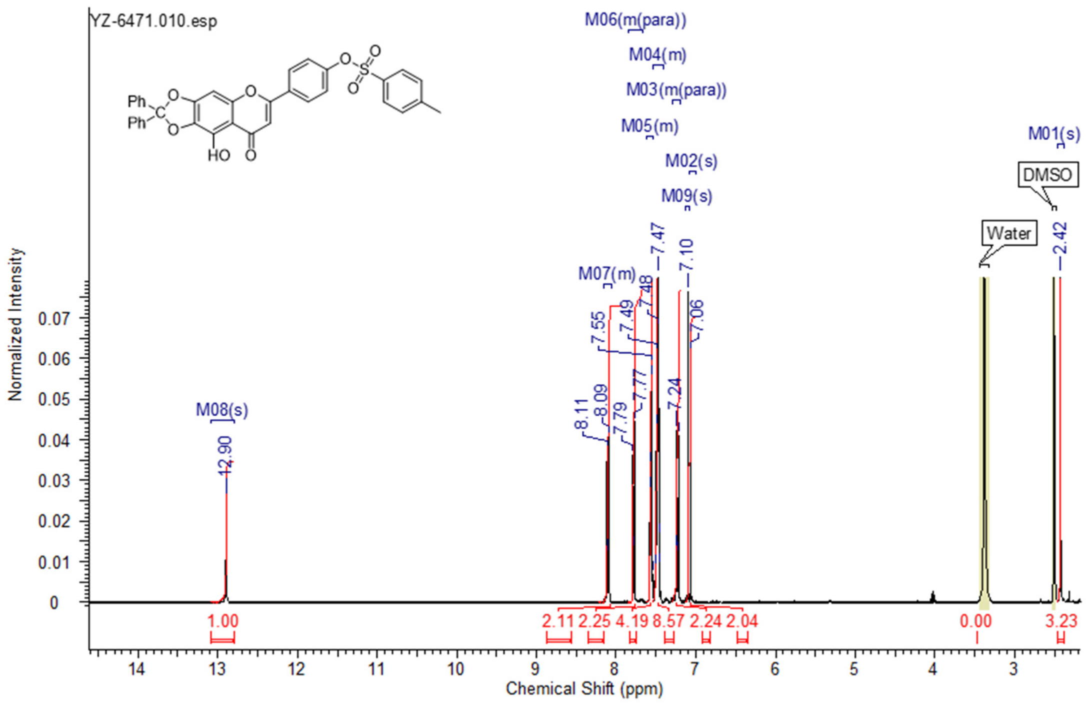

^{13}C NMR spectrum of compound **10b**

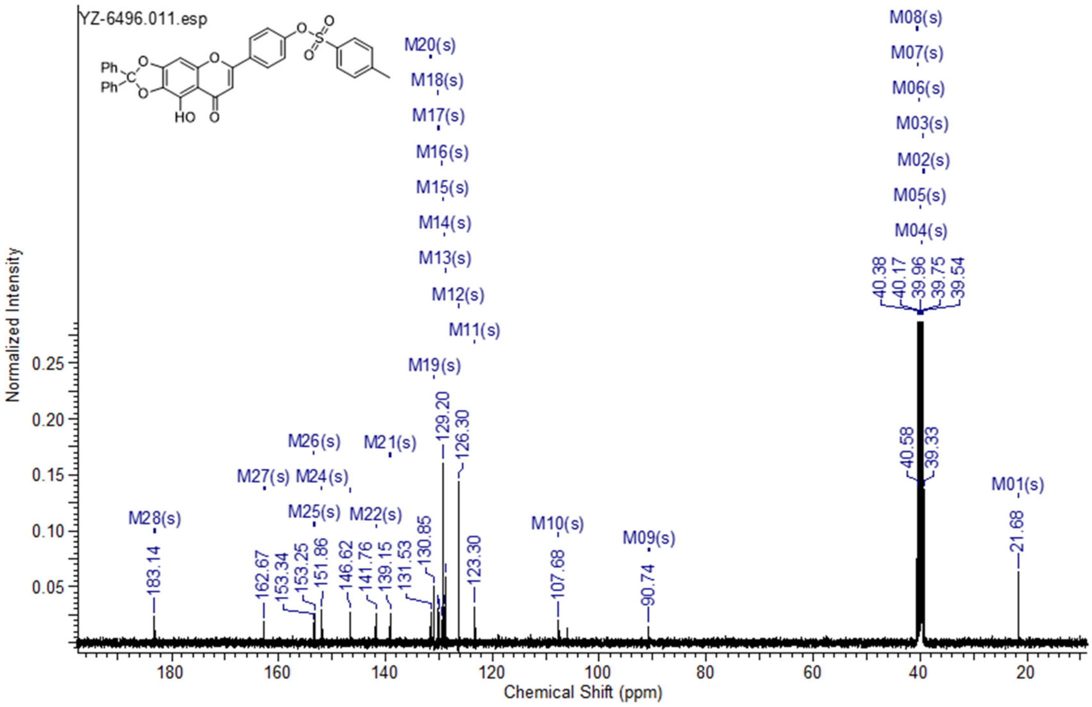

^{1}H NMR spectrum of compound **10c**

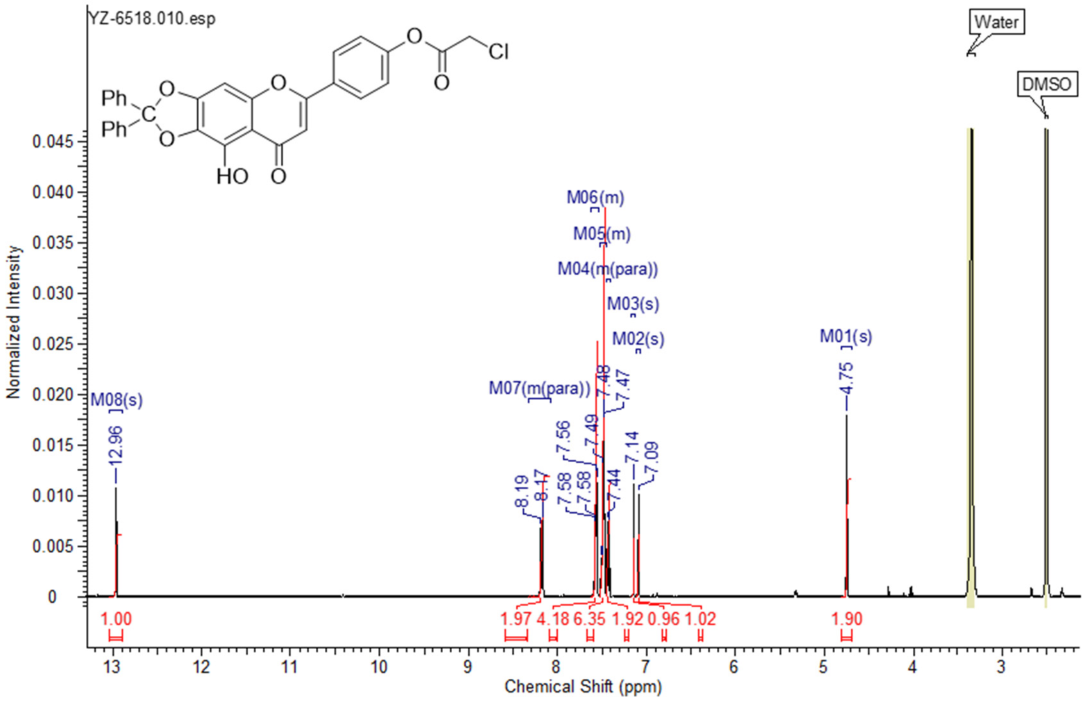

^{13}C NMR spectrum of compound **10c**

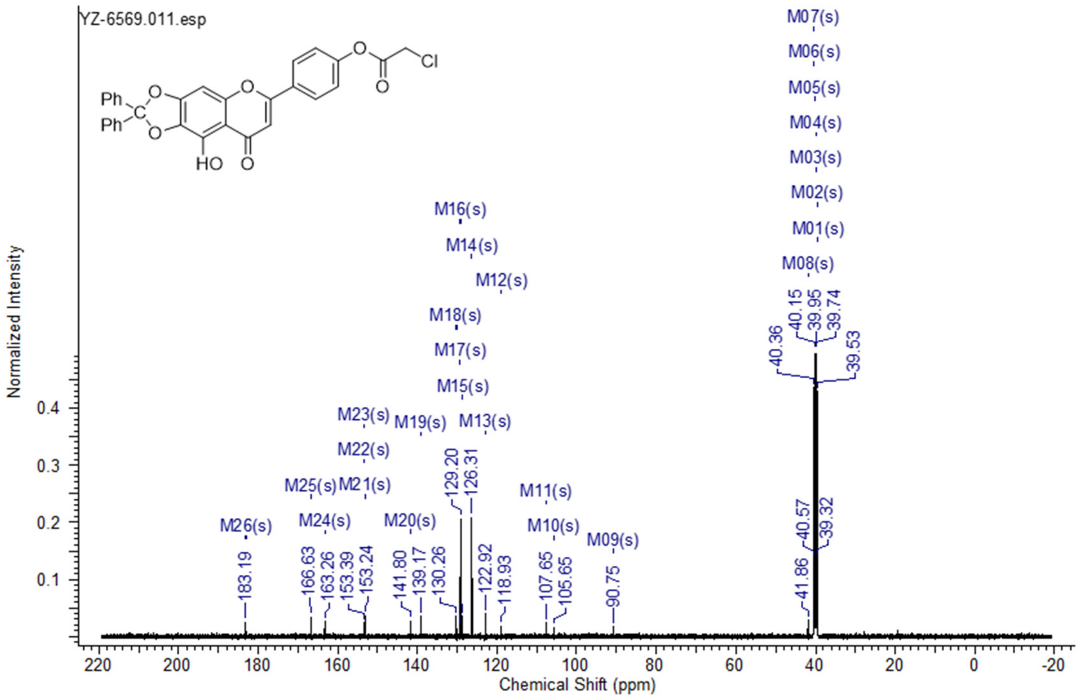

^{1}H NMR spectrum of compound **10d**

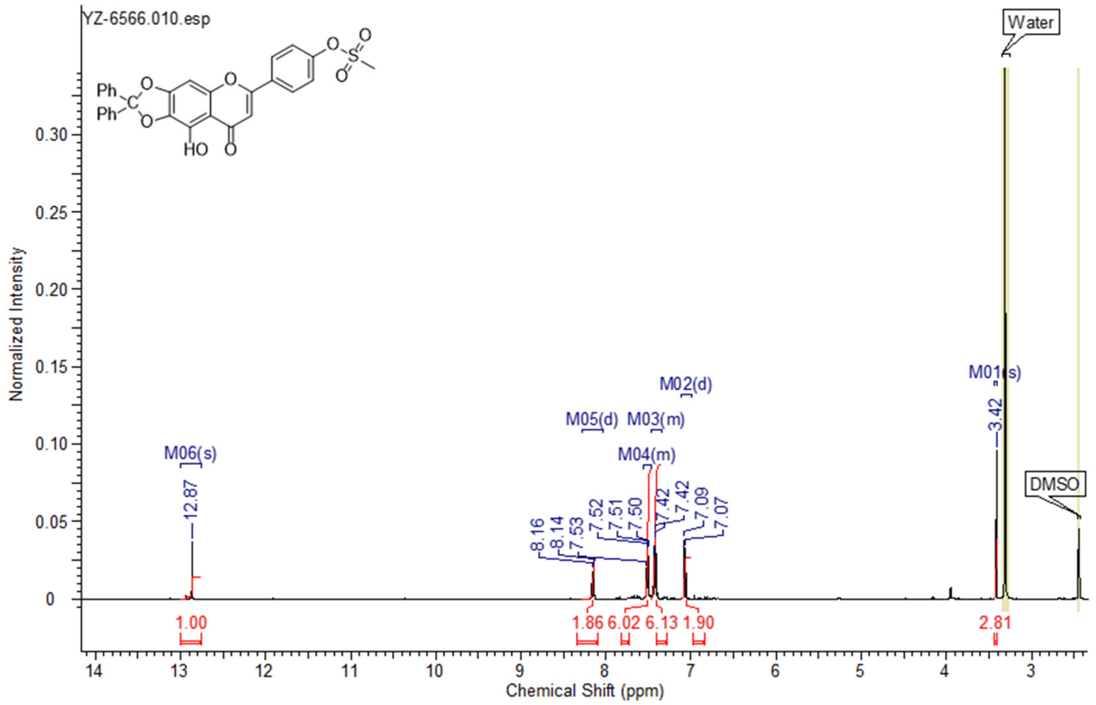

^{13}C NMR spectrum of compound **10d**

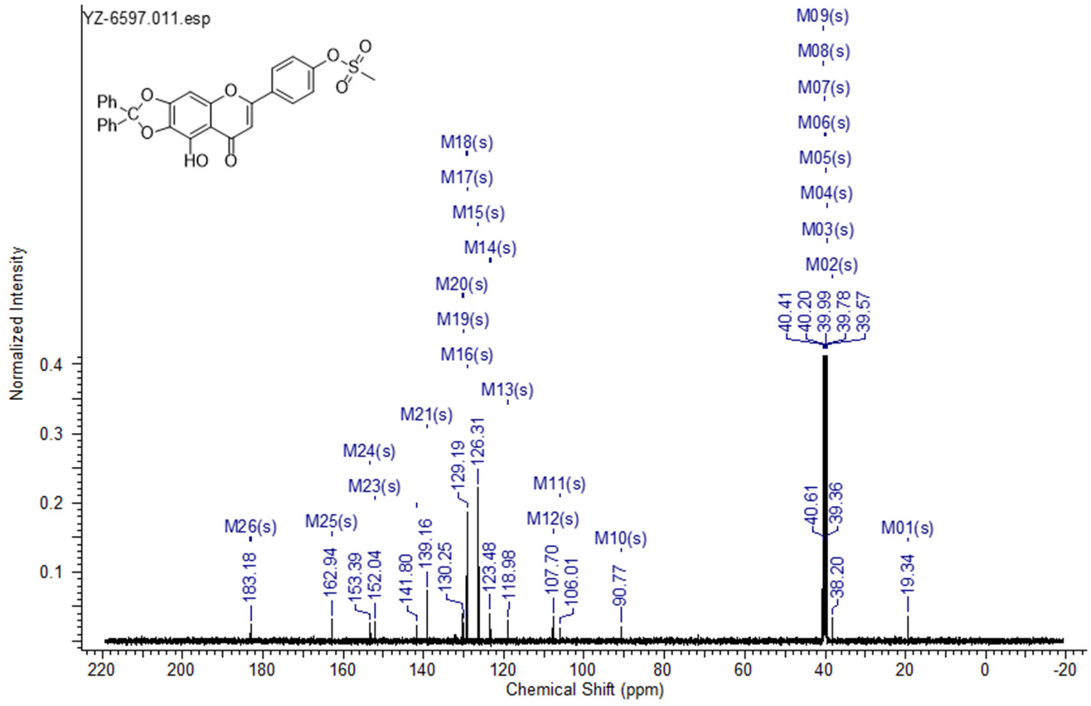

^{1}H NMR spectrum of compound **10e**

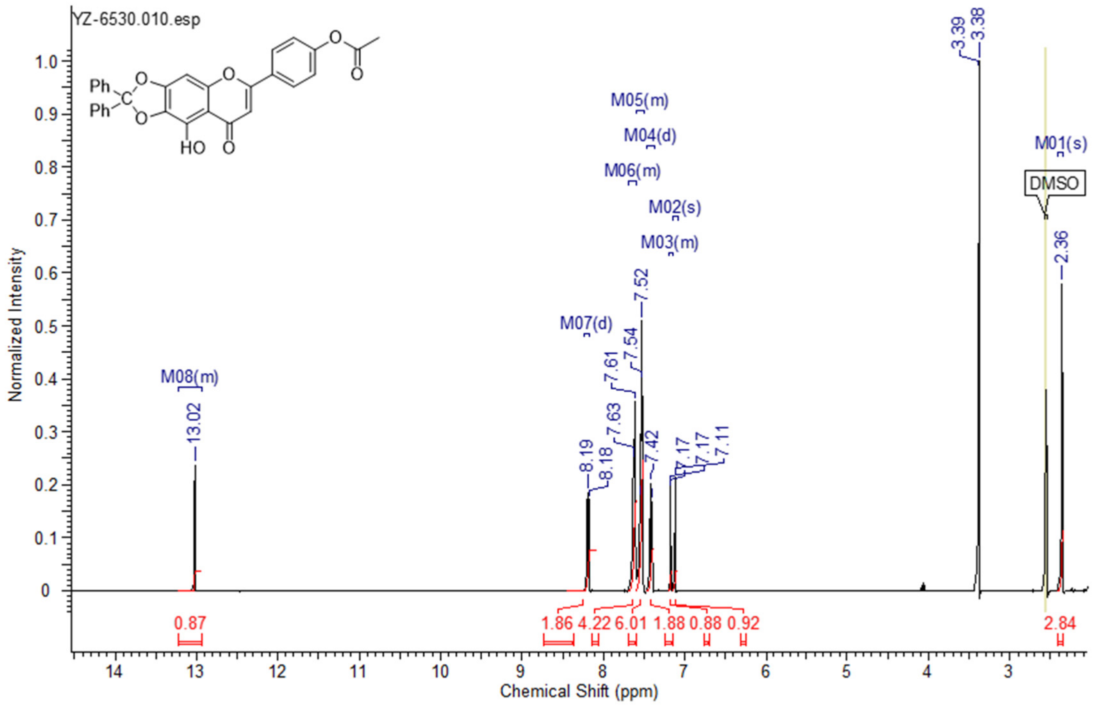

^{13}C NMR spectrum of compound **10e**

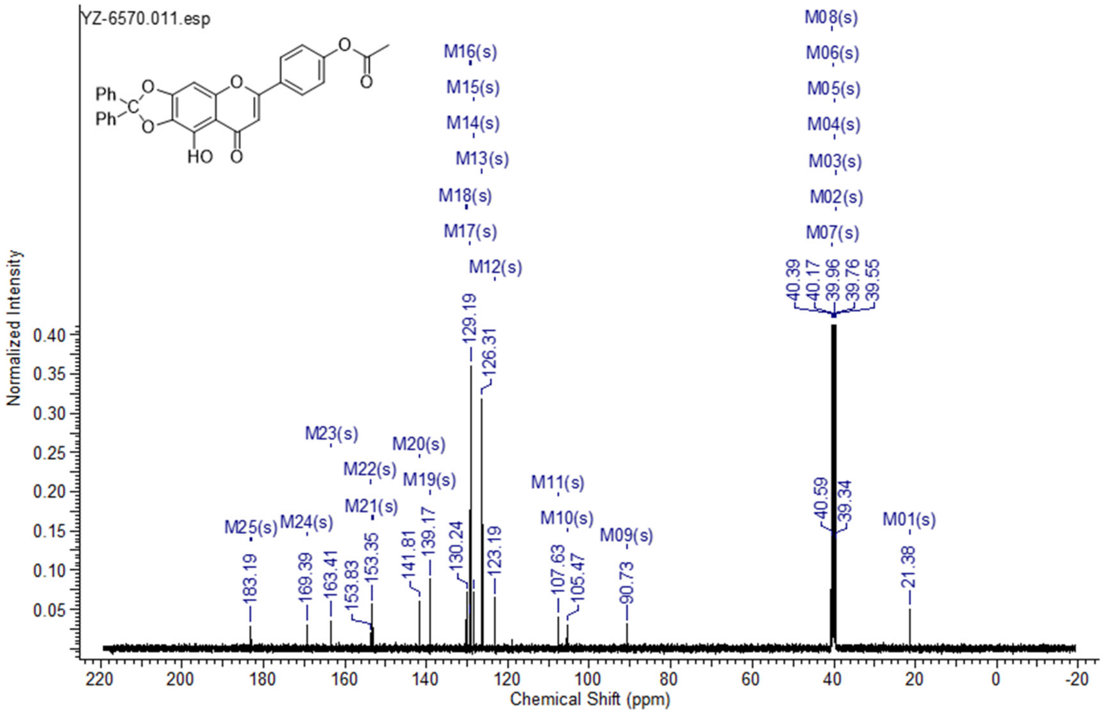

^{1}H NMR spectrum of compound **10f**

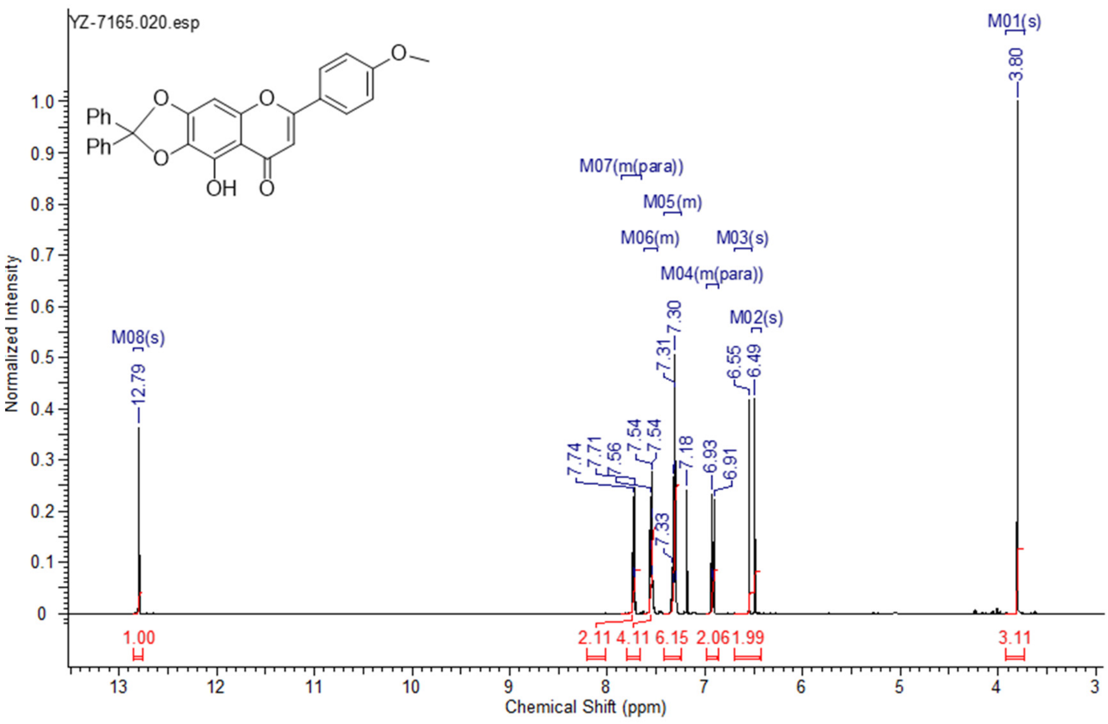

^{13}C NMR spectrum of compound **10f**

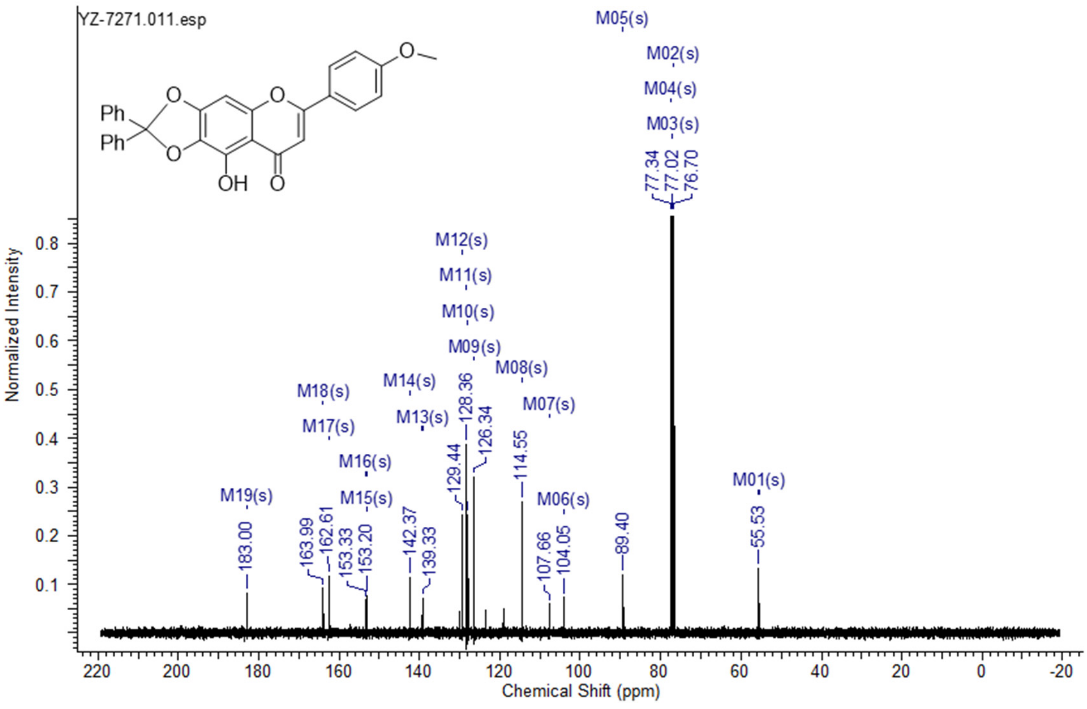

^{1}H NMR spectrum of compound **11f**

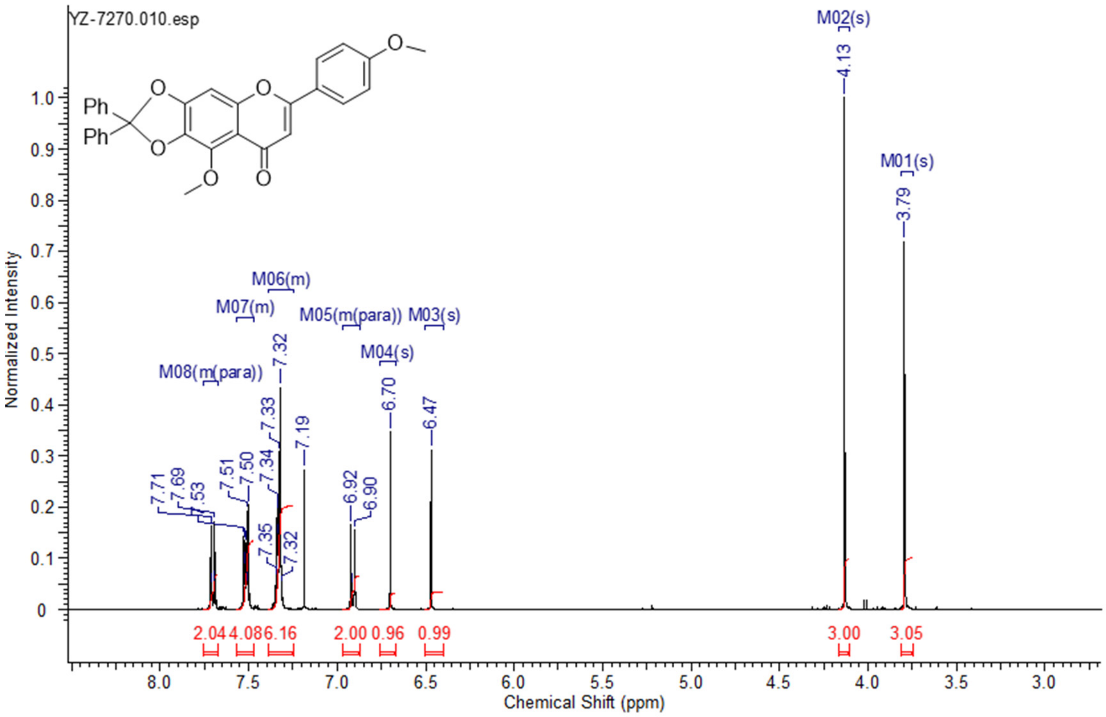

^{13}C NMR spectrum of compound **11f**

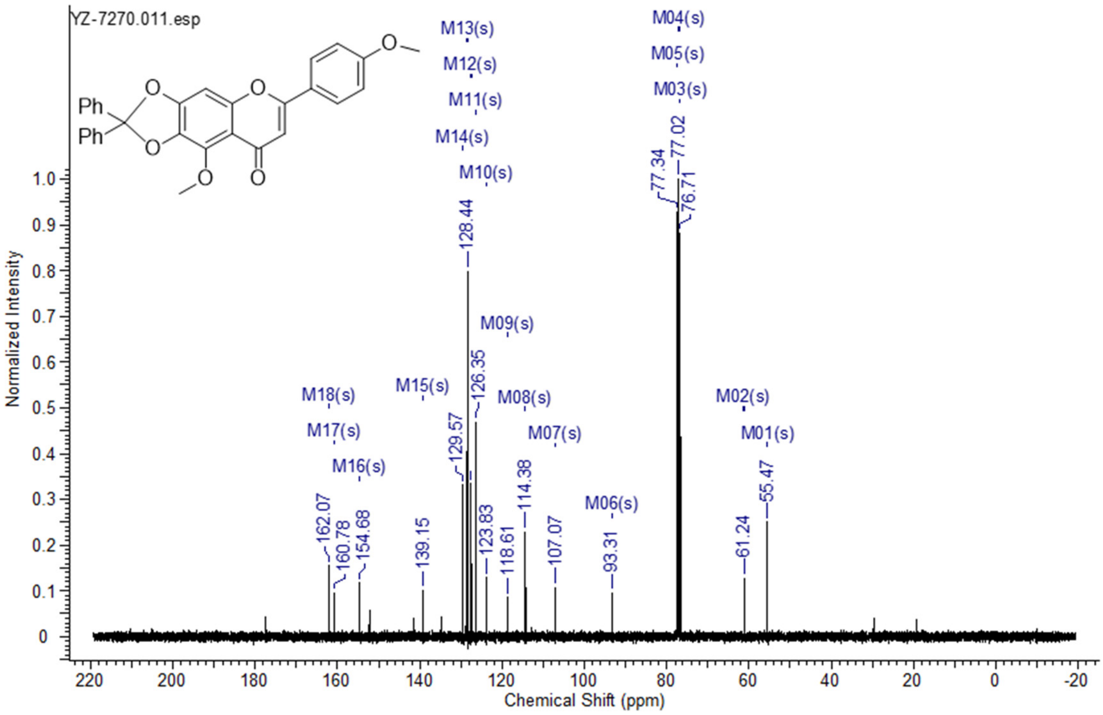

^{1}H NMR spectrum of compound **12a**

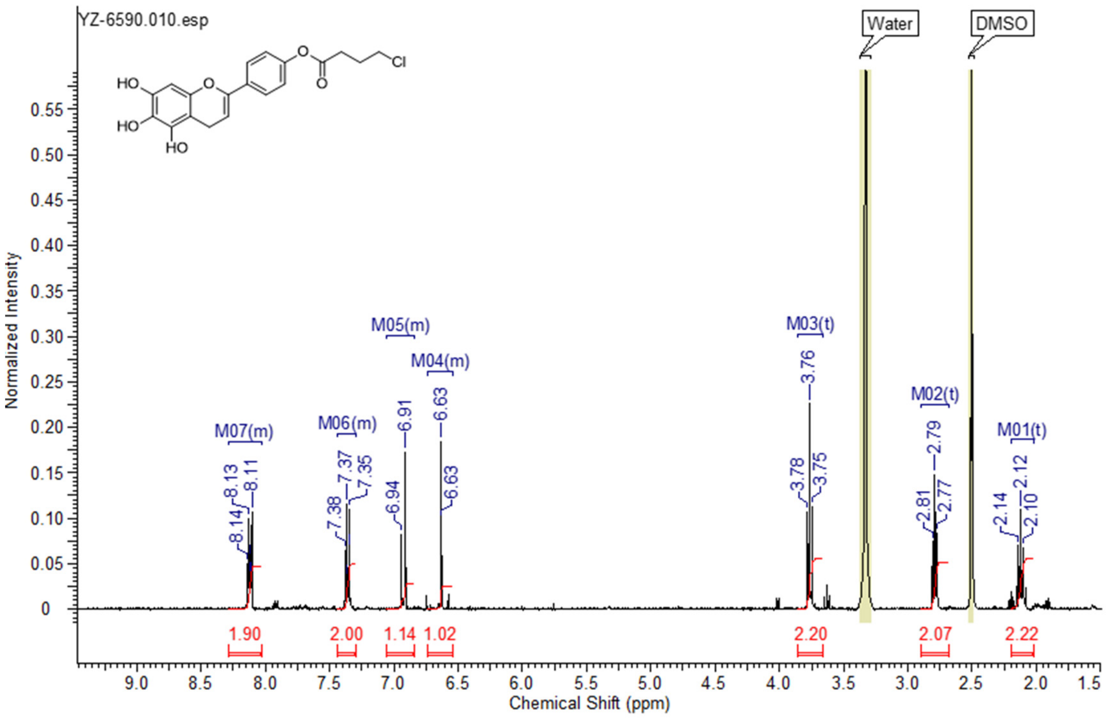

^{13}C NMR spectrum of compound **12a**

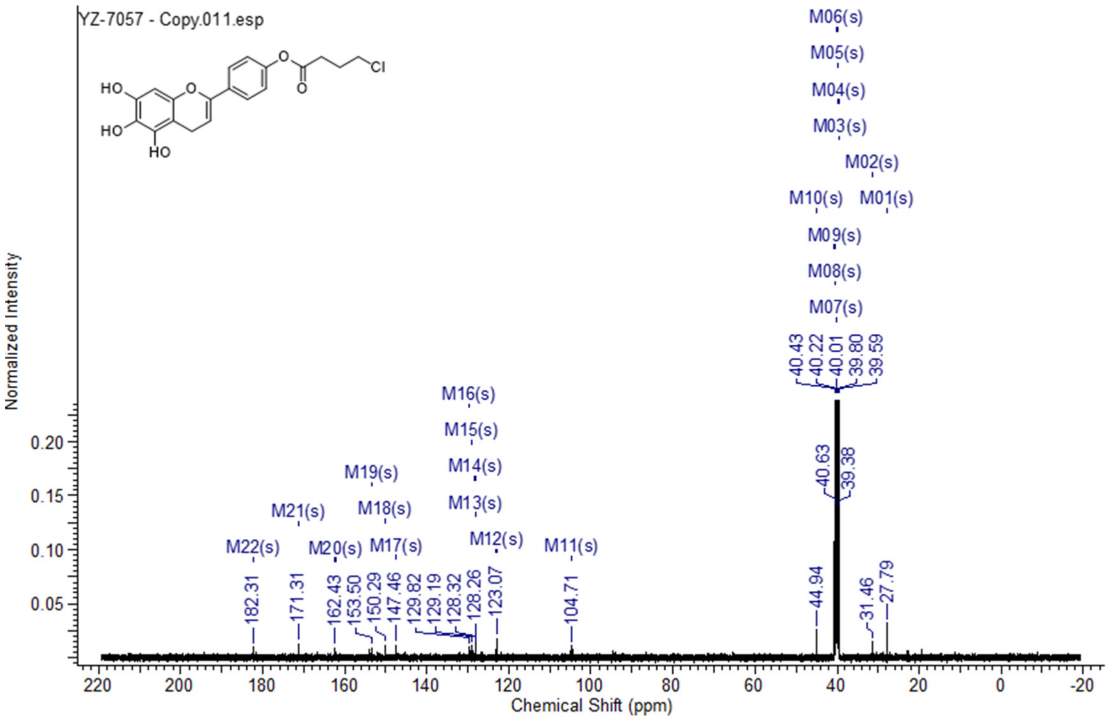

^{1}H NMR spectrum of compound **13a**

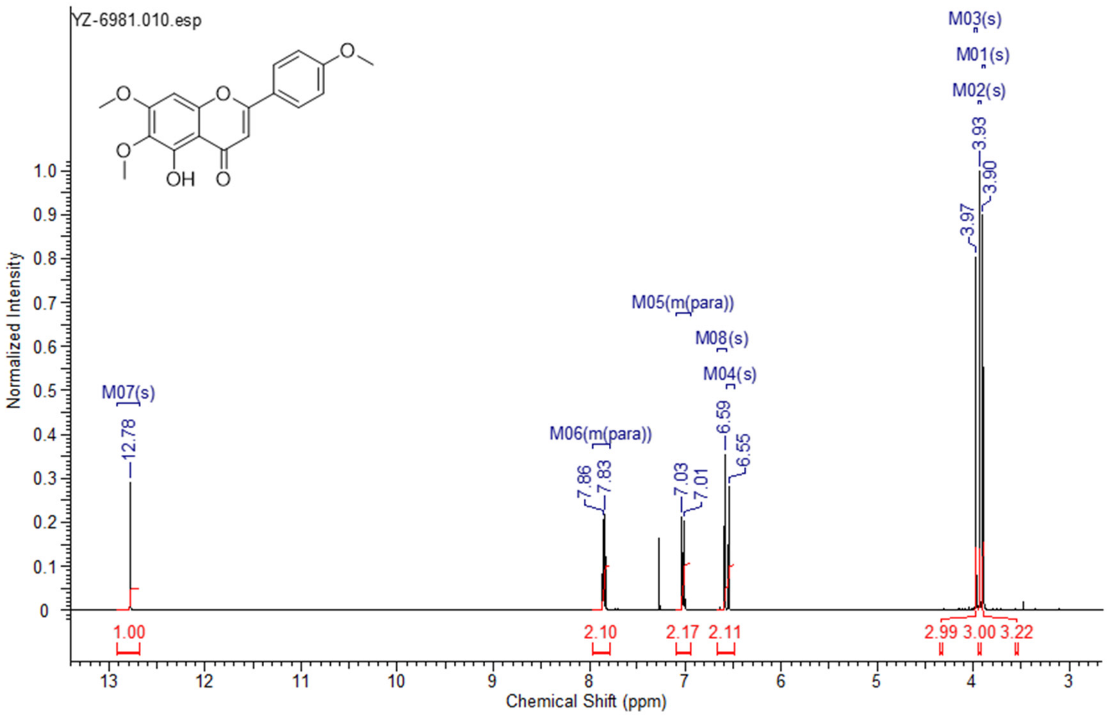

^{13}C NMR spectrum of compound **13a**

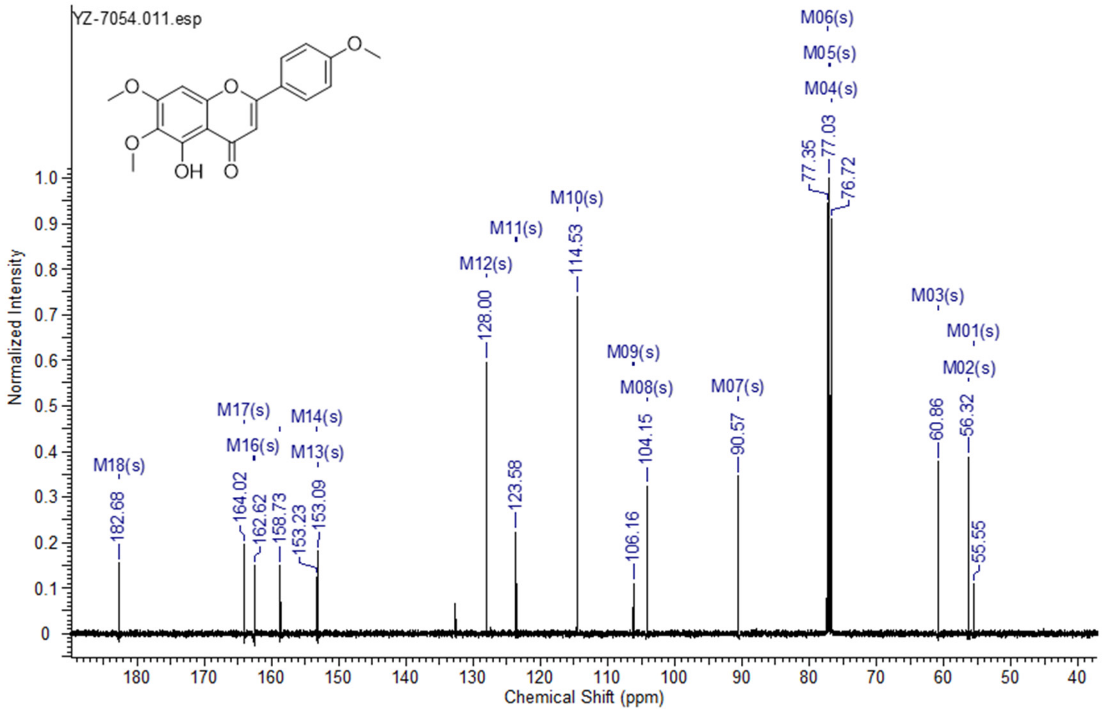

^1H NMR spectrum of compound **13b**

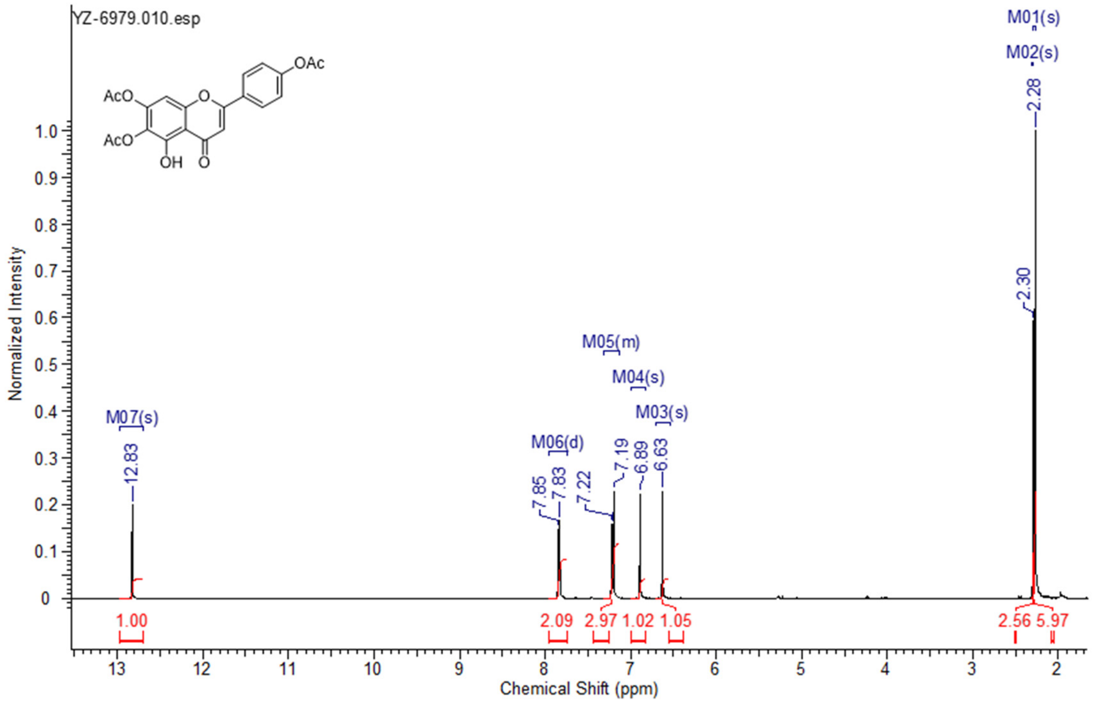

^{13}C NMR spectrum of compound **13b**

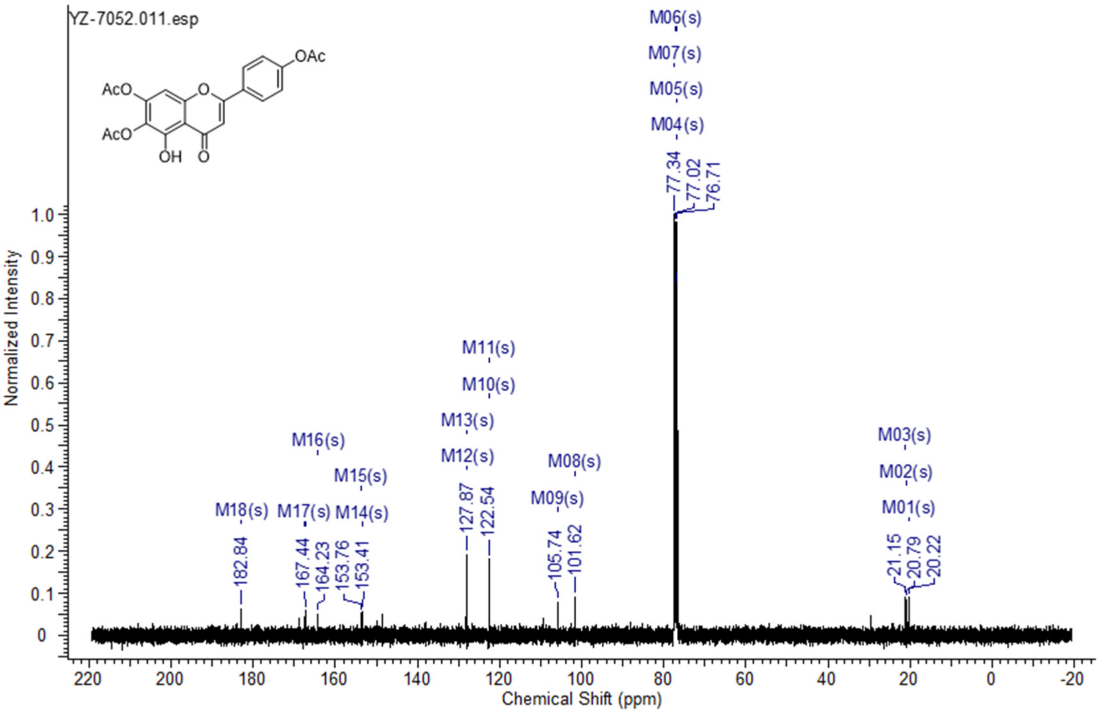

^{1}H NMR spectrum of compound **14a**

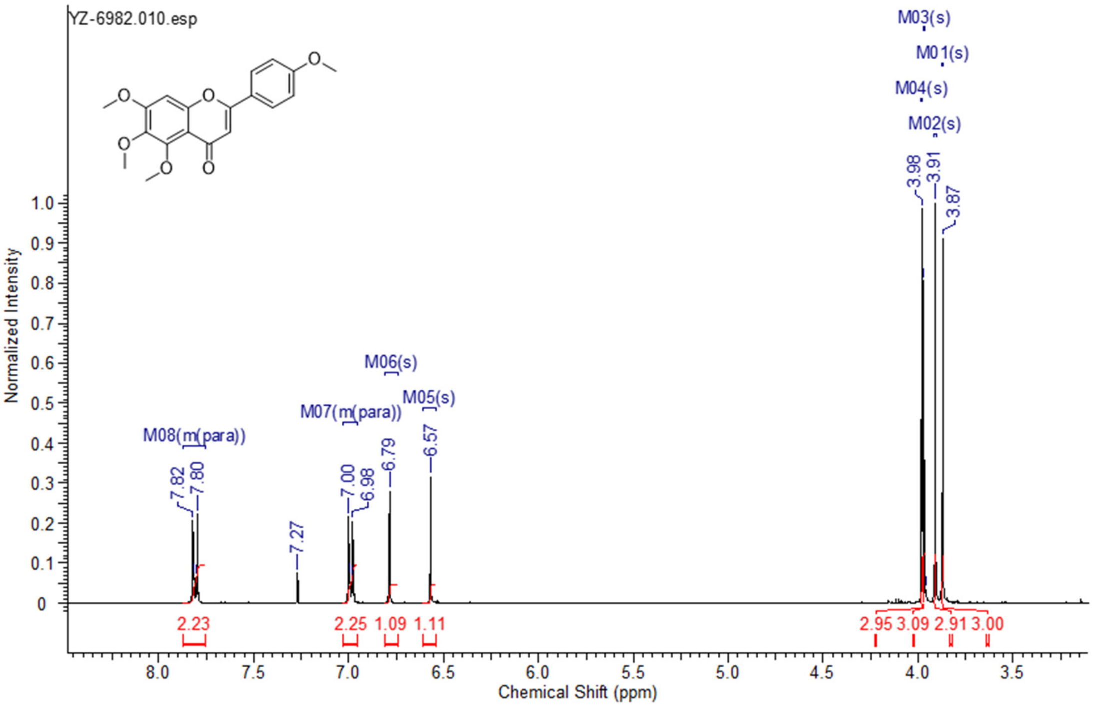

^{13}C NMR spectrum of compound **14a**

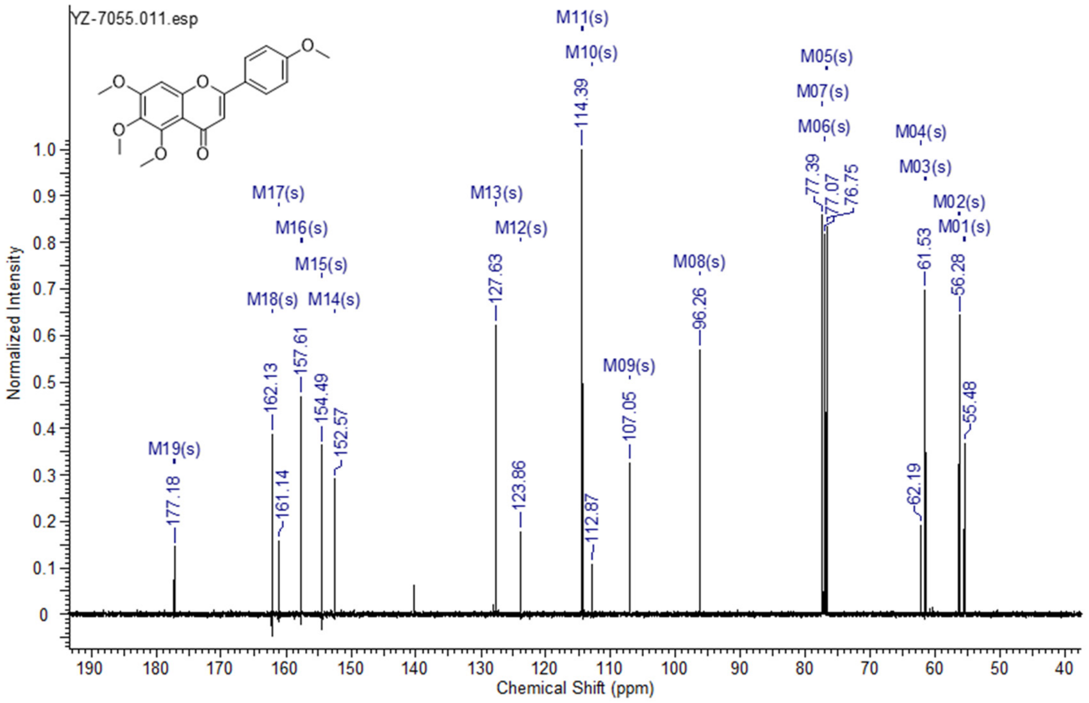

^{1}H NMR spectrum of compound **14b**

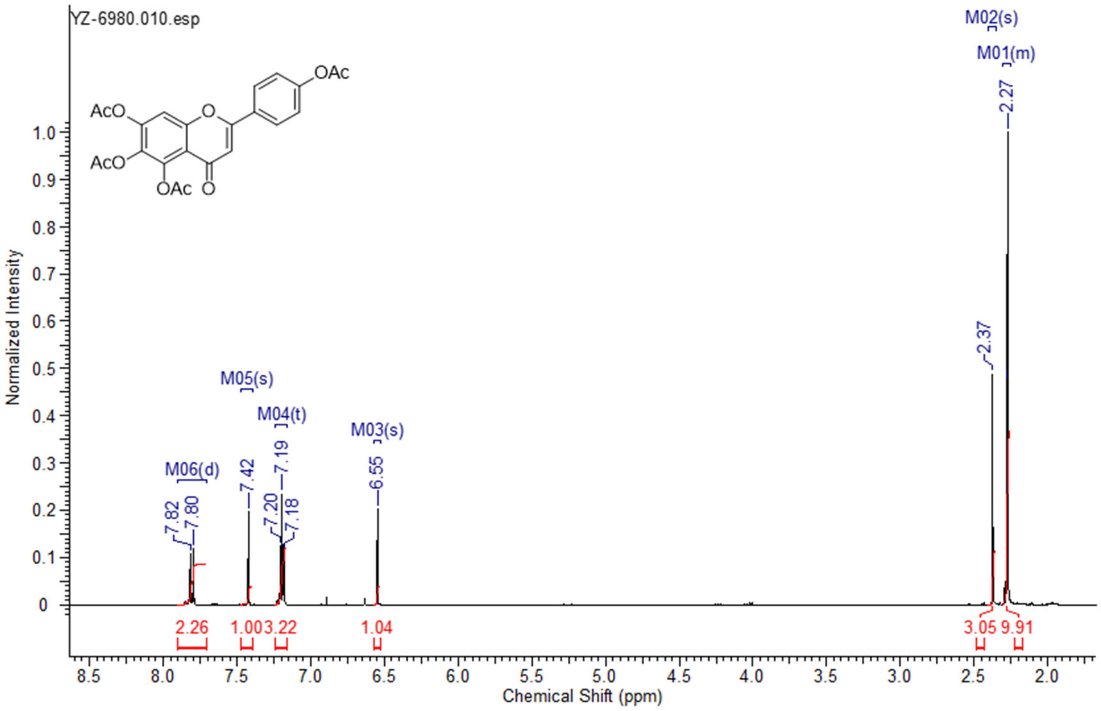

^{13}C NMR spectrum of compound **14b**

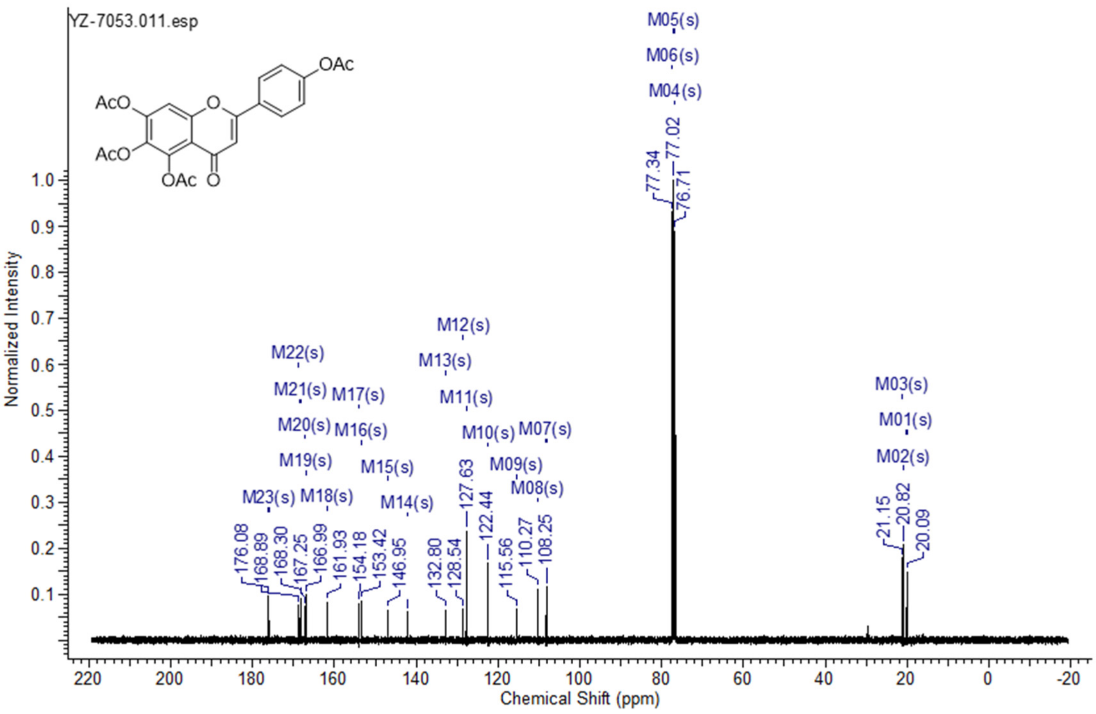

Supplement: Supplementary file 1 [file molecules-28-07417-s001.zip › molecules-2615468-supplementary.pdf]
